# Copper-catalyzed C–S direct cross-coupling of thiols with 5-arylpenta-2,4-dienoic acid ethyl ester†

**DOI:** 10.1039/c8ra05311a

**Published:** 2018-07-27

**Authors:** Rongrong Cai, Zhuoda Zhou, Qianqian Chai, Yueer Zhu, Runsheng Xu

**Affiliations:** Department of Biology and Environment, Jiyang College of Zhejiang A & F University Shaoxing 311800 Zhejiang China 20140041@zafu.edu.cn

## Abstract

A selective copper (Cu)-catalyzed C–S bond direct cross-coupling of thiols with 5-arylpenta-2,4-dienoic acid ethyl ester was developed. Notably, various biologically active 5-phenyl-3-phenylsulfanylpenta-2,4-dienoic acid ethyl ester derivatives were efficiently synthesized under moderate conditions. Finally, a plausible Cu(i)/Cu(iii) reaction mechanism was proposed.

## Abstract 2

A selective copper (Cu)-catalyzed C–S bond direct cross-coupling of thiols with 5-arylpenta-2,4-dienoic acid ethyl ester was developed.

## Graphical abstract

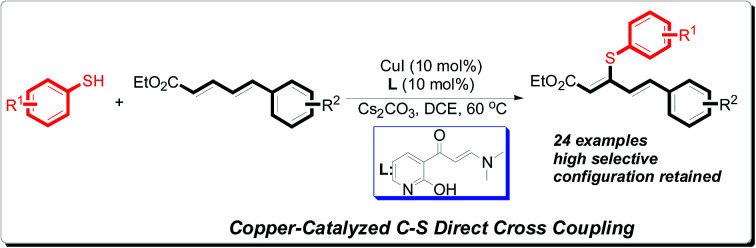

## 1 Introduction

**Scheme 1:**
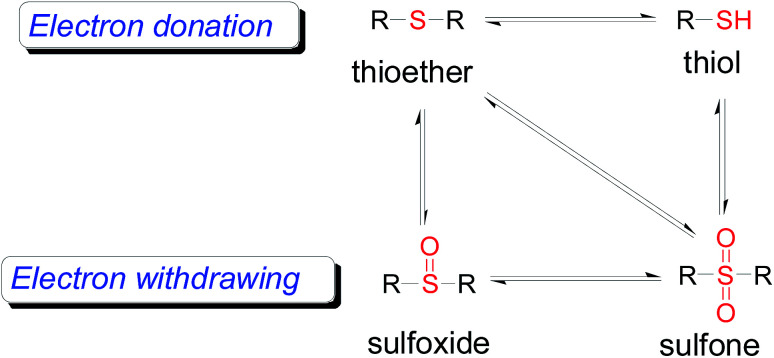
Conversions of sulfur-containing groups.

As one of the most important compounds, organic thioethers are being widely applied in organic synthesis, the pharmaceutical industry, and functional materials.^1^ C–H bonds functionalization has considerably progressed.^2^ In theory, due to larger atomic radius and higher electron density, sulfur has more reactivity and is easy to modify (Scheme 1).^3^ In comparison with the state art of C–C coupling,^4^ acylation^5^ and amination,^6^ C–H bond direct thiolation has been seldom described in the literature. The main reason is that sulfur easily poisons transition metals.^7^ Therefore, developing more efficient strategies for C–H bond thiolation is still required.

Selective C–H bond functionalization, either chemoselective or regioselective, has been long pursued.^8^ The progress on transition-metal catalyzed C–N and C–O cross-coupling has been prompted. However C–H bonds activated by alkenes is rarely reported.^9^ Considering the significance of diversifying synthetic strategies, we focused our interest on sulfur-directed C–H bond functionalization.^10^ 5-Arylpenta-2,4-dienoic acid ethyl esters is an excellent scaffold which ubiquitous in natural biological products, the pharmaceutical chemistry and functionalized materials.^11^ However, the earlier reported synthetic methods have many disadvantages, such as low efficiency, inconvenience, and requiring harsh conditions. Efficient synthetic methods of 5-phenylpenta-2,4-dienoic acid ethyl esters are still required. Due to the functional group tolerance and economic attractiveness, copper catalysts have been extensively used in C–H bonds functionalization.^12^ Herein, we report a selective copper-catalyzed C–S bond direct cross-coupling reaction of thiols with 5-arylpenta-2,4-dienoic acid ethyl ester. In this reaction, various biological activity 5-aryl-3-arylsulfanylpenta-2,4-dienoic acid ethyl ester derivatives were efficiently synthesized under moderate conditions. Finally, a plausible reaction mechanism was proposed.

## 2 Results and discussion

**Scheme 2:**
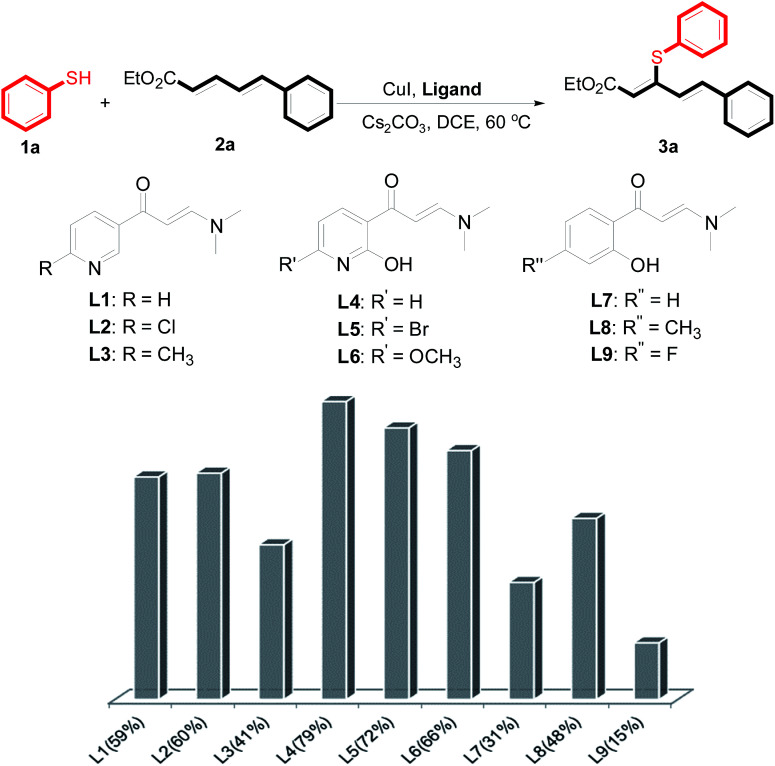
Ligand performance in copper-catalyzed C–S direct cross coupling.

The reaction conditions were screened based on a model reaction of thiophenol 1a with 5-arylpenta-2,4-dienoic acid ethyl ester 2a. These reactions are mainly based on the use of the enaminone ligand, which was previously discovered in our laboratory as an effective ligand for the C–N coupling of Ullmann reactions between aryl halides and various azoles.^10^ At the beginning, various structurally similar enaminone ligands L1–L9 were investigated (Scheme 2). The yields increased by changing the substituent R to R′. Additionally, other enaminone ligands, such as L7, L8, and L9, were observed to be less effective. Analyzing the results, L4 was considered the best ligand.

Furthermore, other reaction parameters were optimized (Table 1). Experimental results demonstrated that the Cu(i) salt resulted in a higher yield than the Cu(ii) salt (entries 1–4). Additionally, the results also demonstrated that the reaction temperature was as an important parameter. The desired product had a 63% yield at 50 °C (entry 8) and a 75% yield at 70 °C (entry 9). Furthermore, the reaction in the absence of the ligand did not occur (entry 11). Finally, the desired product 3a was formed with 81% yield when used the catalyst system L4 with CuI at 60 °C (entry 10).

**Table 1:** Optimization of the model reaction^a^

Entry	Copper salt	Base	1a : 2a	3a^b^(%)
1	Cu(OAc)_2_	Cs_2_CO_3_	1 : 1	15
2	CuSO_4_	Cs_2_CO_3_	1 : 1	42
3	CuBr_2_	Cs_2_CO_3_	1 : 1	NR
4	CuBr	Cs_2_CO_3_	1 : 1	53
5	CuI	Cs_2_CO_3_	1 : 1	79
6	CuI	Na_2_CO_3_	1 : 1.2	NR
7	CuI	K_3_PO_4_	1 : 1.2	41
8	CuI	Cs_2_CO_3_	1 : 1.2	63^c^
9	CuI	Cs_2_CO_3_	1 : 1.2	75^d^
10	CuI	Cs_2_CO_3_	1 : 1.2	81
11	CuI	Cs_2_CO_3_	1 : 1.2	NR^e^

a Unless otherwise noted, reactions conditions were 1a (0.3 mmol), copper source (10 mol%), L5 (10 mol%), Cs_2_CO_3_ (2 equiv.), DCE (4 mL), 60 °C for 24 h, in N_2_.

b Isolated yield.

c At 50 °C.

d At 70 °C.

e Absence of the ligand.

Under the optimized conditions, the reaction scope was next investigated. A wide array of aryl thiols 1 and 5-arylpenta-2,4-dienoic acid ethyl esters 2 were obtained as the productivity with good to excellent yields (Table 2). We found that both the electron-donating and electron-withdrawing aryl thiols 1 reacted smoothly with 5-arylpenta-2,4-dienoic acid ethyl esters 2. The aryl thiols 1 bearing electron-donating groups showed better activity than those with electron-withdrawing groups. 5-Arylpenta-2,4-dienoic acid ethyl esters 2 bearing electron-withdrawing groups showed better activity than those bearing electron-donating groups. The C=C configuration of the 5-arylpenta-2,4-dienoic acid ethyl esters 2 was retained in the corresponding products.

**Table 2:** Copper-catalyzed C–S direct cross-coupling of aryl thiols with 5-arylpenta-2,4-dienoic acid ethyl ester^a^

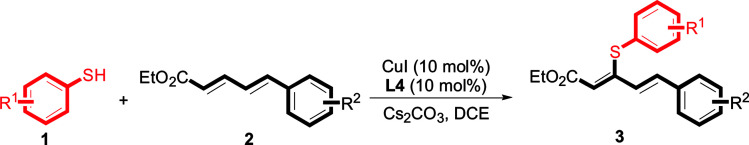				
Entry	1	2	3	Yield^b^ (%)
1	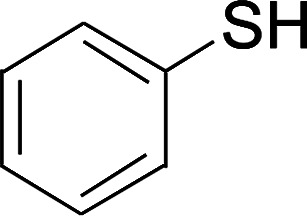	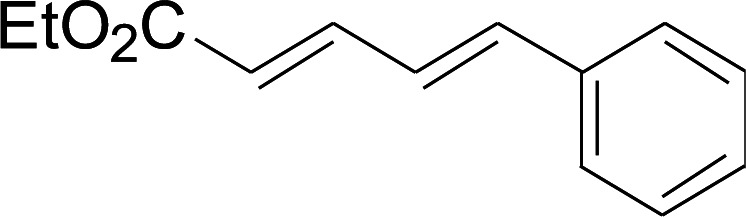	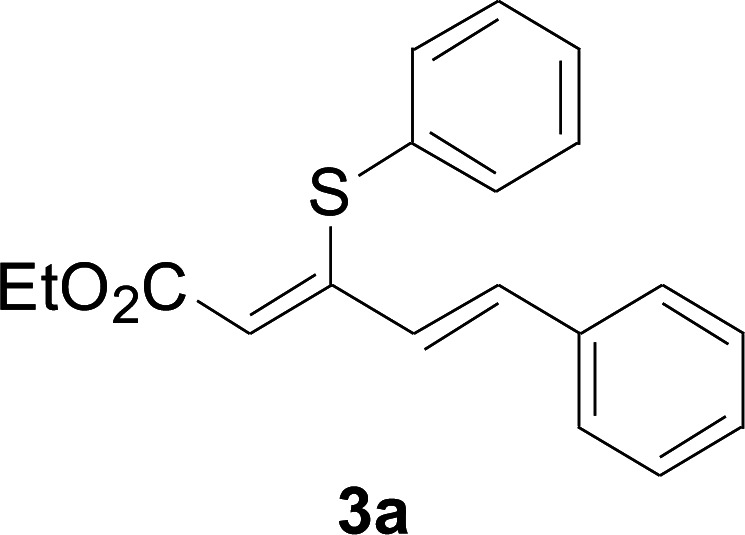	81
2	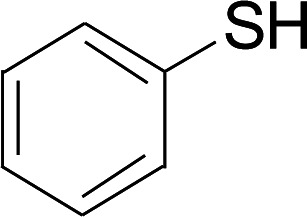	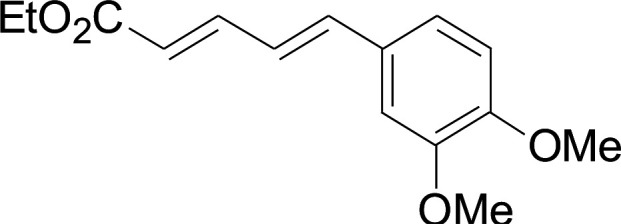	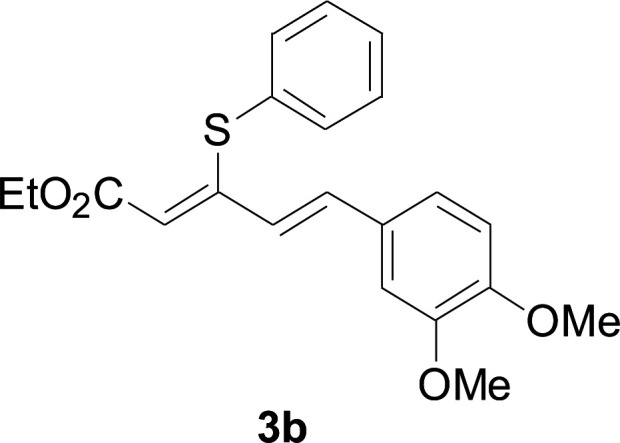	76
3	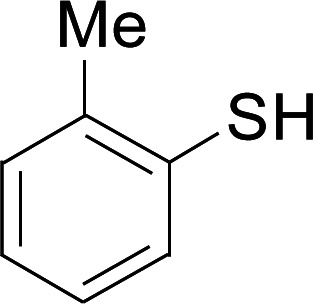	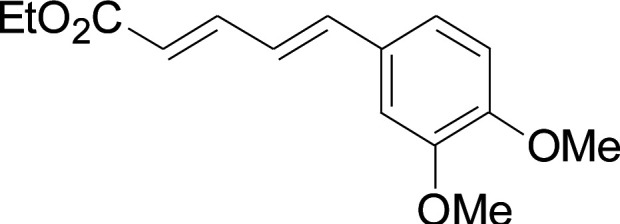	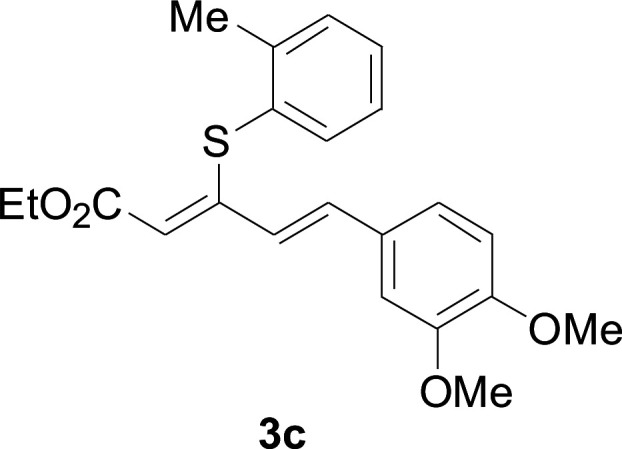	85
4	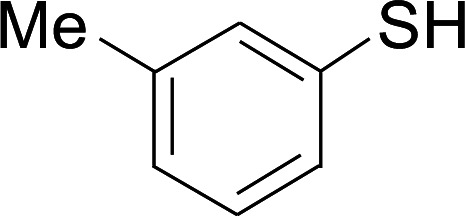	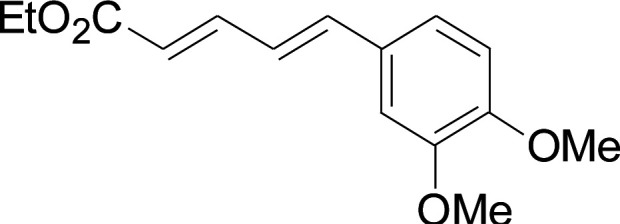	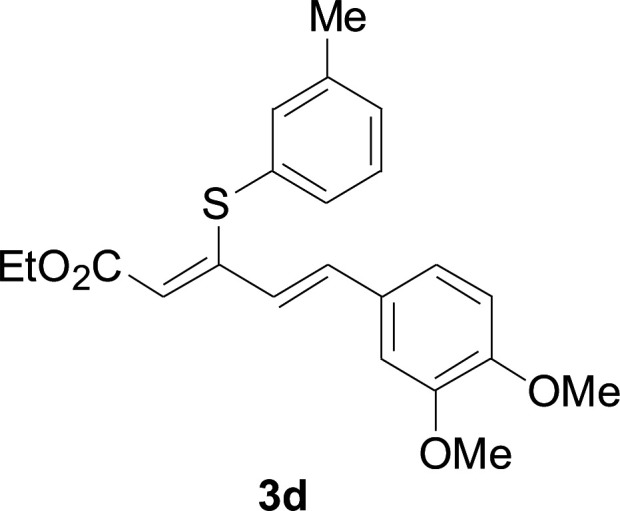	82
5	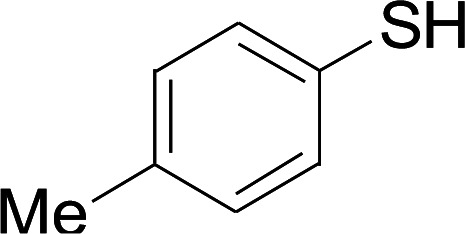	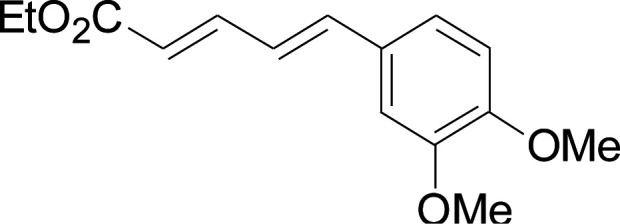	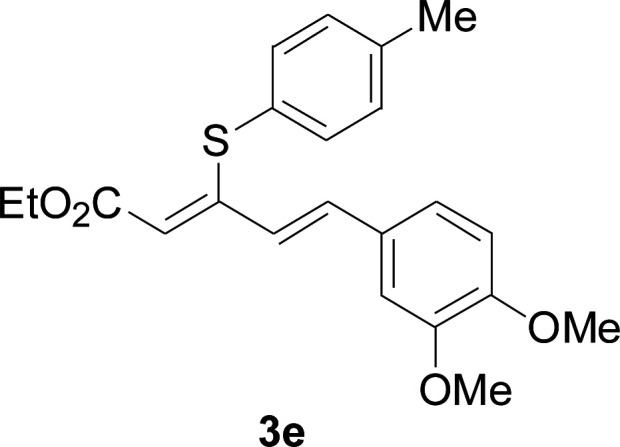	83
6	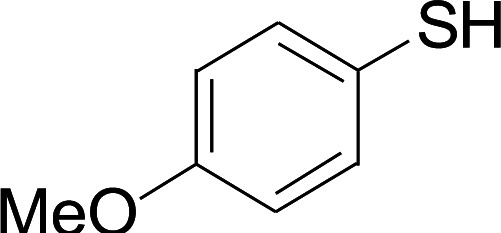	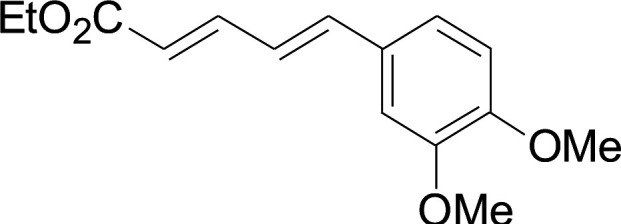	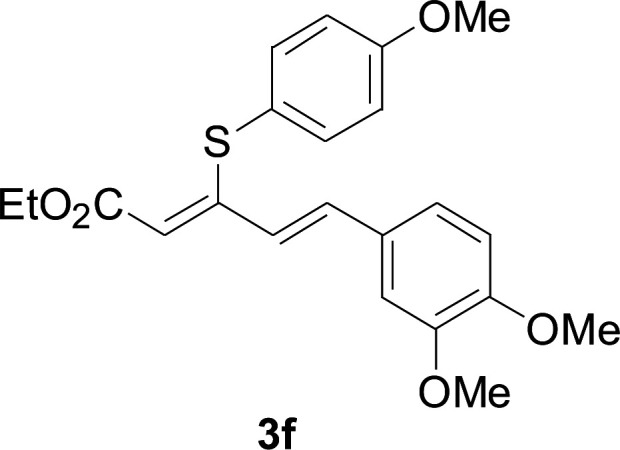	89
7	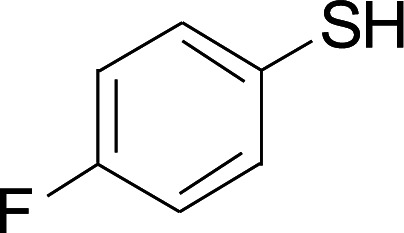	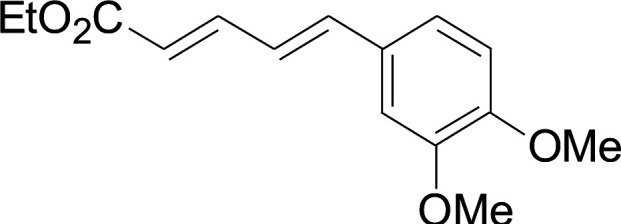	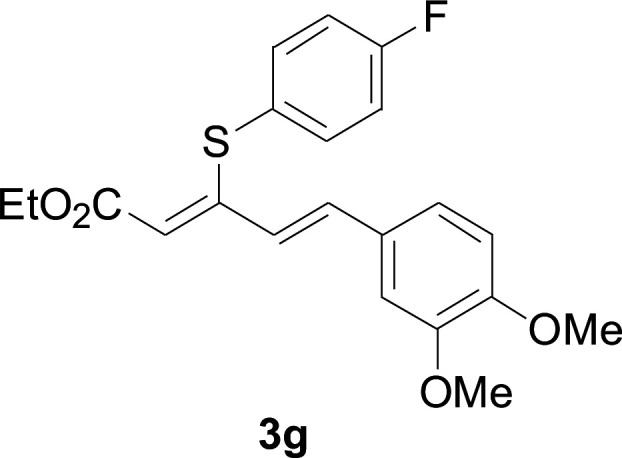	78
8	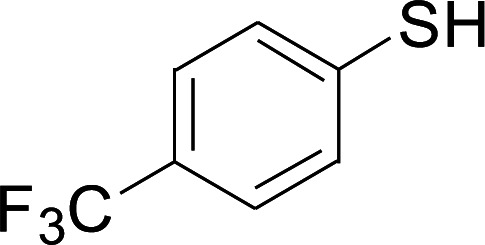	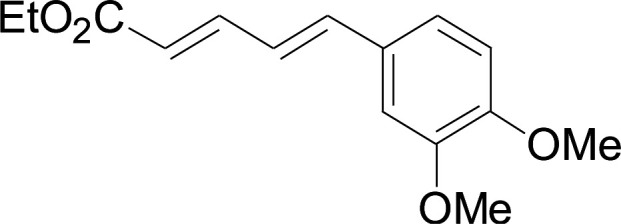	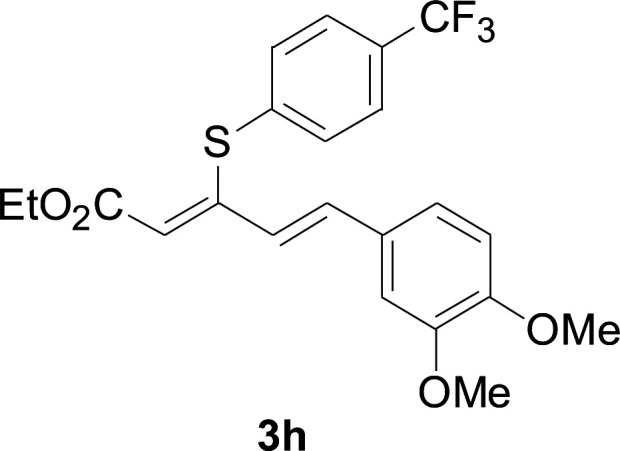	77
9	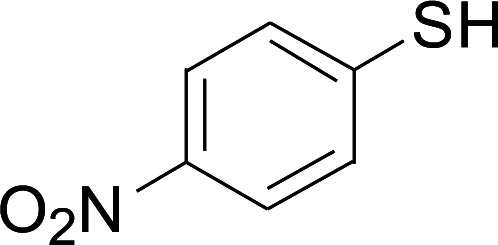	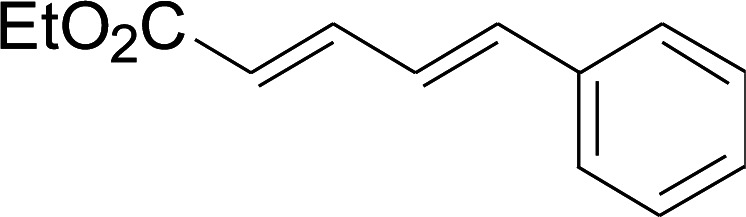	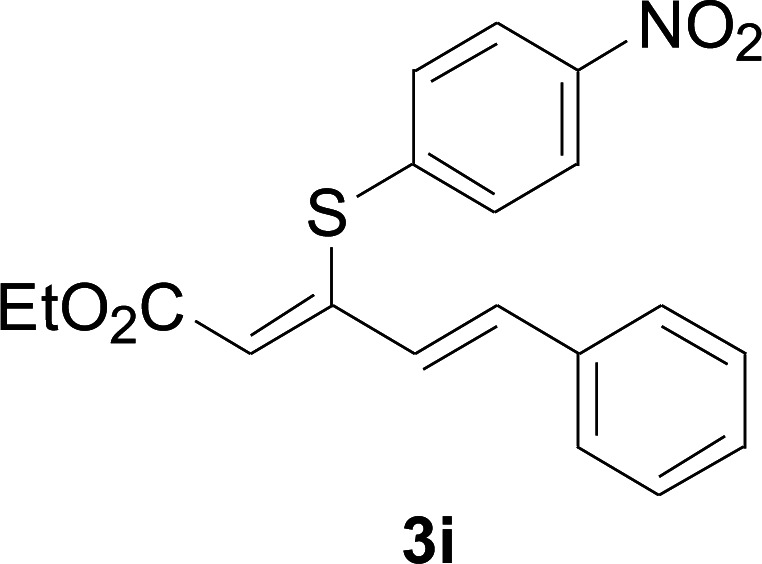	73
10	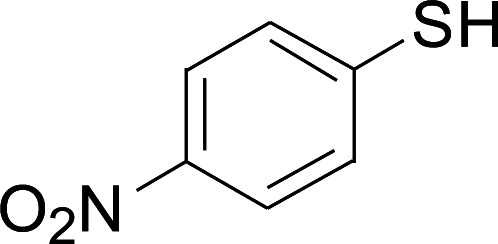	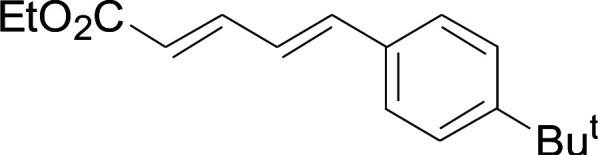	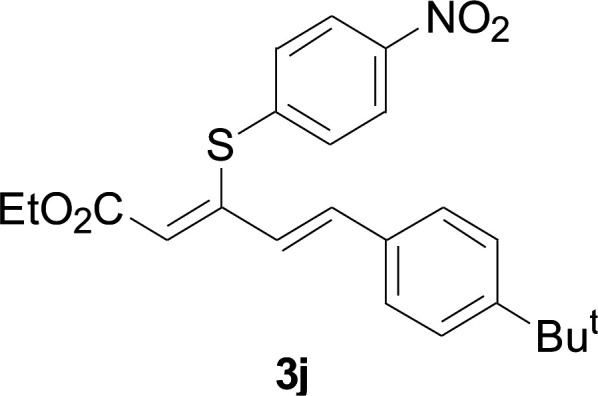	72
11	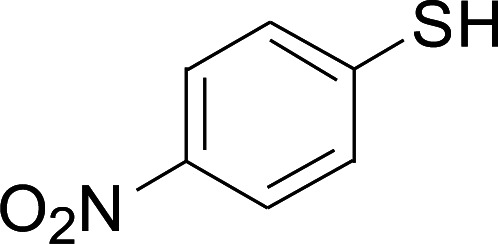	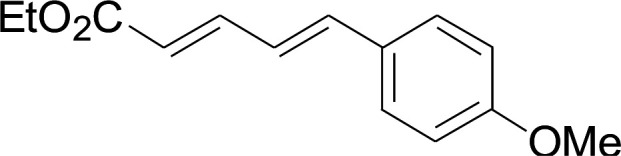	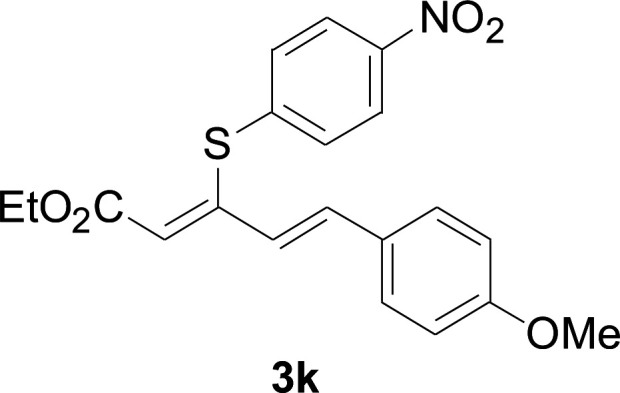	69
12	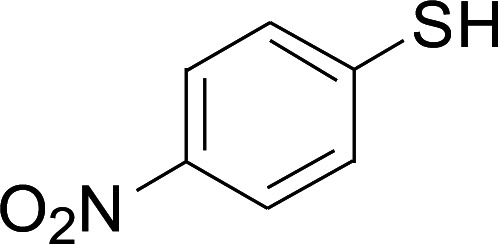	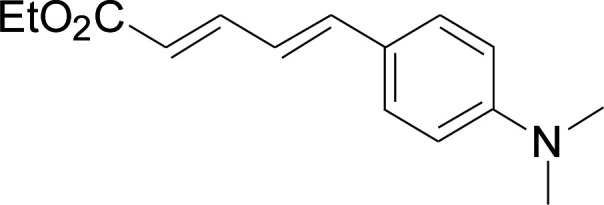	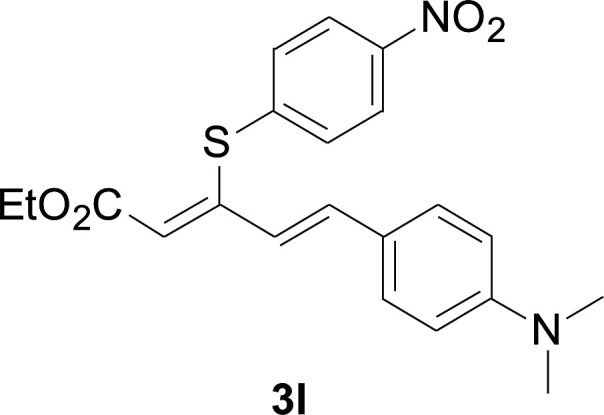	68
13	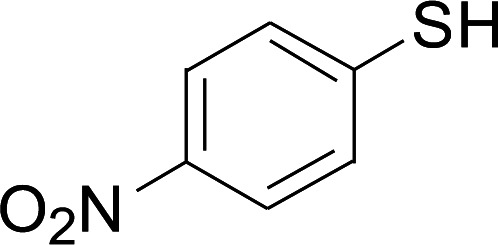	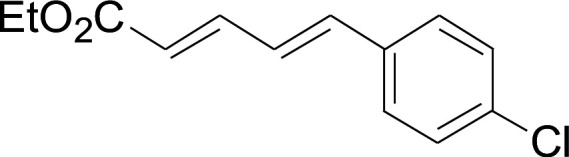	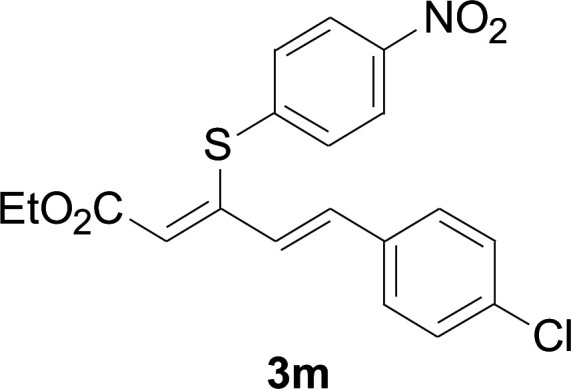	75
14	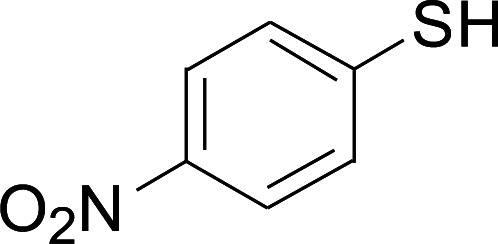	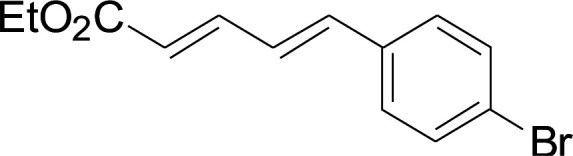	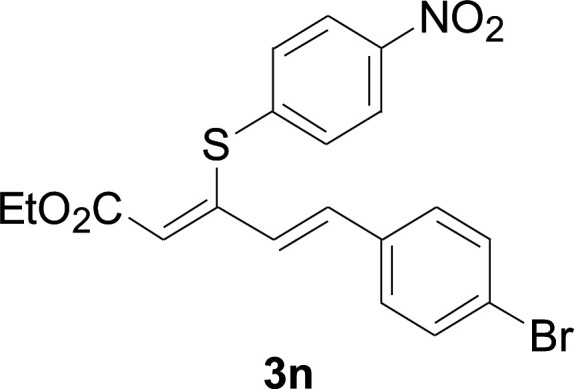	72
15	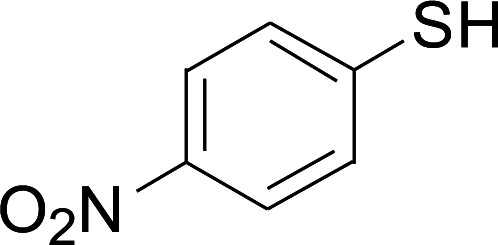	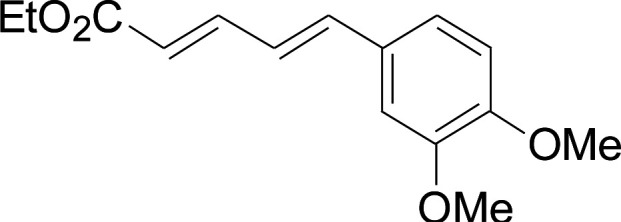	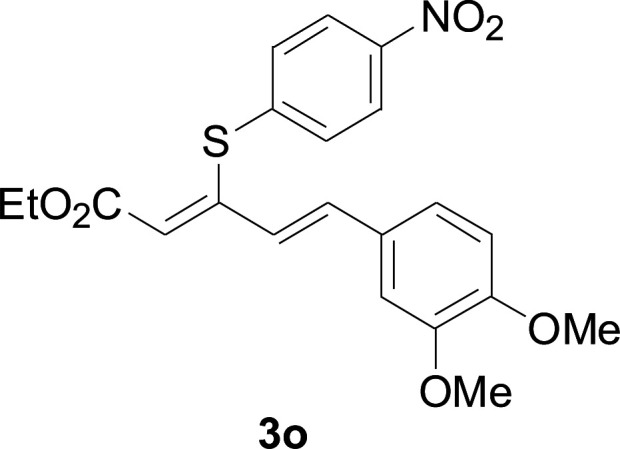	73
16	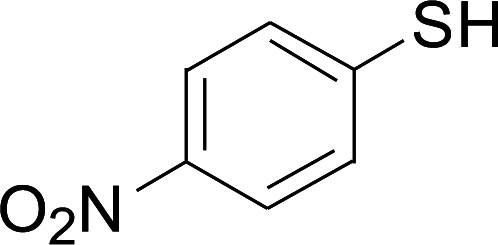	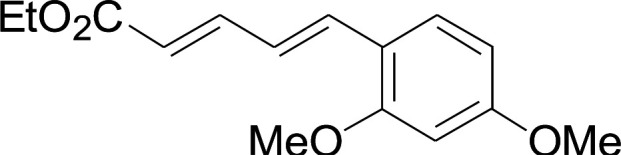	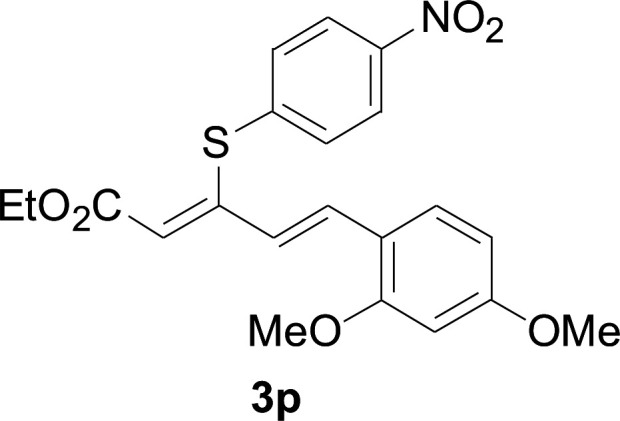	70
17	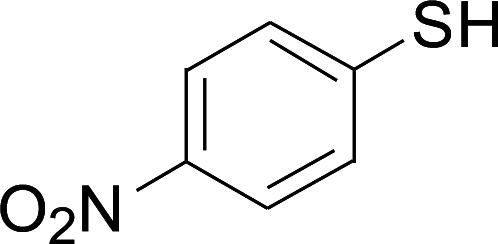	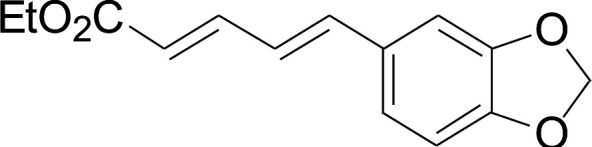	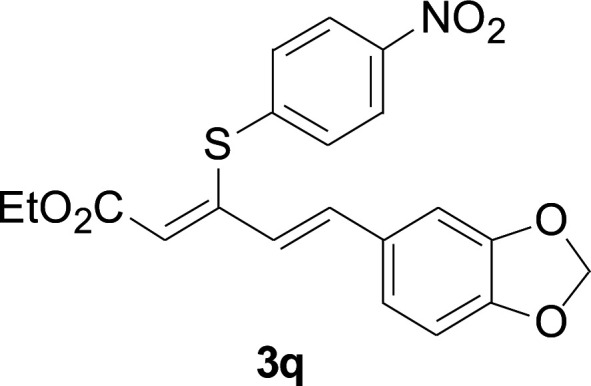	78
18	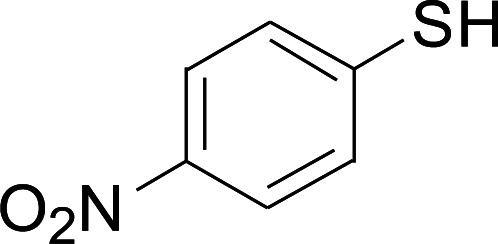	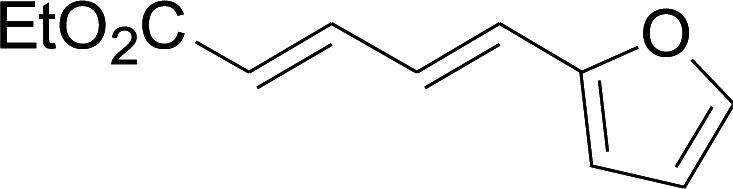	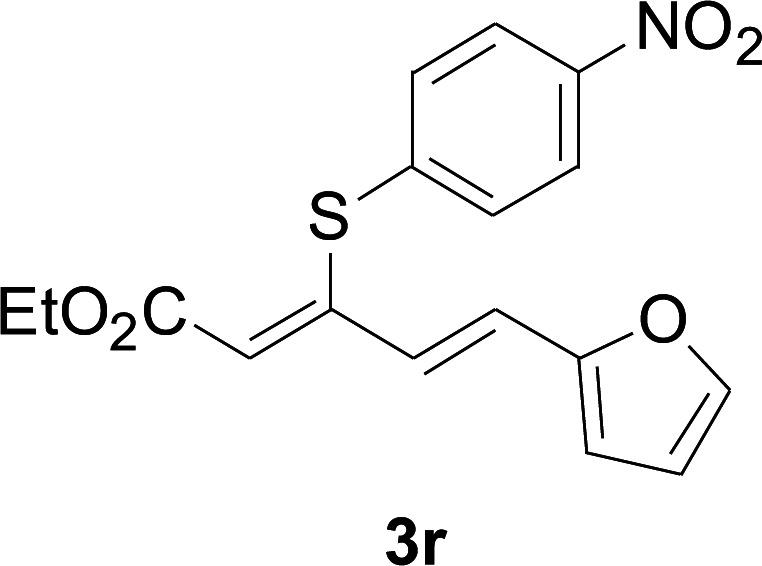	81
19	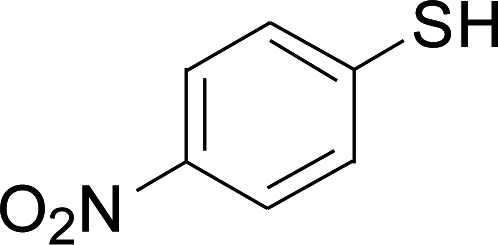	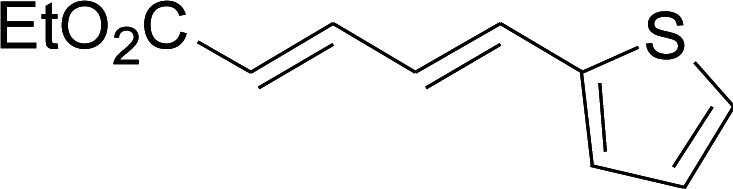	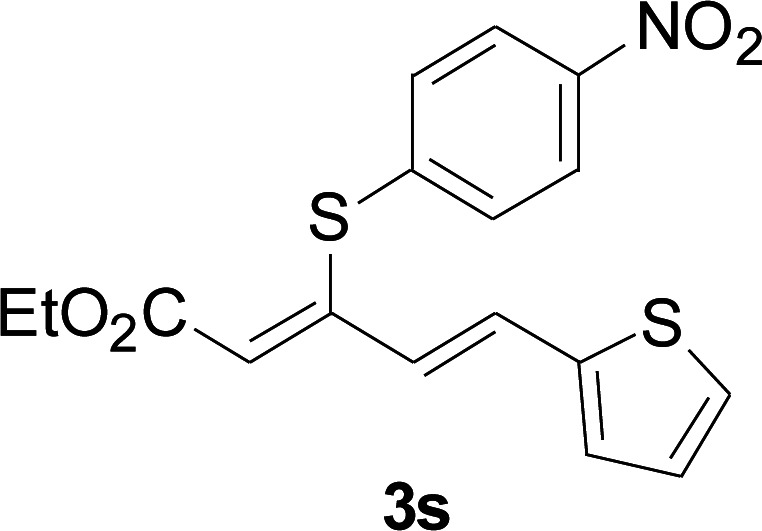	84

a Reactions conditions: 1 (0.3 mmol), 2 (0.36 mmol), CuI (10 mol%), L4 (10 mol%), Cs_2_CO_3_ (2 equiv.), in DCE (4 mL) at 60 °C for 24 h, in N_2_.

b Isolated yield.

Next, we focused on other thiols (Table 3). Aliphatic thiols worked well in this reaction. The corresponding products were isolated with 76–88% yields. The C=C configuration of the 5-phenylpenta-2,4-dienoic acid ethyl esters 2 were also retained in corresponding products.

**Table 3:** Copper-catalyzed C–S direct cross-coupling of aliphatic thiols with 5-arylpenta-2,4-dienoic acid ethyl ester^a^

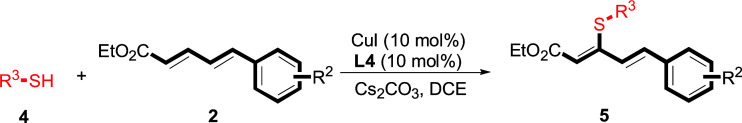				
Entry	1	4	5	Yield^b^ (%)
1	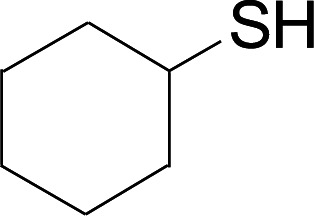	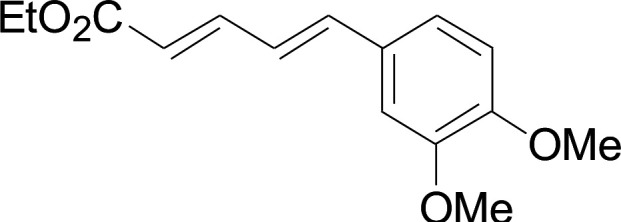	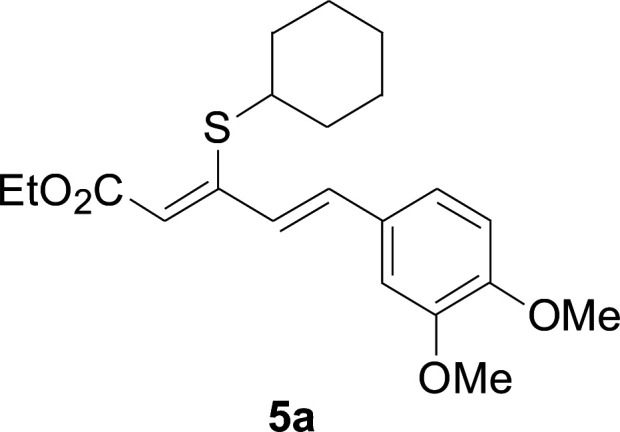	88
2	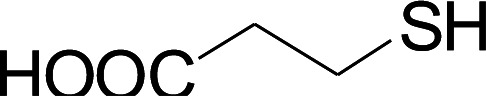	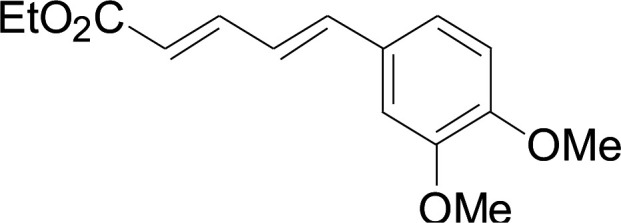	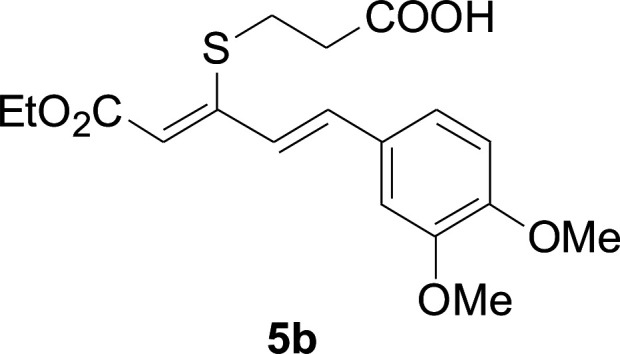	81
3	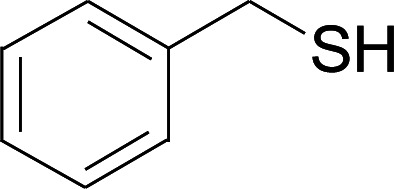	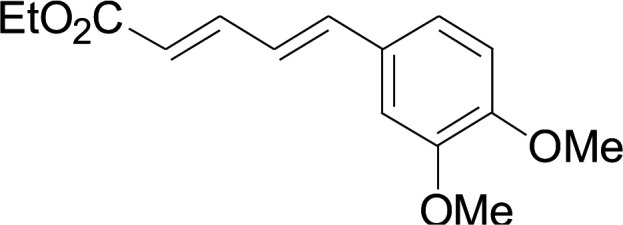	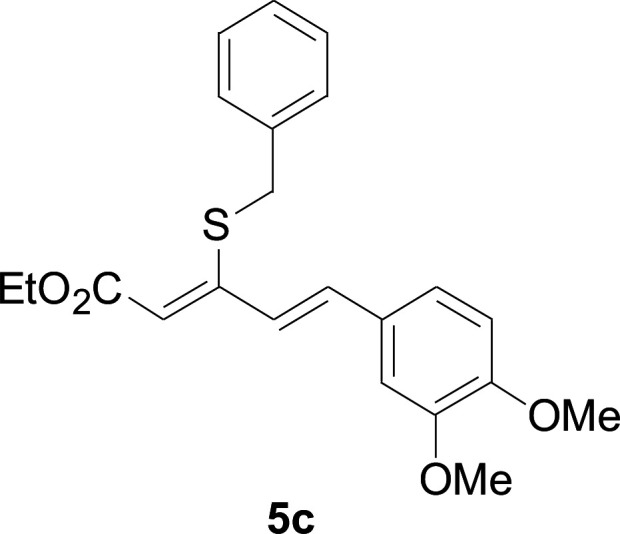	76
4	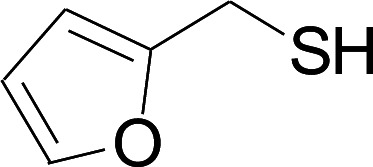	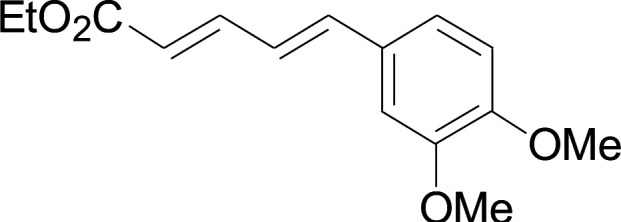	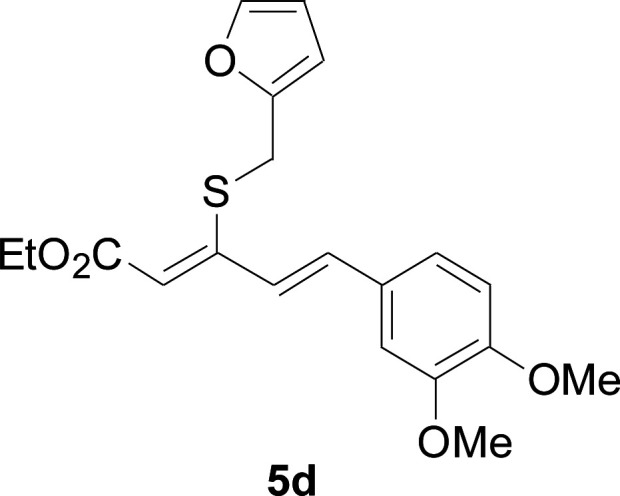	85
5	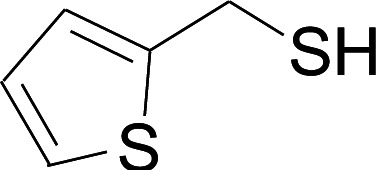	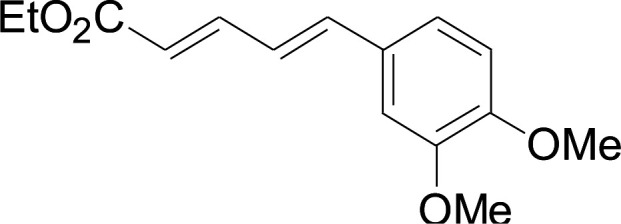	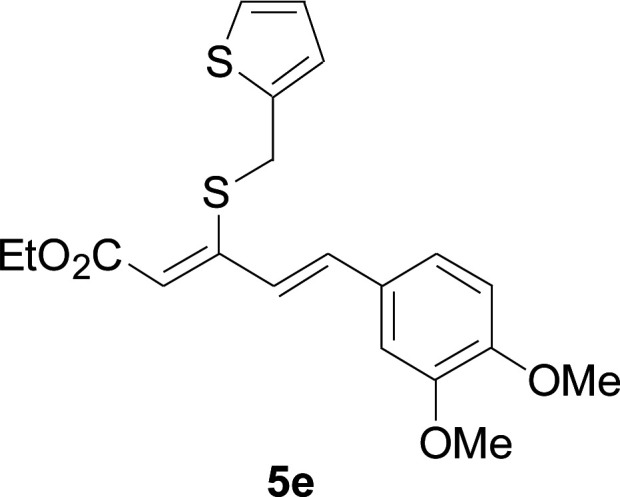	83

a Reactions conditions: 4 (0.3 mmol), 2 (0.36 mmol), CuI (10 mol%), L4 (10 mol%), Cs_2_CO_3_ (2 equiv.), DCE (4 mL), 60 °C for 24 h, in N_2_.

b Isolated yield.

**Scheme 3:**
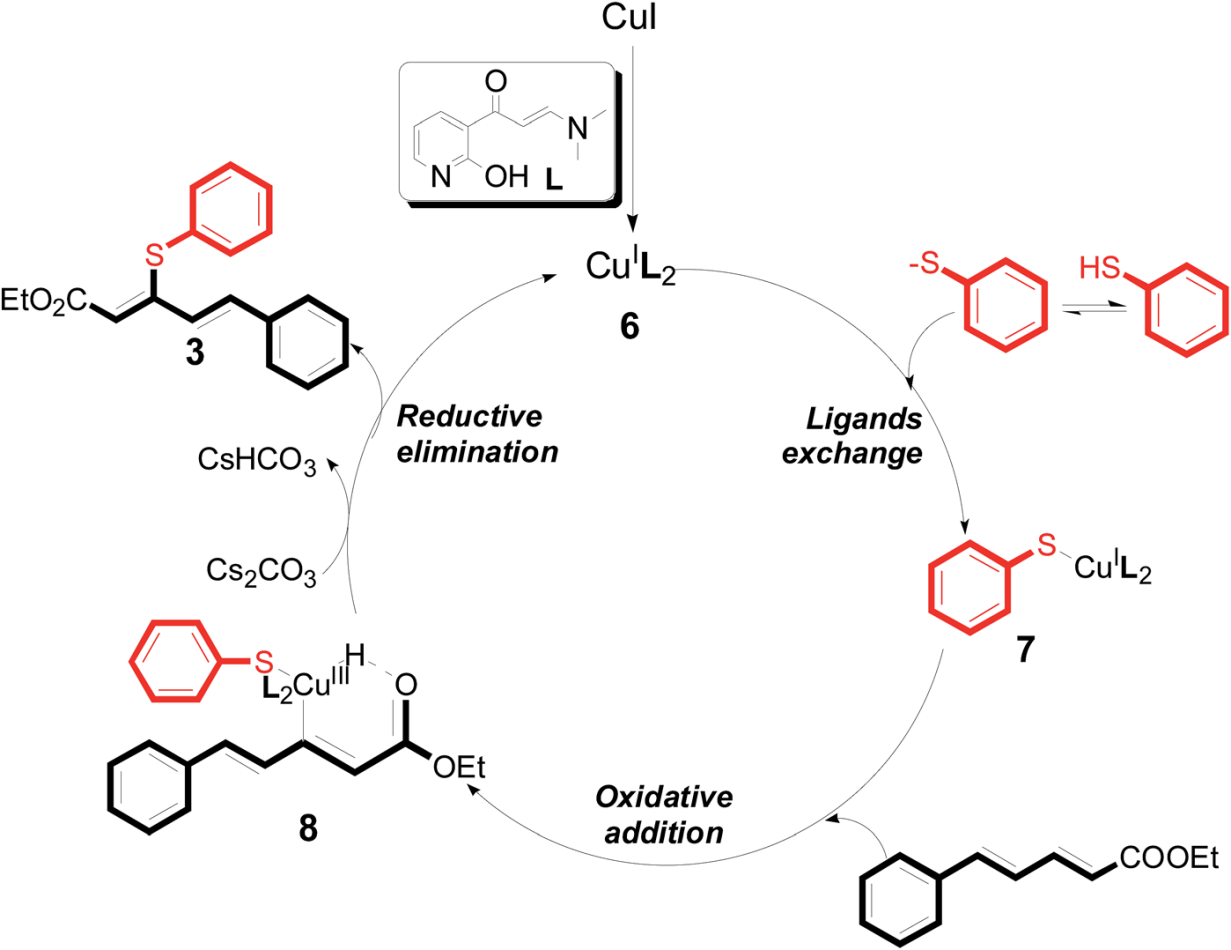
A plausible reaction mechanism.

There were two reasons of the ester group essential for the regioselectivity in those reactions. First, ethyl ester was more common and more meaningful than other alkyl esters in the field of synthetic industry and functional materials. Secondly, ethyl ester had better coordination ability than other alkyl ester. Based on the above results, a reaction mechanism was proposed (Scheme 3). After the coordination of CuI with L4, a corresponding intermediate 6 was generated.^13^ Next, an intermediate 7 was formed from intermediate 6 with aryl thiols through a ligand exchange step. Next, intermediate 7 reacted with 5-arylpenta-2,4-dienoic acid ethyl esters to produce intermediate 8*via* an intermolecular oxidative addition. Finally, intermediate 8 furnished the desired product 3 and concomitantly generated intermediate 6, which re-entered the catalytic cycle. Furthermore, the specific reaction mechanism is still under study *via* high-resolution electrospray ionisation mass spectrometry (HR-ESI-MS) and density functional theory (DFT).

## 3 Conclusions

In conclusion, a selective copper-catalyzed C–S bond direct cross-coupling of thiols with 5-arylpenta-2,4-dienoic acid ethyl esters was developed. Using this methodology, various biological activity 5-aryl-3-phenylsulfanylpenta-2,4-dienoic acid ethyl ester derivatives were efficiently synthesized. The process used inexpensive catalysts and under mild conditions. The reaction mechanism of the Cu(i)/Cu(iii) catalysis cycle was proposed.

## 4 Experimental section

### 4.1. General procedure for preparation of L1–L10

Dimethylformamide dimethylacetal (DMFDMA) (10 mmol, 1.19 g) and 1-pyridin-3-yl-ethanone (10 mmol, 1.21 g) were dissolved in *p*-xylene (2 mL). And the mixture was refluxed during a period of 5 to 12 hours, during which time the formation of yellow precipitate. The precipitate was filtered out and washed with petroleum ether three times. The solid was vacuum-dried, and 1.65 g (yield 94%) of a yellow solid was obtained L1 3-dimethylamino-1-pyridin-3-yl-propenone. ^1^H NMR (500 MHz, d^6^-DMSO): *δ* 14.51 (s, 1 H), 7.92–7.90 (t, *J* = 7.5 Hz, 2 H), 7.37–7.34 (s, *J* = 7.8 Hz, 1 H), 6.83 (d, *J* = 2.0 Hz, 1 H), 5.98–5.95 (d, *J* = 12 Hz, 1 H), 3.19 (s, 3 H), 2.98 (s, 3 H); ^13^C NMR (125 MHz, d^6^-DMSO): *δ* 191.1, 163.6, 134.9, 129.9, 121.2, 119.1, 118.7, 90.4, 46.1, 38.6.

### 4.2. General procedure for preparation of 3 and 5

A mixture of benzenethiol 1a (33.0 mg, 0.3 mmol), 5-phenylpenta-2,4-dienoic acid ethyl ester 2a (72.7 mg, 0.36 mmol), CuI (5.7 mg, 10 mol%), 3-dimethylamino-1-(2-hydroxypyridin-3-yl)-propenone L4 (5.8 mg, 10 mol%) and Cs_2_CO_3_ (195.6 mg, 2 equiv.) in DMSO (4 mL) was stirred under a N_2_ atmosphere. After the reaction mixture was stirred at 60 °C for 24 h, it was allowed to cool to ambient temperature. Then the mixture was quenched with saturated salt water (10 mL), and the solution was extracted with ethyl acetate (3 × 10 mL). The organic layers were combined and dried by sodium sulfate and concentrated *in vacuo*. The pure product 5-phenyl-3-phenylsulfanylpenta-2,4-dienoic acid ethyl ester 3a (75.3 mg, 81% yield) was obtained by flash column chromatography on silica gel.

#### 4.2.1 5-Phenyl-3-phenylsulfanylpenta-2,4-dienoic acid ethyl ester (3a)

75.3 mg, 81% yield; yellow soild; mp 111–113 °C; ^1^H NMR (500 MHz, CDCl_3_): *δ* 8.41 (dd, 1 H, *J* = 15.9 Hz, 0.8 Hz), 8.20 (d, 2 H, *J* = 8.9 Hz), 7.57 (d, 2 H, *J* = 8.9 Hz), 7.50–7.55 (m, 2 H), 7.30–7.43 (m, 4 H), 6.73 (m, 1 H), 5.90 (m, 1 H), 4.23 (q, 2 H), 1.35 (t, 3 H); ^13^C NMR (125 MHz, CDCl_3_): 160.8 (C), 145.9 (C), 142.8 (C), 140.7 (C), 136.9 (CH), 133.7 (CH), 129.0 (CH), 127.5 (CH), 126.8 (CH), 125.8 (CH), 122.4 (CH), 121.3 (CH), 120.1 (CH), 58.6 (CH_2_), 11.3 (CH_3_); ESI-HRMS *m*/*z*: calcd for C_19_H_19_O_2_S^+^ [M + H]^+^: 311.1100; found 311.0997.

#### 4.2.2 3-Cyclohexylsulfanyl-5-(3,4-dimethoxyphenyl)penta-2,4-dienoic acid ethyl ester (5a)

99.4 mg, 88% yield; yellow oil; ^1^H NMR (500 MHz, CDCl_3_): *δ* 8.19 (dd, 1 H, *J* = 16.1, 0.8 Hz), 7.25 (d, 1 H, *J* = 16.1 Hz), 7.05–7.13 (m, 2 H), 6.84 (d, 1 H, *J* = 12.3 Hz), 5.71 (s, 1 H), 4.19 (q, 2 H), 3.92 (s, 3 H), 3.90 (s, 3 H), 3.14–3.25 (m, 1 H), 2.02–2.14 (m, 2 H), 1.76–1.86 (m, 2 H), 1.60–1.70 (m, 1 H), 1.37–1.54 (m, 5 H), 1.26–1.36 (m, 3 H); ^13^C NMR (125 MHz, CDCl_3_): *δ* 165.5 (C), 154.0 (C), 150.0 (C), 149.1 (C), 136.1 (CH), 129.5 (C), 123.1 (CH), 121.4 (CH), 111.4 (CH), 111.1 (CH), 109.7 (CH), 59.9 (CH_2_), 55.94 (CH_3_), 55.91 (CH_3_), 44.0 (CH), 32.7 (CH_2_), 26.0 (CH_2_) 25.9 (CH_2_), 14.5 (CH_3_); ESI-HRMS *m*/*z*: calcd for C_21_H_29_O_4_S^+^ [M + H]^+^: 377.1781; found 377.1778.

## Conflicts of interest

There are no conflicts to declare.

## Supplementary Material

RA-008-C8RA05311A-s001
