# Supplementary material for: Copper-catalyzed C–S direct cross-coupling of thiols with 5-arylpenta-2,4-dienoic acid ethyl ester
Source: RSC Adv. 2018 Jul 27;8(47):26828–36. doi: 10.1039/c8ra05311a (PMC9083087; doi:10.1039/c8ra05311a)
Supplement: RA-008-C8RA05311A-s001 [file RA-008-C8RA05311A-s001.pdf]

Electronic Supplementary Material (ESI) for RSC Advances.
This journal is © The Royal Society of Chemistry 2018

Electronic supplementary information

Copper-catalyzed C-S direct cross-coupling of thiols with 5-arylpenta-2, 4-dienoic acid ethyl ester

Rong-Rong Cai, Zhuo-Da Zhou, Qian-Qian Chai, Yue-Er Zhu and Run-Sheng Xu*

Department of Biology and Environment, Jiyang College of Zhejiang A＆F University, Shaoxing 311800, Zhejiang China

E-mail: 20140041@zafu.edu.cn

Contents

Contents.. 1

Experimental Details.. 2

Analytical Datas.. 3

Spectrums...14

Experimental Details

General information

All reagents used in the experiment were obtained from commercial sources and used without further purification. Unless otherwise noted, all reactions were carried out at N_2 atmosphere. Analytical thin layer chromatography (TLC) employed glass 0.25 mm silica gel plates. All NMR spectra were recorded on Bruker AVANCE DMX-500 spectrometry at 500 MHz and 125 MHz for ^{1}H and ^{13}C NMR in $CDCl_3$, respectively. Unless otherwise noted, ^{1}H and ^{13}C chemical shifts are referenced to at $CHCl_3$ at 7.24 ppm and 77.0 ppm. Multiplicities are reported using the following abbreviations: s = singlet, d = doublet, t = triplet, q = quartet, m = multiplet, br = broad resonance. The ^{1}H NMR spectra were reported in delta (δ) units, parts per million (ppm) downfield from the internal standard. Coupling constants are reported in Hertz (Hz). Mass spectras were performed on a Bruker Esquire 3000plus mass spectrometer equipped with ESI interface and ion trap analyzer. The ESI-HRMS were tested on Bruker 7-tesla FT-ICR MS equipped with an electrospray source.

General procedure for preparation of L1-L10:

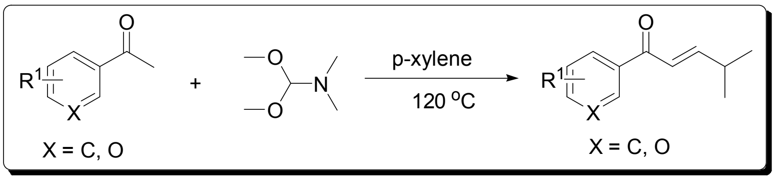

Dimethylformamide dimethylacetal (DMFDMA) (10 mmol, 1.19 g) and 1-Pyridin-3-yl-ethanone (10 mmol, 1.21 g) were dissolved in *p*-xylene (2 mL). And the mixture was refluxed during a period of 5 to 12 hours, during which time the formation of yellow precipitate. The precipitate was filtered out and washed with petroleum ether three times. The solid was vacuum-dried, and 1.65 g (yield 94 %) of a yellow solid was obtained **L1** 3-Dimethylamino-1-pyridin-3-yl-propenone. ^{1}H NMR (500 MHz, d^6-DMSO): δ 14.51 (s, 1 H), 7.92-7.90 (t, *J* = 7.5 Hz, 2 H), 7.37-7.34 (s, *J* = 7.8 Hz, 1 H), 6.83 (d, *J* = 2.0 Hz, 1 H), 5.98-5.95 (d, *J* = 12 Hz, 1 H), 3.19 (s, 3 H), 2.98 (s, 3 H); ^{13}C NMR (125 MHz, d^6-DMSO): δ 191.1, 163.6, 134.9, 129.9, 121.2, 119.1, 118.7, 90.4, 46.1, 38.6.

General procedure for preparation of 3 and 5

A mixture of benzenethiol **1a** (33.0 mg, 0.3 mmol), 5-phenylpenta-2,4-dienoic acid ethyl ester

2a (72.7 mg, 0.36 mmol), CuI (5.7 mg, 10 mol%), 3-dimethylamino-1-(2-hydroxypyridin-3-yl)-propenone **L4** (5.8 mg, 10 mol %) and Cs_2CO_3 (195.6 mg, 2 equiv) in DMSO (4 mL) was stirred under a N_2 atmosphere. After the reaction mixture was stirred at 60 °C for 24 h, it was allowed to cool to ambient temperature. Then the mixture was quenched with saturated salt water (10 mL), and the solution was extracted with ethyl acetate (3 × 10 mL). The organic layers were combined and dried by sodium sulfate and concentrated in vacuo. The pure product 5-phenyl-3-phenylsulfanylpenta-2,4-dienoic acid ethyl ester **3a** (75.3 mg, 81% yield) was obtained by flash column chromatography on silica gel.

Analytical Datas

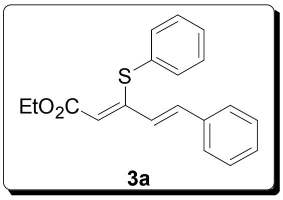

5-Phenyl-3-phenylsulfanylpenta-2,4-dienoic acid ethyl ester (3a)

75.3 mg, 81% yield; Yellow soild; Mp 111-113 ^{o}C;

^{1}H NMR (500 MHz, $CDCl_3$): δ 8.41 (dd, 1 H, J = 15.9 Hz, 0.8 Hz), 8.20 (d, 2 H, J = 8.9 Hz), 7.57 (d, 2 H, J = 8.9 Hz), 7.50-7.55 (m, 2 H), 7.30-7.43 (m, 4 H), 6.73 (m, 1 H), 5.90 (m, 1 H), 4.23 (q, 2 H), 1.35 (t, 3 H);

^{13}C NMR (125 MHz, $CDCl_3$): 160.8 (C), 145.9 (C), 142.8 (C), 140.7 (C), 136.9 (CH), 133.7 (CH), 129.0 (CH), 127.5 (CH), 126.8 (CH), 125.8 (CH), 122.4 (CH), 121.3 (CH), 120.1 (CH), 58.6 (CH_2), 11.3 (CH_3);

ESI-HRMS m/z: Calcd for $C_{19}H_{19}O_2S^+$ $[M+H]^+$: 311.1100; Found 311.0997.

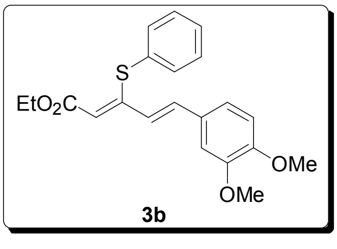

5-(4-Methoxy-3-methylphenyl)-3-phenylsulfanylpenta-2,4-dienoic acid ethyl ester (3b)

84.5 mg, 76% yield; Yellow solid; Mp 102-103 ^{o}C;

^{1}H NMR (500 MHz, $CDCl_3$): δ 8.25 (dd, 1 H, J = 16.0, 0.9 Hz), 7.49-7.56 (m, 2 H), 7.40-7.46 (m, 3

H), 7.34 (d, 1 H, J = 16.0 Hz), 7.10-7.16 (m, 2 H), 6.85 (d, 1 H, J = 12.7 Hz), 5.31 (s, 1 H), 4.11 (q, 2 H), 3.93 (s, 3 H), 3.91 (s, 3 H), 1.23 (t, 3 H);

^{13}C NMR (125 MHz, $CDCl_3$): δ 165.5 (C), 155.7 (C), 150.1 (C), 149.1 (C), 136.1 (CH), 135.1 (CH), 129.8 (CH), 129.7 (C), 129.4 (CH), 129.3 (C), 122.1 (CH), 121.6 (CH), 112.8 (CH), 111.1 (CH), 109.7 (CH), 59.9 (CH_2), 55.9 (CH_3), 55.9 (CH_3), 14.3 (CH_3);

ESI-HRMS m/z: Calcd for $C_{21}H_{23}O_4S^+$ $[M+H]^+$: 371.1312; Found 371.1309.

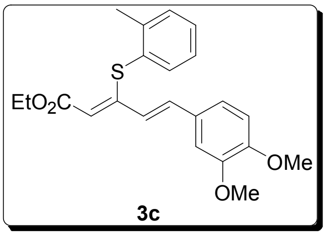

5-(3,4-Dimethoxyphenyl)-3-o-tolylsulfanylpenta-2,4-dienoic acid ethyl ester (3c)

98.0 mg, 85% yield; Yellow solid; Mp 78-82 °C;

^{1}H NMR (500 MHz, $CDCl_3$): δ 8.26 (dd, 1 H, J = 16.1, 0.8 Hz), 7.53 (d, 1 H, J = 7.2 Hz), 7.34-7.41 (m, 3 H), 7.25-7.29 (m, 1 H), 7.10-7.17 (m, 2 H), 6.86 (d, 1 H, J = 12.3 Hz), 5.05 (s, 1 H), 4.11 (q, 2 H), 3.94 (s, 3 H), 3.91 (s, 3 H), 2.44 (s, 3 H), 1.23 (t, 3 H);

^{13}C NMR (125 MHz, $CDCl_3$): δ 165.5 (C), 155.0 (C), 150.1 (C), 149.2 (C), 143.0 (C), 136.8 (CH), 135.8 (CH), 131.2 (CH), 130.3 (CH), 129.3 (C), 128.9 (C), 127.3 (CH), 122.2 (CH), 121.5 (CH), 111.1 (CH), 110.9 (CH), 109.7 (CH), 59.8 (CH_2), 56.0 (CH_3), 20.5 (CH_3), 14.4 (CH_3);

ESI-HRMS m/z: Calcd for $C_{22}H_{25}O_4S^+$ $[M+H]^+$: 385.1468; Found 385.1465.

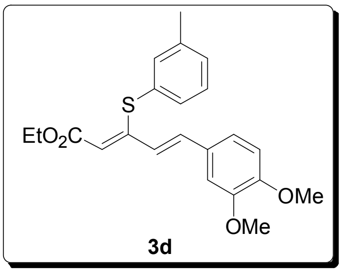

5-(3,4-Dimethoxyphenyl)-3-m-tolylsulfanylpenta-2,4-dienoic acid ethyl ester (3d)

94.6 mg, 82% yield; Yellow solid; Mp: 69-70 °C;

^{1}H NMR (500 MHz, $CDCl_3$): δ 8.23 (dd, 1 H, J = 16.1, 0.8 Hz), 7.29-7.39 (m, 4 H), 7.19-7.25 (m, 1 H), 7.07-7.16 (m, 2 H), 6.85 (d, 1 H, J = 12.2 Hz), 5.31 (s, 1 H), 4.12 (q, 2 H), 3.94 (s, 3 H), 3.91 (s, 3 H), 2.38 (s, 3 H), 1.24 (t, 3 H);

^{13}C NMR (125 MHz, $CDCl_3$): δ 165.5 (C), 156.0 (C), 150.1 (C), 149.2 (C), 139.7 (C), 136.0 (CH),

135.7 (CH), 132.2 (CH), 130.3 (CH), 129.8 (C), 129.6 (CH), 129.3 (C), 122.2 (CH), 121.6 (CH), 112.6 (CH), 111.1 (CH), 109.7 (CH), 59.9 (CH_2), 55.9 (2 CH_3), 21.3 (CH_3), 14.4 (CH_3);

ESI-HRMS *m/z*: Calcd for $C_{22}H_{25}O_4S^+$ $[M+H]^+$: 385.1468; Found 385.1465.

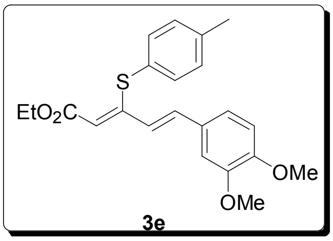

5-(3,4-Dimethoxyphenyl)-3-p-tolylsulfanylpenta-2,4-dienoic acid ethyl ester (3e)

95.7 mg, 83% yield; Yellow solid; Mp 109-112 ^{o}C;

^{1}H NMR (500 MHz, $CDCl_3$): δ 8.23 (dd, 1 H, *J* = 16.1, 0.7 Hz), 7.41 (m, 3 H), 7.23 (m, 2 H), 7.12 (m, 2 H), 6.85 (d, 1 H, *J* = 12.3 Hz), 5.25 (s, 1 H), 4.13 (q, 2 H), 3.93 (s, 3 H), 3.91 (s, 3 H), 2.40 (s, 3 H), 1.24 (t, 3 H);

^{13}C NMR (125 MHz, $CDCl_3$): δ 165.5 (C), 156.5 (C), 150.1 (C), 149.1 (C), 139.9 (C), 135.8 (CH), 135.4 (CH), 130.6 (CH), 129.3 (C), 126.3 (C), 122.2 (CH), 121.5 (CH), 111.9 (CH), 111.1 (CH), 109.7 (CH), 59.8 (CH_2), 55.9 (CH_3), 55.9 (CH_3), 21.4 (CH_3), 14.4 (CH_3);

ESI-HRMS *m/z*: Calcd for $C_{22}H_{25}O_4S^+$ $[M+H]^+$: 385.1468; Found 385.1465.

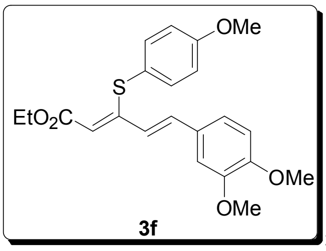

5-(3,4-Dimethoxyphenyl)-3-(4-methoxyphenylsulfanyl)penta-2,4-dienoic acid ethyl ester (3f)

106.9 mg, 89% yield; Yellow solid; Mp 127-130 ^{o}C;

^{1}H NMR (500 MHz, $CDCl_3$): δ 8.22 (dd, 1 H, *J* = 16.2, 0.9 Hz), 7.46 (d, 2 H, *J* = 8.9 Hz), 7.36 (d, 1 H, *J* = 16.2 Hz), 7.15-7.10 (m, 2 H), 6.97 (d, 2 H, *J* = 8.9 Hz), 6.85 (d, 1 H, *J* = 12.1 Hz), 5.17 (s, 1 H), 4.11 (q, 2 H), 3.94 (s, 3 H), 3.91 (s, 3 H), 3.86 (s, 3 H), 1.24 (t, 3 H);

^{13}C NMR (125 MHz, $CDCl_3$): δ 165.6 (C), 161.0 (C), 157.3 (C), 150.2 (C), 149.1 (C), 137.4 (CH), 135.5 (CH), 129.3 (C), 122.1 (CH), 121.4 (CH), 120.0 (C), 115.5 (CH), 111.3 (CH), 111.1 (CH), 109.8 (CH), 59.9 (CH_2), 56.1 (2 CH_3), 55.3 (CH_3), 14.3 (CH_3);

ESI-HRMS *m/z*: Calcd for $C_{22}H_{25}O_5S^+$ $[M+H]^+$: 401.1417; Found 401.1414.

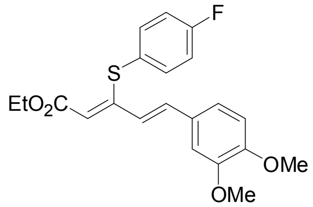

3g

5-(3,4-Dimethoxyphenyl)-3-(4-fluorophenylsulfanyl)penta-2,4-dienoic acid ethyl ester (3g)

90.9 mg, 78% yield; Yellow solid; Mp 113-115 ^{o}C;

^{1}H NMR (500 MHz, CDCl$_3$): δ 8.22 (dd, 1 H, *J* = 16.1, 0.8 Hz), 7.50-7.55 (m, 2 H), 7.34 (d, 1 H, *J* = 16.1 Hz), 7.08-7.17 (m, 4 H), 6.86 (d, 1 H, *J* = 12.3 Hz), 5.21 (s, 1 H), 4.12 (q, 2 H), 3.94 (s, 3 H), 3.91 (s, 3 H), 1.24 (t, 3 H);

^{13}C NMR (125 MHz, CDCl$_3$): δ 165.3 (C), 162.0 (C), 155.9 (C), 150.2 (C), 149.2 (C), 137.6 (CH), 136.1 (CH), 129.2 (C), 125.3 (C, d), 121.9 (CH), 121.3 (CH), 117.3 (CH), 112.3 (CH), 111.1 (CH), 109.7 (CH), 59.9 (CH$_2$), 55.9 (CH$_3$), 55.9 (CH$_3$), 14.3 (CH$_3$);

ESI-HRMS *m/z*: Calcd for $C_{21}H_{22}FO_4S^+$ $[M+H]^+$: 389.1217; Found 389.1214.

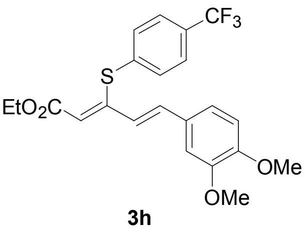

3h

5-(3,4-Dimethoxyphenyl)-3-(4-trifluoromethylphenylsulfanyl)penta-2,4-dienoic acid ethyl ester (3h)

101.3 mg, 77% yield; Yellow solid; Mp 77-78 ^{o}C;

^{1}H NMR (500 MHz, CDCl$_3$): δ 8.23 (dd, 1 H, *J* = 14.9, 0.4 Hz), 7.56-7.64 (m, 4 H), 7.33 (d, 1 H, *J* = 14.9 Hz), 7.06-7.12 (m, 2 H), 6.84 (d, 1 H, *J* = 12.2 Hz), 5.60 (s, 1 H), 4.16 (q, 2 H), 3.93 (s, 3 H), 3.90 (s, 3 H), 1.28 (t, 3 H);

^{13}C NMR (125 MHz, CDCl$_3$): δ 165.2 (C), 152.7 (C), 150.4 (C), 149.2 (C), 137.6 (CH), 135.0 (C), 133.1 (CH), 130.6 (C), 129.0 (C), 127.6 (C), 126.4 (CH), 121.8 (CH), 121.7 (CH), 116.5 (CH), 111.1 (CH), 109.7 (CH), 60.2 (CH$_2$), 55.9 (CH$_3$), 55.9 (CH$_3$), 14.3 (CH$_3$);

ESI-HRMS *m/z*: Calcd for $C_{22}H_{22}F_3O_4S^+$ $[M+H]^+$: 439.1185; Found 439.1182.

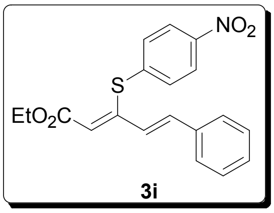

3-(4-Nitrophenylsulfanyl)-5-phenylpenta-2,4-dienoic acid ethyl ester (3i)

77.8 mg, 73% yield; Yellow solid; Mp 111-113 ^{o}C;

^{1}H NMR (500 MHz, CDCl$_3$): δ 8.35 (dd, 1 H, J = 15.9 Hz, 0.8 Hz), 8.18 (d, 2 H, J = 8.9 Hz), 7.54 (d, 2 H, J = 8.9 Hz), 7.47-7.52 (m, 2 H), 7.28-7.41 (m, 4 H), 5.95 (s, 1 H), 4.21 (q, 2 H), 1.30 (t, 3 H);

^{13}C NMR (125 MHz, CDCl$_3$): 164.8 (C), 149.9 (C), 146.8 (C), 142.7 (C), 138.9 (CH), 135.7 (C), 131.0 (CH), 129.5 (CH), 128.8 (CH), 127.8 (CH), 124.4 (CH), 123.3 (CH), 121.1 (CH), 60.6 (CH$_2$), 14.3 (CH$_3$);

ESI-HRMS *m/z*: Calcd for $C_{19}H_{18}NO_4S^+$ [M+H]$^+$: 356.0951; Found 356.0948.

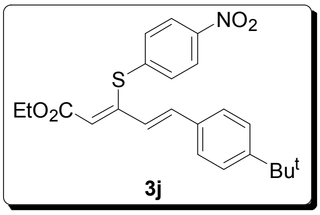

3-(4-Nitrophenylsulfanyl)-5-p-tolylpenta-2,4-dienoic acid ethyl ester (3j)

76.6 mg, 72% yield; Yellow solid; Mp 112-114 ^{o}C;

^{1}H NMR (500 MHz, CDCl$_3$): δ 8.32 (dd, 1 H, J = 15.8, 0.8 Hz), 8.17 (d, 2H, J = 9.0 Hz), 7.52 (d, 2 H, J = 9.0 Hz), 7.44 (d, 2 H, J = 8.4 Hz), 7.36 (d, 2 H, J = 8.4 Hz), 7.31 (d, 1 H, J = 15.8 Hz), 5.97 (s, 1 H), 4.22 (q, 2 H), 1.26-1.35 (m, 12 H);

^{13}C NMR (125 MHz, CDCl$_3$): δ 164.9 (C), 153.1 (C), 149.8 (C), 146.7 (C), 143.1 (C), 139.1 (CH), 133.0 (C), 130.8 (CH), 127.7 (CH), 125.8 (CH), 124.4 (CH), 122.5 (CH), 121.0 (CH), 60.6 (CH$_2$), 34.8 (C), 31.2 (CH$_3$), 14.3 (CH$_3$);

ESI-HRMS *m/z*: Calcd for $C_{23}H_{26}NO_4S^+$ [M+H]$^+$: 412.1577; Found 412.1574.

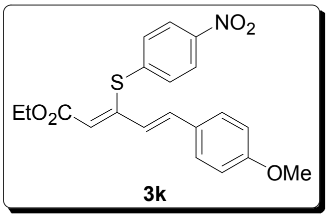

5-(4-Methoxyphenyl)-3-(4-nitrophenylsulfanyl)penta-2,4-dienoic acid ethyl ester (3k)

79.8 mg, 69% yield; Yellow solid; Mp80-82 ºC;

^{1}H NMR (500 MHz, $CDCl_3$): δ 8.26 (dd, 1 H, *J* = 15.8, 0.9 Hz), 8.16 (d, 2 H, *J* = 9.0 Hz), 7.52 (d, 2 H, *J* = 9.0 Hz), 7.46 (d, 2 H, *J* = 8.4 Hz), 7.30 (d, 1 H, *J* = 15.8 Hz), 6.87 (d, 2 H, *J* = 8.4 Hz), 5.92 (t, 1 H, *J* = 12.7 Hz), 4.21 (q, 2 H), 3.82 (s, 3 H), 1.30 (t, 3 H);

^{13}C NMR (125 MHz, $CDCl_3$): δ 165.0 (C), 160.88 (C), 150.0 (C), 146.7 (C), 143.2 (C), 138.8 (CH), 130.8 (CH), 129.4 (CH), 128.5 (C), 124.4 (CH), 121.2 (CH), 120.3 (CH), 114.3 (CH), 60.5 (CH_2), 55.4 (CH_3), 14.3 (CH_3);

ESI-HRMS *m/z*: Calcd for $C_{20}H_{20}NO_5S^+$ $[M+H]^+$: 386.1057; Found 386.1054.

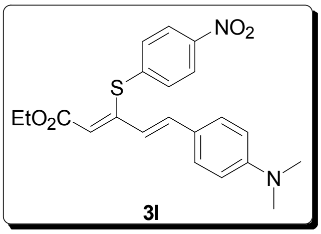

5-(4-Dimethylaminophenyl)-3-(4-nitrophenylsulfanyl)penta-2,4-dienoic acid ethyl ester (3l)

81.3 mg, 68% yield; Red solid; Mp 146-149 ºC;

^{1}H NMR (500 MHz, $CDCl_3$): δ 8.21 (dd, 1 H, *J* = 15.6, 0.7 Hz), 8.14 (d, 2 H, *J* = 9.0 Hz), 7.49 (d, 2 H, *J* = 9.0 Hz), 7.41 (d, 2 H, *J* = 8.8 Hz), 7.29 (d, 1 H, *J* = 15.6 Hz), 6.64 (d, 2 H, *J* = 8.8 Hz), 5.90 (s, 1 H), 4.22 (q, 2 H), 3.00 (s, 6 H), 1.30 (t, 3 H);

^{13}C NMR (125 MHz, $CDCl_3$): δ 165.3 (C), 151.4 (C), 150.1 (C), 146.4 (C), 144.2 (C), 140.1 (CH), 130.2 (CH), 129.6 (CH), 124.3 (CH), 123.6 (CH), 119.1 (C), 119.1 (CH), 118.7 (CH), 60.3 (CH_2), 40.2 (CH_3), 14.3 (CH_3);

ESI-HRMS *m/z*: Calcd for $C_{21}H_{23}N_2O_4S^+$ $[M+H]^+$: 399.1373; Found 399.1370.

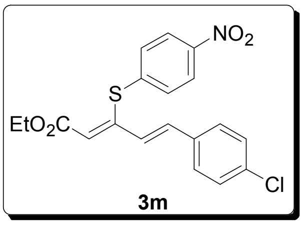

5-(4-Chlorophenyl)-3-(4-nitrophenylsulfanyl)penta-2,4-dienoic acid ethyl ester (3m)

87.7 mg, 75% yield; Yellow solid; Mp: 107-110 ºC;

^{1}H NMR (500 MHz, $CDCl_3$): δ 8.23 (dd, 1 H, *J* = 15.9, 0.9 Hz), 8.11 (d, 2 H, *J* = 9.0 Hz), 7.46 (d, 2 H, *J* = 9.0 Hz), 7.37 (d, 2 H, *J* = 8.4 Hz), 7.17-7.28 (m, 3 H), 5.87 (s, 1 H), 4.14 (q, 2 H), 1.23 (t, 3 H);

^{13}C NMR (125 MHz, $CDCl_3$): δ 164.8 (C), 149.7 (C), 147.0 (C), 142.1 (C), 137.3 (CH), 135.3 (C), 134.2 (C), 131.2 (CH), 129.2 (CH), 129.1 (CH), 124.5 (CH), 123.8 (CH), 121.2 (CH), 60.7 (CH_2), 14.3 (CH_3);

ESI-HRMS *m/z*: Calcd for $C_{19}H_{17}ClNO_4S^+$ $[M+H]^+$: 390.0561; Found 390.0558.

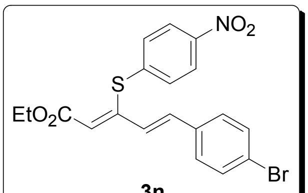

3n

5-(4-Bromophenyl)-3-(4-nitrophenylsulfanyl)penta-2,4-dienoic acid ethyl ester (3n)

93.8 mg, 72% yield; Yellow solid; Mp:117-120 °C;

^1H NMR (500 MHz, $CDCl_3$): δ 8.36 (dd, 1 H, J = 15.9, 0.8 Hz), 8.18 (d, 2 H, J = 9.0 Hz), 7.53 (d, 2 H, J = 9.0 Hz), 7.47 (d, 2 H, J = 8.5 Hz), 7.36 (d, 2 H, J = 8.5 Hz), 7.25 (d, 1 H, J = 15.9 Hz), 5.94 (s, 1 H), 4.20 (q, 2 H), 1.30 (t, 3 H);

^{13}C NMR (125 MHz, $CDCl_3$): δ 164.8 (C), 149.7 (C), 147.0 (C), 142.4 (C), 137.4 (CH), 134.6 (C), 132.0 (CH), 131.3 (CH), 129.2 (CH), 124.5 (CH), 123.9 (CH), 123.6 (C), 121.3 (CH), 60.7 (CH_2), 14.3 (CH_3);

ESI-HRMS *m/z*: Calcd for $C_{19}H_{17}BrNO_4S^+$ $[M+H]^+$: 434.0056; Found 434.0053.

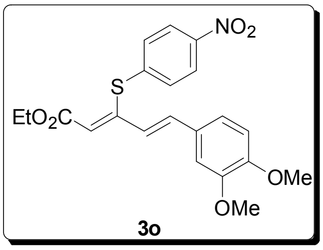

3o

5-(3,4-Dimethoxyphenyl)-3-(4-nitrophenylsulfanyl)penta-2,4-dienoic acid ethyl ester (3o)

60.7 mg, 73% yield; Yellow oil;

^1H NMR (500 MHz, $CDCl_3$): δ 8.25 (dd, 1 H, J = 15.8, 0.8 Hz), 8.17 (d, 2 H, J = 9.0 Hz), 7.52 (d, 2 H, J = 9.0 Hz), 7.29 (d, 1 H, J = 15.8 Hz), 7.06 (m, 2 H), 6.82 (m, 1 H), 5.92 (t, 1 H), 4.21 (q, 2 H), 3.92 (s, 3 H), 3.90 (s, 3 H), 1.30 (t, 3 H);

^{13}C NMR (125 MHz, $CDCl_3$): δ 165.0 (C), 150.6 (C), 149.9 (C), 149.2 (C), 146.7 (C), 143.2 (C), 139.1 (CH), 130.7 (CH), 128.8 (C), 124.4 (CH), 122.1 (CH), 121.4 (CH), 120.4 (CH), 111.1 (CH), 109.7 (CH), 60.5 (CH_2), 55.9 (CH_3), 55.9 (CH_3), 14.2 (CH_3);

ESI-HRMS *m/z*: Calcd for $C_{21}H_{22}NO_6S^+$ $[M+H]^+$: 416.1162; Found 416.1159.

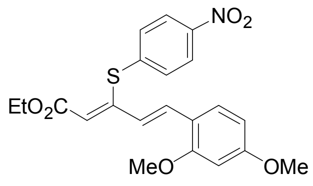

3p

5-(3,4-Dimethoxyphenyl)-3-(4-nitrophenylsulfanyl)penta-2,4-dienoic acid ethyl ester (3p)

87.3 mg, 70% yield; Yellow solid; Mp: 126-128 ^{o}C;

^{1}H NMR (500 MHz, $CDCl_3$): δ 8.28 (dd, 1 H, J = 16.0, 0.8 Hz), 8.15 (d, 2 H, J = 9.0 Hz), 7.67 (d, 1 H, J = 16.0 Hz), 7.52 (d, 3 H, J = 9.0 Hz), 6.38-6.51 (m, 2 H), 5.89 (s, 1 H), 4.21 (q, 2 H), 3.82 (s, 3 H), 3.81 (s, 3 H), 1.29 (t, 3 H);

^{13}C NMR (125 MHz, $CDCl_3$): δ 165.1 (C), 162.3 (C), 159.2 (C), 151.1 (C), 146.7 (C), 143.5 (C), 134.2 (CH), 131.0 (CH), 129.0 (CH), 124.5 (CH), 121.2 (CH), 119.3 (CH), 117.9 (C), 105.4 (CH), 98.4 (CH), 60.3 (CH_2), 55.6 (CH_3), 55.5 (CH_3), 14.3 (CH_3);

ESI-HRMS *m/z*: Calcd for $C_{21}H_{22}NO_6S^+$ $[M+H]^+$: 416.1162; Found 416.1159.

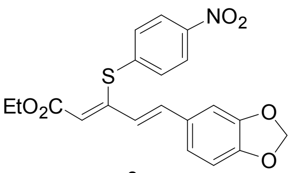

3q

5-Benzo[1,3]dioxol-5-yl-3-(4-nitrophenylsulfanyl)penta-2,4-dienoic acid ethyl ester (3q)

93.5 mg, 78% yield; Yellow solid; Mp 122-125 ^{o}C;

^{1}H NMR (500 MHz, $CDCl_3$): δ 8.13-8.26 (m, 3 H), 7.52 (d, 2 H, J = 8.9 Hz), 7.24 (d, 1 H, J = 15.8 Hz), 7.07 (d, 1 H, J = 1.5 Hz), 6.94 (dd, 1 H, J = 8.1, 1.6 Hz), 6.76 (d, 1 H, J = 12.0 Hz), 5.98 (s, 2 H), 5.92 (s, 1 H), 4.21 (q, 2 H), 1.30 (t, 3 H);

^{13}C NMR (125 MHz, $CDCl_3$): δ 165.0 (C), 149.8 (C), 149.1 (C), 148.4 (C), 146.8 (C), 143.1 (C), 138.8 (CH), 130.8 (CH), 130.2 (C), 124.4 (CH), 123.8 (CH), 121.6 (CH), 120.5 (CH), 108.5 (CH), 106.5 (CH), 101.5 (CH_2), 60.5 (CH_2), 14.3 (CH_3);

ESI-HRMS *m/z*: Calcd for $C_{20}H_{18}NO_6S^+$ $[M+H]^+$: 400.0849; Found 400.0846.

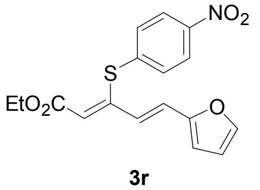

3r

5-Furan-2-yl-3-(4-nitrophenylsulfanyl)penta-2,4-dienoic acid ethyl ester (3r)

83.9 mg, 81% yield; Yellow oil;

^{1}H NMR (500 MHz, $CDCl_3$): δ 8.21 (d, 1 H, *J* = 16.1 Hz), 8.16 (d, 2 H, *J* = 9.0 Hz), 7.49 (d, 2 H, *J* = 9.0 Hz), 7.45 (d, 1 H, *J* = 1.7 Hz), 7.11 (d, 1 H, *J* = 16.1 Hz), 6.48 (d, 1 H, *J* = 12.4 Hz), 6.42 (dd, 1 H, *J* = 3.4, 1.7 Hz), 6.00 (s, 1 H), 4.23 (q, 2 H), 1.30 (t, 3 H);

^{13}C NMR (125 MHz, $CDCl_3$): δ 164.8 (C), 152.0 (C), 148.5 (C), 146.6 (C), 144.4 (CH), 143.3 (C), 130.2 (CH), 125.9 (CH), 124.4 (CH), 122.1 (CH), 121.6 (CH), 113.3 (CH), 112.3 (CH), 60.6 (CH_2), 14.3 (CH_3);

ESI-HRMS *m/z*: Calcd for $C_{17}H_{16}NO_5S^+$ $[M+H]^+$: 346.0744; Found 346.0741.

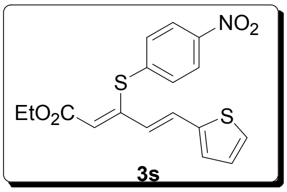

3s

3-(4-Nitrophenylsulfanyl)-5-thiophen-2-yl-penta-2,4-dienoic acid ethyl ester (3s)

91.1 mg, 84% yield; Yellow oil;

^{1}H NMR (500 MHz, $CDCl_3$): δ 8.17 (d, 1 H, *J* = 15.5 Hz), 8.16 (d, 2 H, *J* = 9.0 Hz), 7.50 (d, 2 H, *J* = 9.0 Hz), 7.46 (d, 1 H, *J* = 15.5 Hz), 7.32 (dd, 1 H, *J* = 5.0, 0.8 Hz), 7.12 (dd, 1 H, *J* = 3.6, 0.8 Hz), 7.00 (dd, 1 H, *J* = 5.0, 3.6 Hz), 5.97 (s, 1 H), 4.22 (q, 2 H), 1.31 (t, 3 H);

^{13}C NMR (125 MHz, $CDCl_3$): δ 164.8 (C), 148.7 (C), 146.7 (C), 143.1 (C), 141.3 (C), 131.9 (CH), 130.5 (CH), 129.6 (CH), 128.1 (CH), 127.8 (CH), 124.4 (CH), 122.8 (CH), 121.6 (CH), 60.6 (CH_2), 14.3 (CH_3);

ESI-HRMS *m/z*: Calcd for $C_{17}H_{16}NO_4S_2^+$ $[M+H]^+$: 362.0515; Found 362.0512.

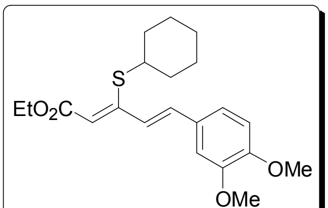

5a

3-Cyclohexylsulfanyl-5-(3,4-dimethoxyphenyl)penta-2,4-dienoic acid ethyl ester (5a)

99.4 mg, 88% yield; Yellow oil;

^{1}H NMR (500 MHz, $CDCl_3$): δ 8.19 (dd, 1 H, *J* = 16.1, 0.8 Hz), 7.25 (d, 1 H, *J* = 16.1 Hz), 7.05-7.13 (m, 2 H), 6.84 (d, 1 H, *J* = 12.3 Hz), 5.71 (s, 1 H), 4.19 (q, 2 H), 3.92 (s, 3 H), 3.90 (s, 3 H), 3.14-

3.25 (m, 1 H), 2.02-2.14 (m, 2 H), 1.76-1.86 (m, 2 H), 1.60-1.70 (m, 1 H), 1.37-1.54 (m, 5 H), 1.26-1.36 (m, 3 H);

^{13}C NMR (125 MHz, $CDCl_3$): δ 165.5 (C), 154.0 (C), 150.0 (C), 149.1 (C), 136.1 (CH), 129.5 (C), 123.1 (CH), 121.4 (CH), 111.4 (CH), 111.1 (CH), 109.7 (CH), 59.9 (CH_2), 55.94 (CH_3), 55.91 (CH_3), 44.0 (CH), 32.7 (CH_2), 26.0 (CH_2) 25.9 (CH_2), 14.5 (CH_3);

ESI-HRMS *m/z*: Calcd for $C_{21}H_{29}O_4S^+$ $[M+H]^+$: 377.1781; Found 377.1778.

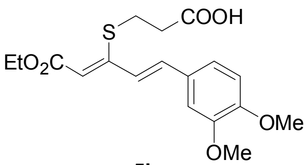

5b

3-(2-Carboxyethylsulfanyl)-5-(3,4-dimethoxyphenyl)penta-2,4-dienoic acid ethyl ester (5b) 89.0 mg, 81% yield; Yellow oil;

^{1}H NMR (500 MHz, $CDCl_3$): δ 8.21 (d, 1 H, J = 16.1 Hz), 7.39-7.41 (m, 3 H), 7.06-7.16 (m, 2 H), 6.91 (d, 2 H, J = 8.6 Hz), 6.85 (d, 1 H, J = 12.2 Hz), 5.17 (s, 1 H), 4.12 (q, 2 H), 3.93 (s, 3 H), 3.91 (s, 3 H), 1.24 (t, 3 H);

^{13}C NMR (125 MHz, $CDCl_3$): δ 165.7 (C), 157.4 (C), 157.3 (C), 156.1 (C), 150.1 (C), 149.2 (C), 137.7 (CH), 135.6 (CH), 132.9 (CH), 129.3 (C), 121.6 (CH), 117.0 (CH), 116.1 (CH), 111.2 (CH), 109.7 (CH), 59.9 (CH_2), 55.97 (CH_3), 55.94 (CH_3), 14.4 (CH_3);

ESI-HRMS *m/z*: Calcd for $C_{18}H_{23}O_6S^+$ $[M+H]^+$: 367.1210; Found 367.1207.

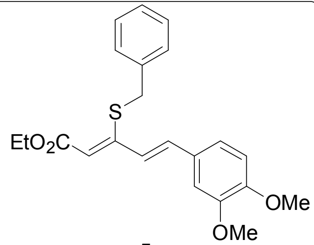

5c

3-Benzylsulfanyl-5-(3,4-dimethoxyphenyl)penta-2,4-dienoic acid ethyl ester (5c)

87.7 mg, 76% yield; Yellow solid; Mp: 99-102 ^{o}C;

^{1}H NMR (500 MHz, $CDCl_3$): δ 8.19 (dd, 1 H, J = 16.2, 0.9 Hz), 7.28-7.41 (m, 5 H), 7.21 (d, 1 H, J = 16.2 Hz), 7.05-7.10 (m, 2 H), 6.83 (d, 1 H, J = 12.3 Hz), 5.69 (s, 1 H), 4.19 (q, 2 Hz), 4.09 (s, 2 H), 3.92 (s, 3 H), 3.90 (s, 3 H), 1.31 (t, 3 H);

^{13}C NMR (125 MHz, $CDCl_3$): δ 165.2 (C), 155.1 (C), 150.1 (C), 149.1 (C), 135.9 (C), 135.2 (CH),

129.3 (C), 129.1 (CH), 128.8 (CH), 127.7 (CH), 122.3 (CH), 121.5 (CH), 111.1 (CH), 110.4 (CH), 109.7 (CH), 59.9 (CH_2), 55.94 (CH_3), 55.91 (CH_3), 36.8 (CH_2), 14.4 (CH_3);

ESI-HRMS *m/z*: Calcd for $C_{22}H_{25}O_4S^+$ $[M+H]^+$: 385.1468; Found 385.1465.

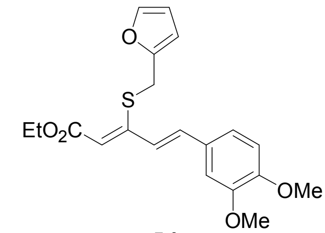

5d

5-(3,4-Dimethoxyphenyl)-3-(furan-2-ylmethylsulfanyl)penta-2,4-dienoic acid ethyl ester (5d)

95.5 mg, 85% yield; Yellow oil;

^{1}H NMR (500 MHz, $CDCl_3$): δ 8.18 (1 H, dd, *J* = 16.2, 0.7 Hz), 7.38 (dd, 1 H, *J* = 1.8, 0.8 Hz), 7.21 (d, 1 H, *J* = 16.2 Hz), 7.04-7.12 (m, 2 H), 6.84 (d, 1 H, *J* = 12.3 Hz), 6.27-6.3(d, 1 H, *J* = 7.1 Hz);

^{13}C NMR (125 MHz, $CDCl_3$): δ 165.2 (C), 154.0 (C), 150.1 (C), 149.1 (C), 149.0 (C), 142.6 (CH), 136.3 (CH), 129.2 (C), 122.3 (CH), 121.5 (CH), 111.3 (CH), 111.1 (CH), 110.7 (CH), 109.7 (CH), 108.6 (CH), 60.1 (CH_2), 55.94 (CH_3), 55.91 (CH_3), 29.2 (CH_2), 14.4 (CH_3);

ESI-HRMS *m/z*: Calcd for $C_{20}H_{23}O_5S^+$ $[M+H]^+$: 375.1261; Found 375.1258.

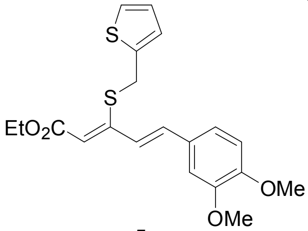

5e

5-(3,4-Dimethoxyphenyl)-3-(thiophen-2-ylmethylsulfanyl)penta-2,4-dienoic acid ethyl ester (5e)

97.2 mg, 83% yield; Yellow oil;

^{1}H NMR (500 MHz, $CDCl_3$): δ 8.23 (1 H, dd, *J* = 16.2, 0.7 Hz), 7.43 (dd, 1 H, *J* = 1.8, 0.8 Hz), 7.16 (d, 1 H, *J* = 16.2 Hz), 7.00-7.10 (m, 2 H), 6.81 (d, 1 H, *J* = 12.3 Hz), 6.25-6.0 (d, 1 H, *J* = 7.1 Hz);

^{13}C NMR (125 MHz, $CDCl_3$): δ 165.2 (C), 154.0 (C), 150.1 (C), 149.1 (C), 149.0 (C), 142.6 (CH), 136.3 (CH), 129.2 (C), 122.3 (CH), 121.5 (CH), 111.3 (CH), 111.1 (CH), 110.7 (CH), 109.7 (CH), 108.6 (CH), 60.1 (CH_2), 55.94 (CH_3), 55.91 (CH_3), 29.2 (CH_2), 14.4 (CH_3);

ESI-HRMS *m/z*: Calcd for $C_{20}H_{23}O_4S_2^+$ $[M+H]^+$: 391.1038; Found 391.1035.

Spectrums

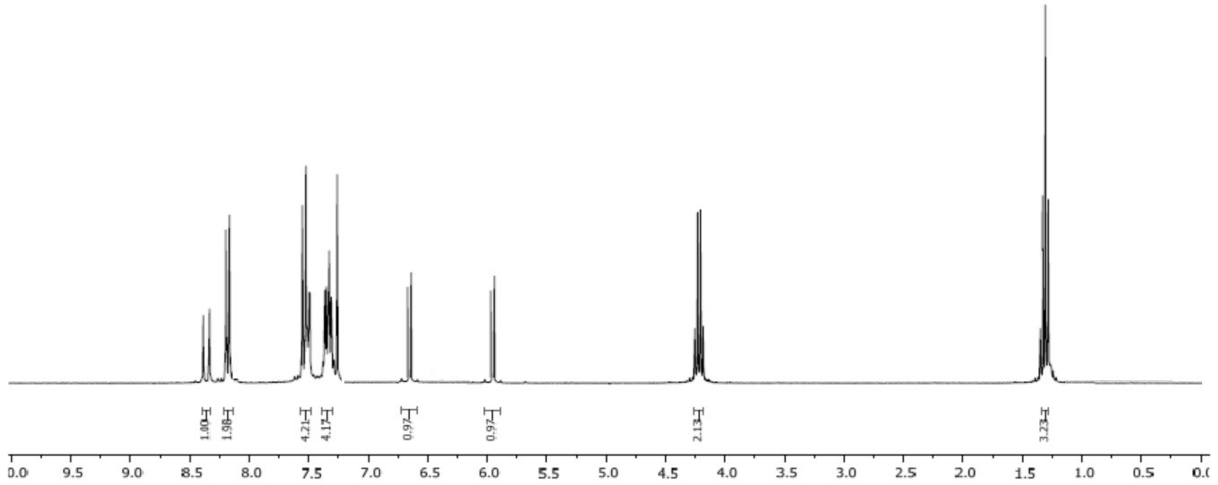

Fig 1. 3a ^{1}H NMR

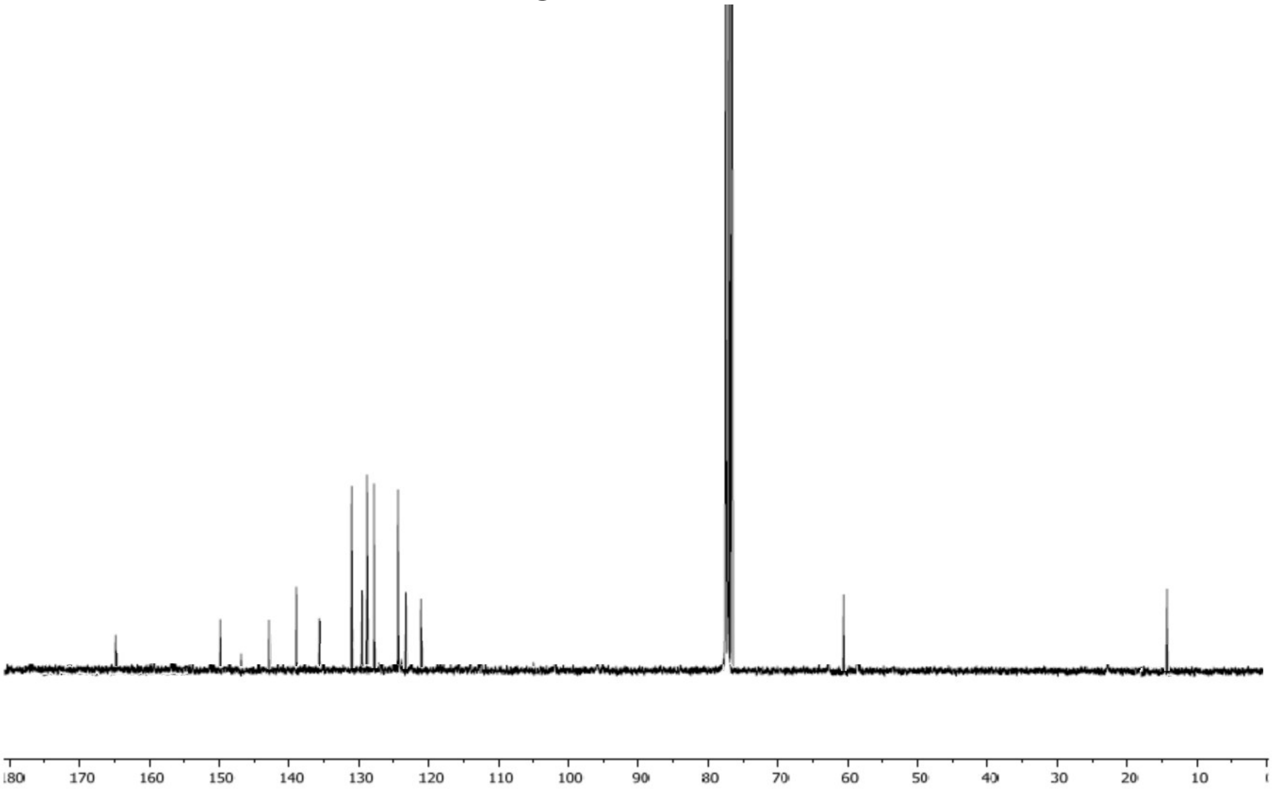

Fig 2. 3a ^{13}C NMR

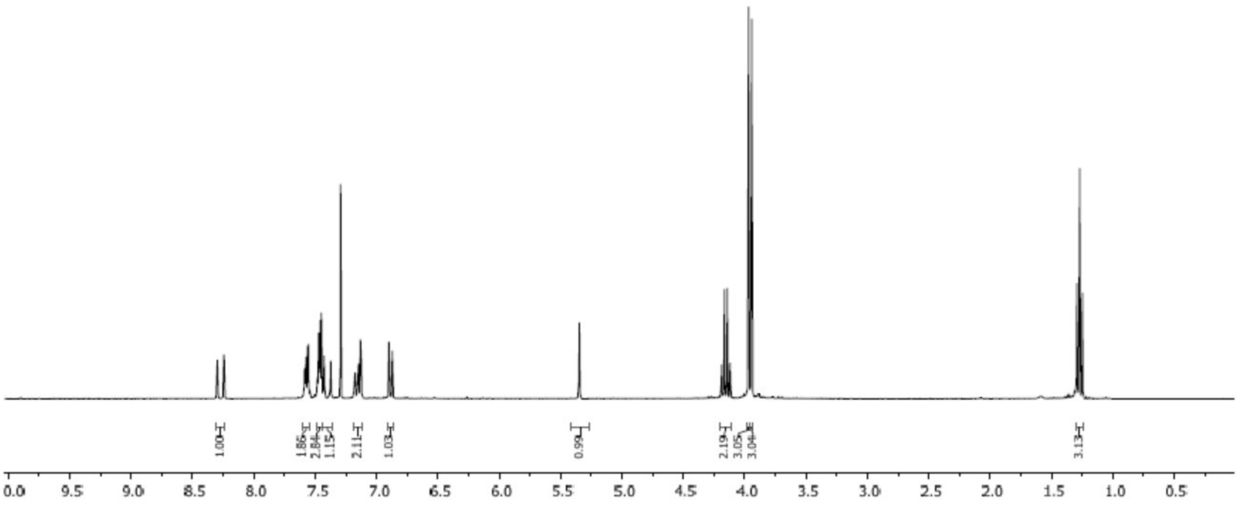

Fig 3. 3b ^{1}H NMR

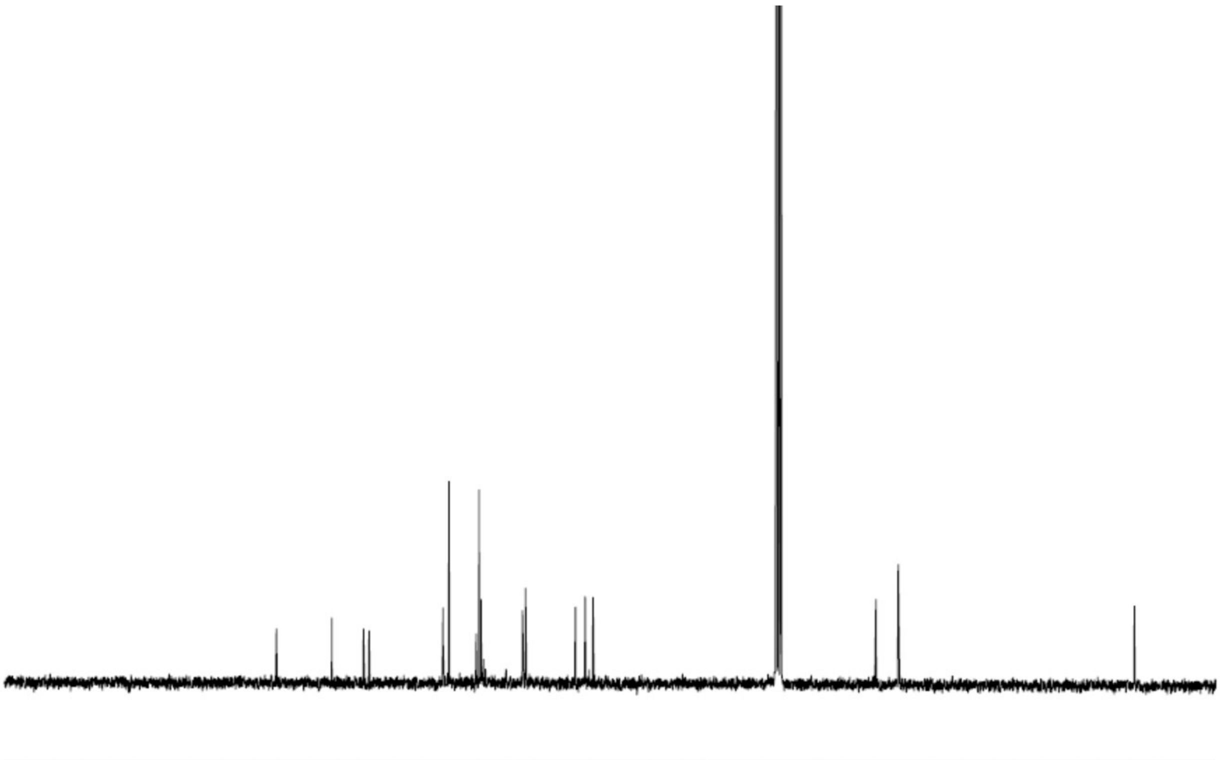

Fig 4. 3b ^{13}C NMR

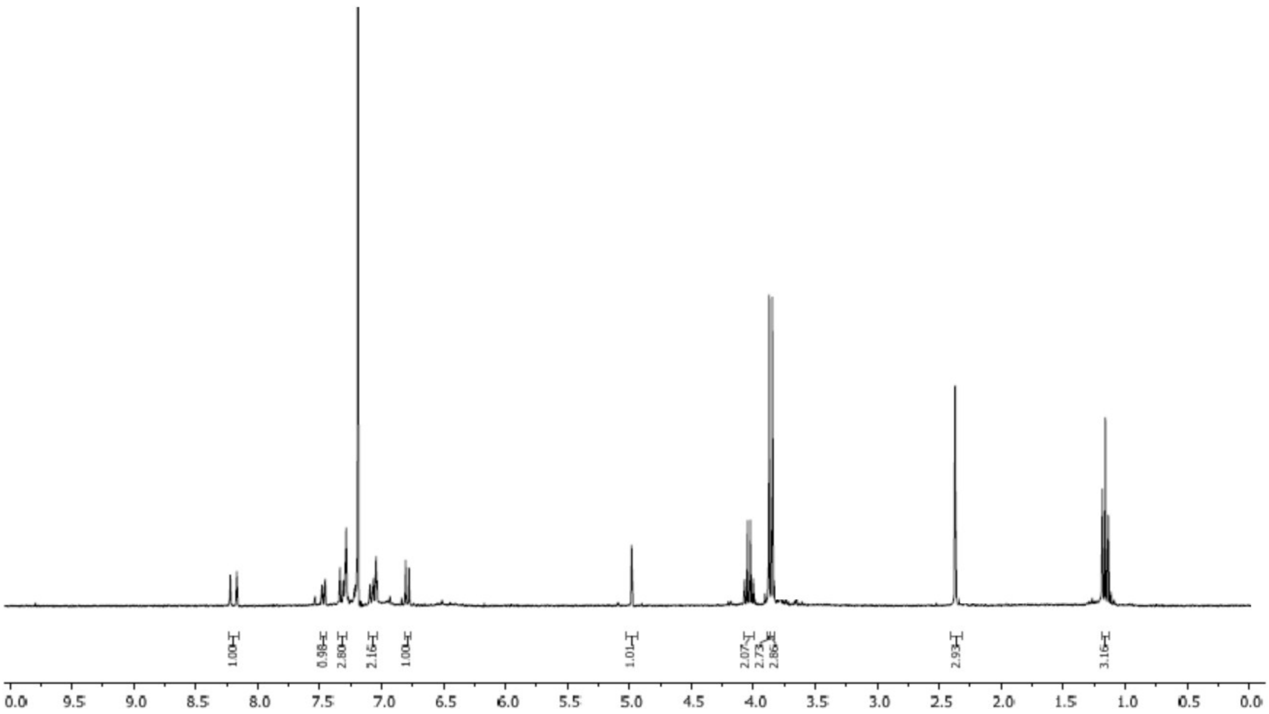

Fig 5. 3c ^{1}H NMR

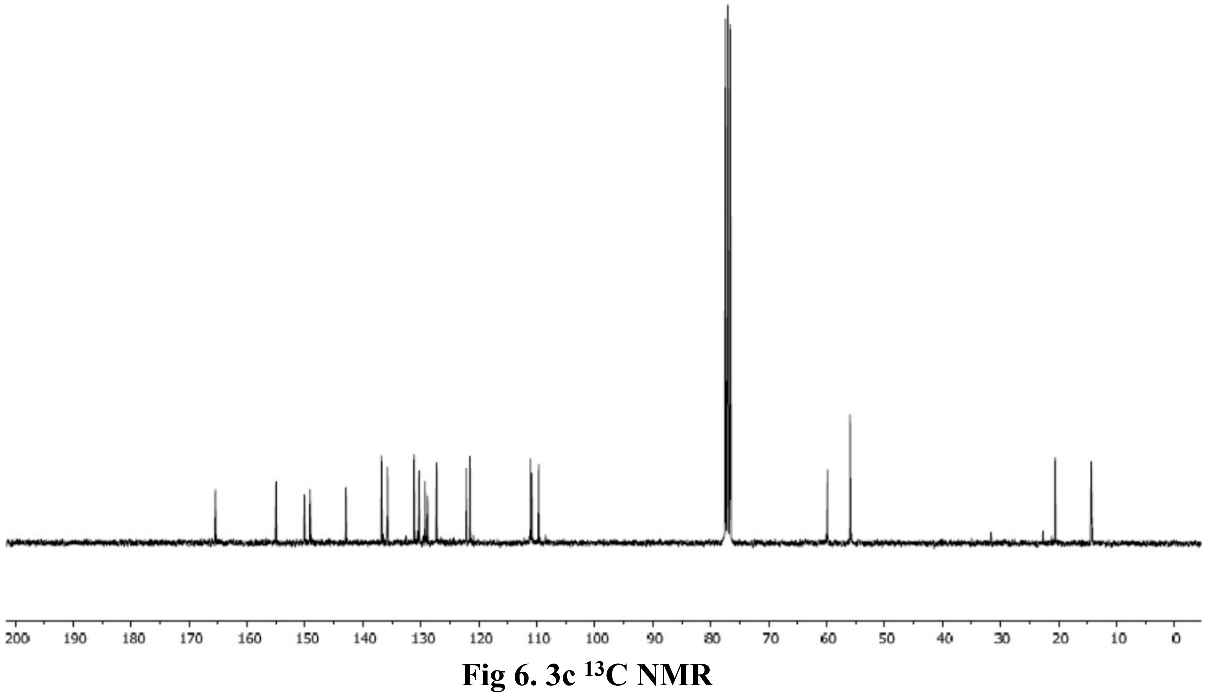

Fig 6. 3c ^{13}C NMR

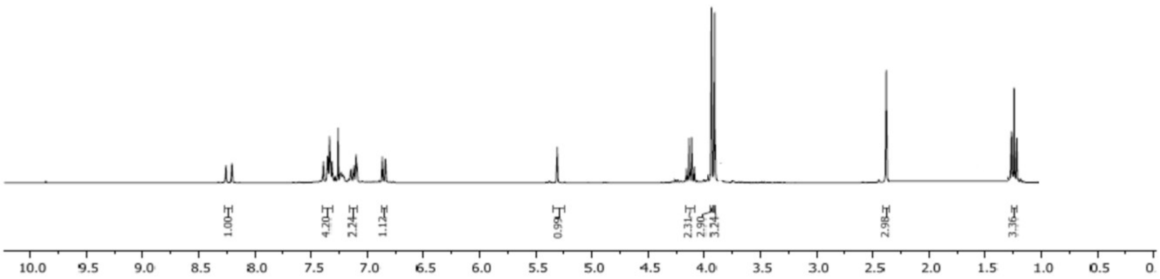

Fig 7. 3d ^{1}H NMR

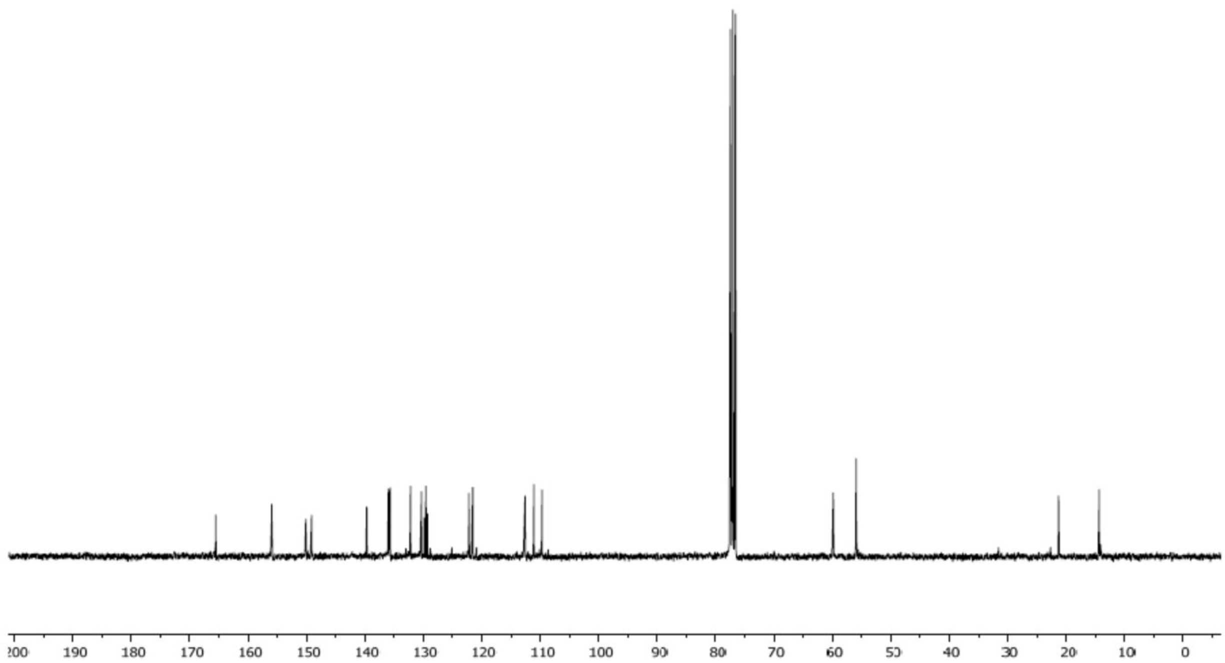

Fig 8. 3d ^{13}C NMR

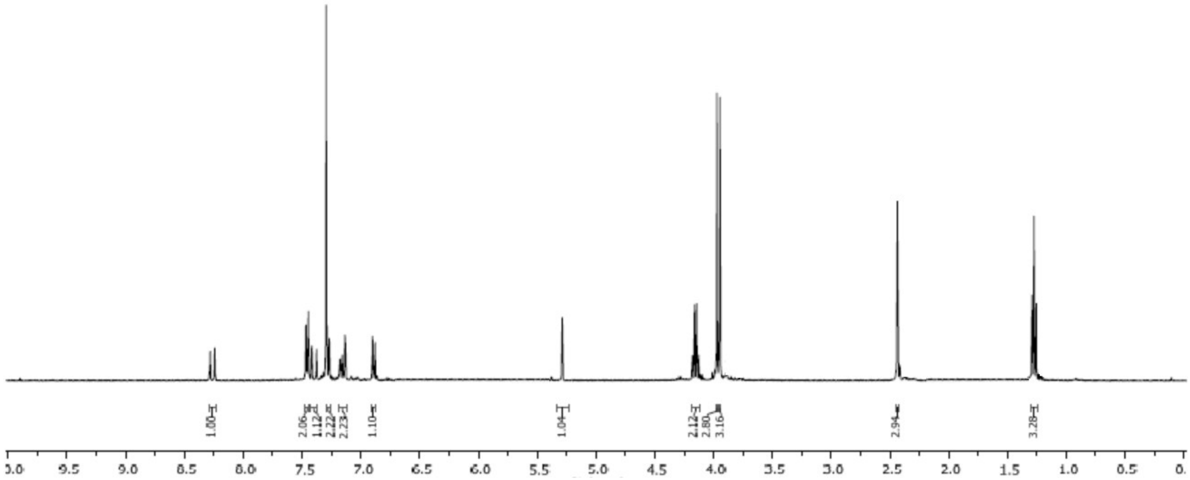

Fig 9. 3e ¹H NMR

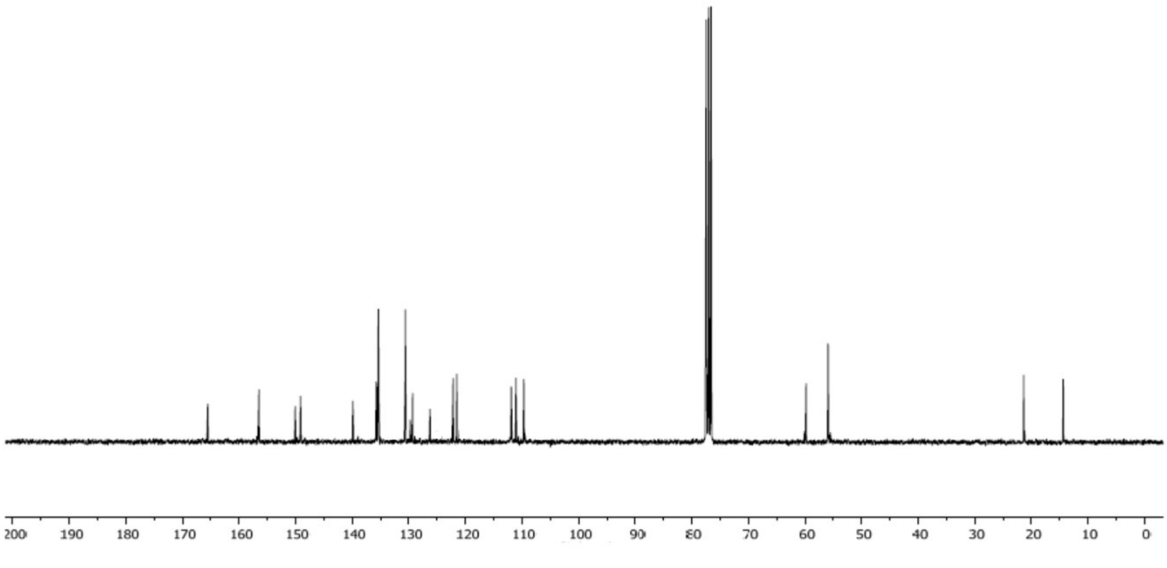

Fig 10. 3e ¹³C NMR

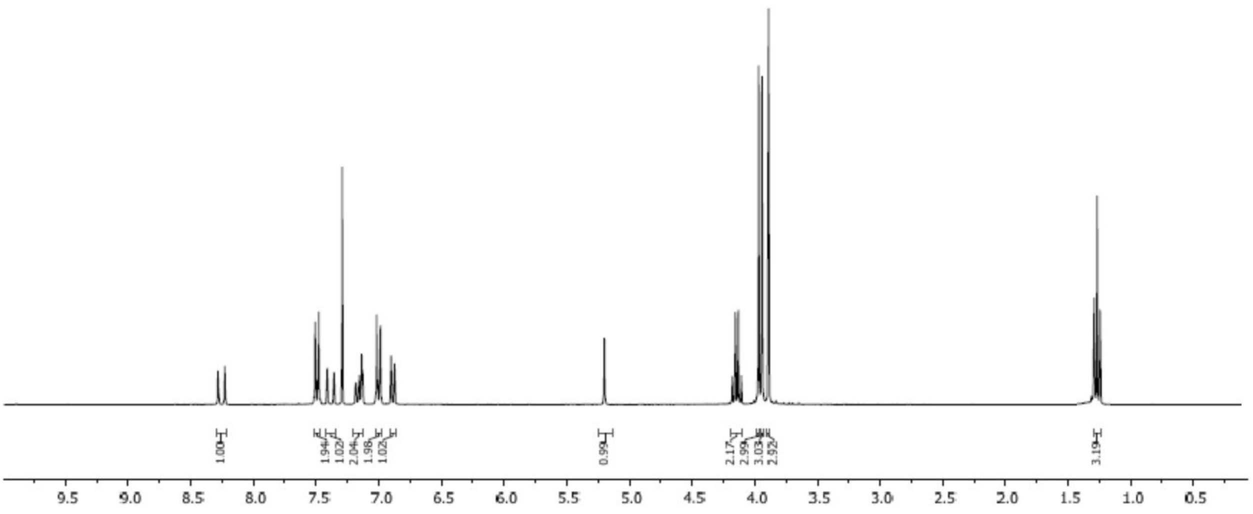

Fig 11. 3f ^{1}H NMR

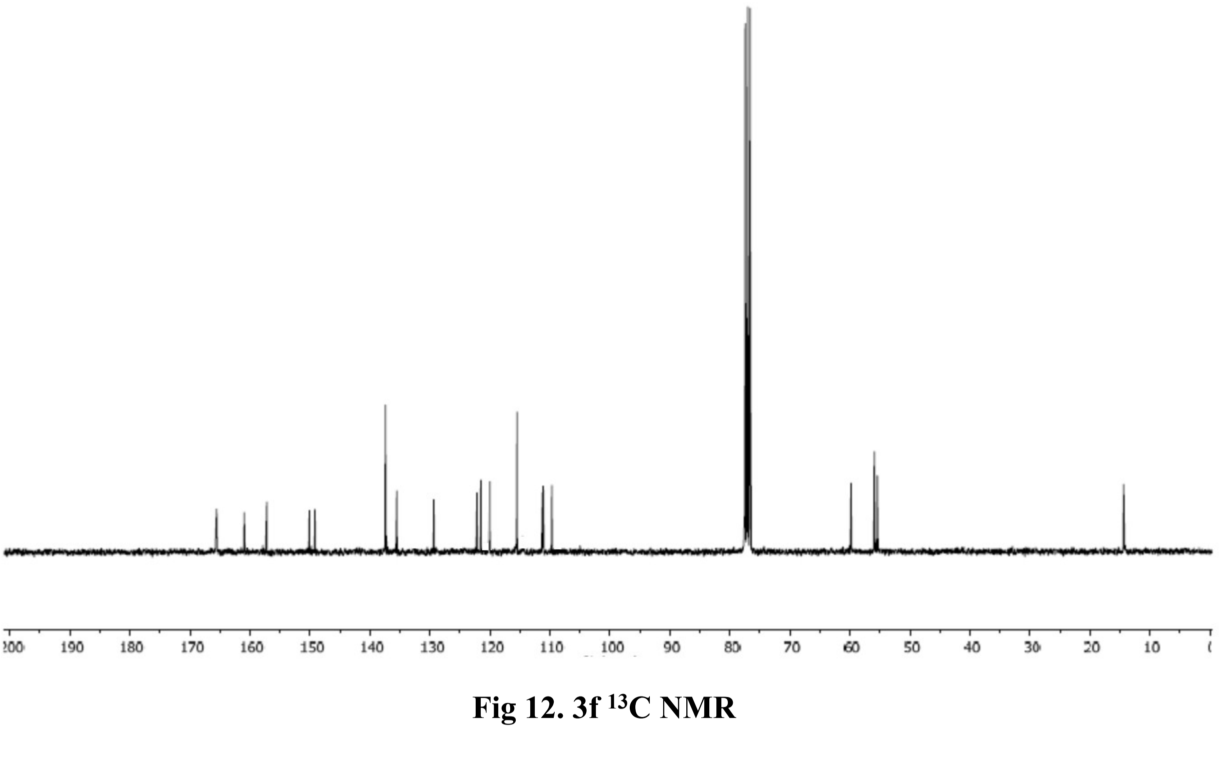

Fig 12. 3f ^{13}C NMR

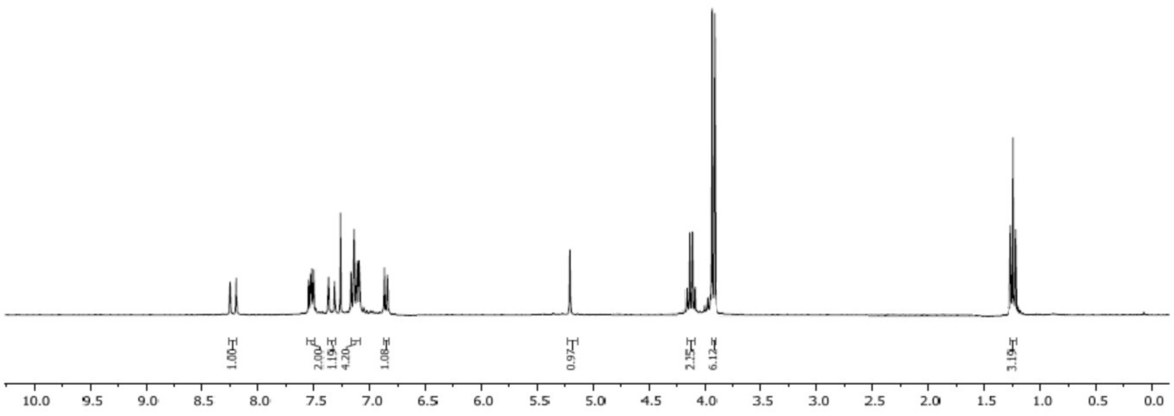

Fig 13. 3g ^{1}H NMR

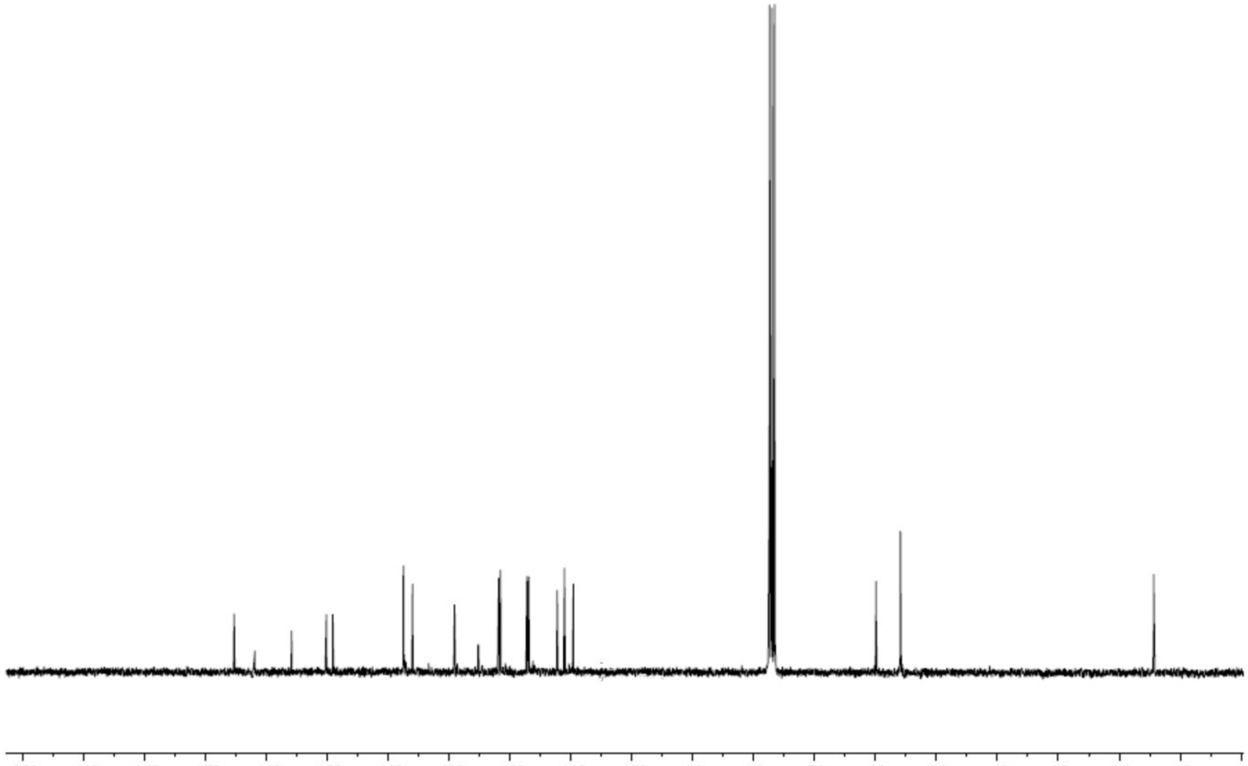

Fig 14. 3g ^{13}C NMR

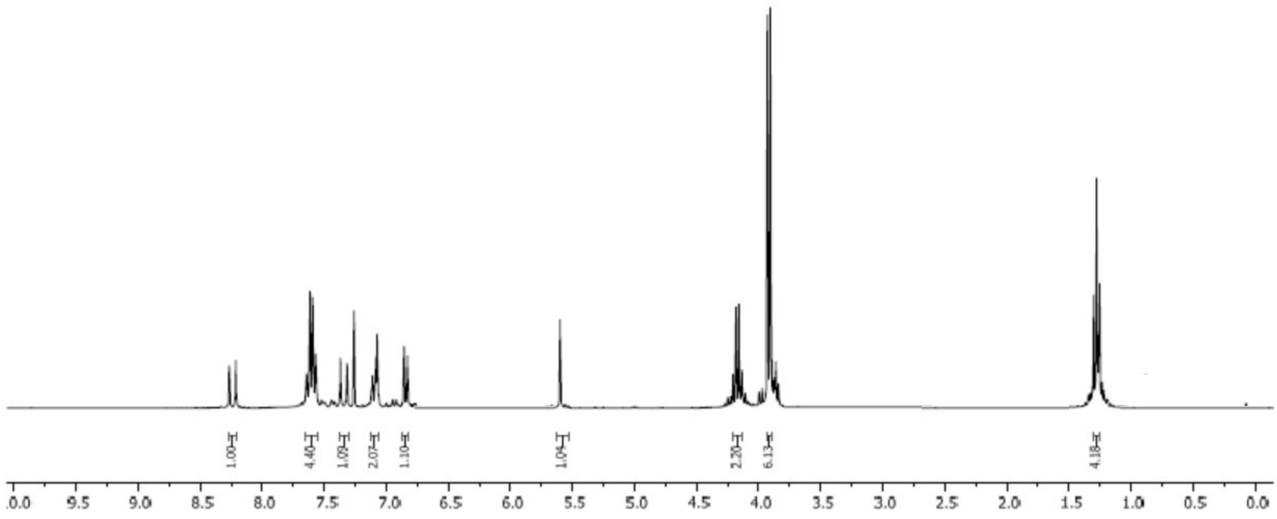

Fig 15. 3h ^{1}H NMR

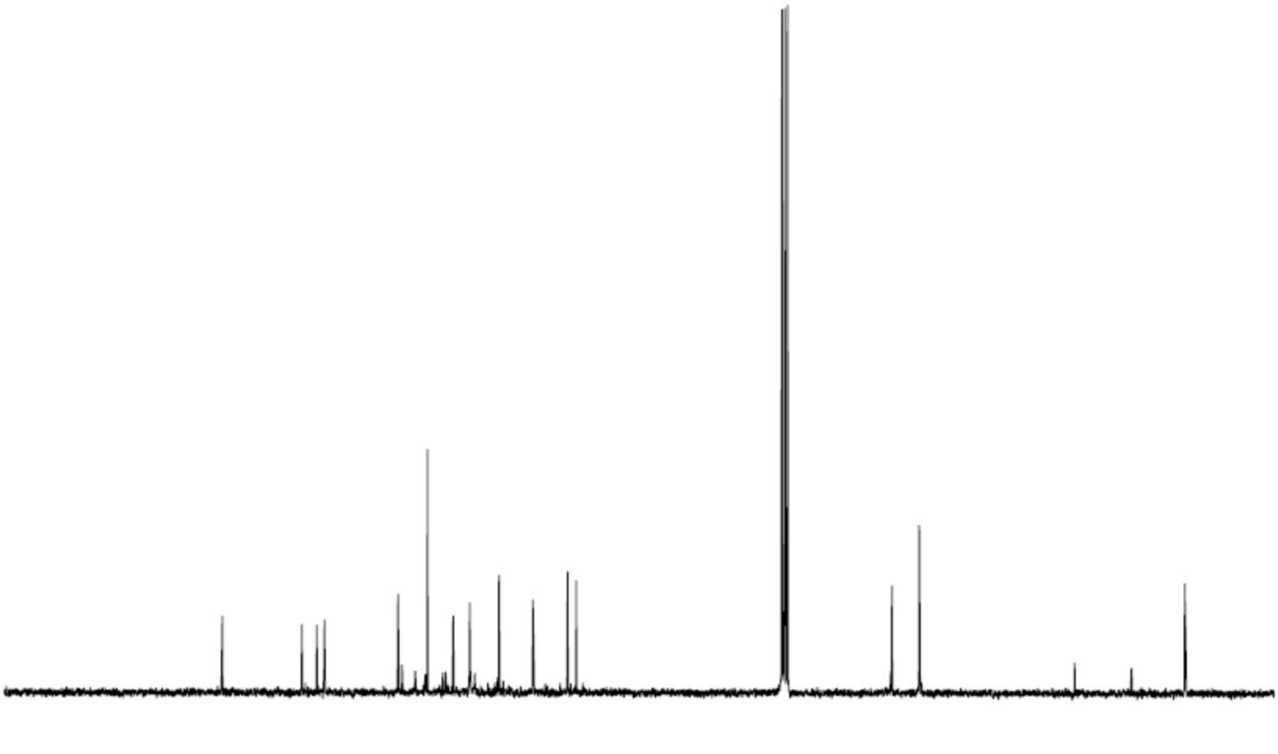

Fig 16. 3h ^{13}C NMR

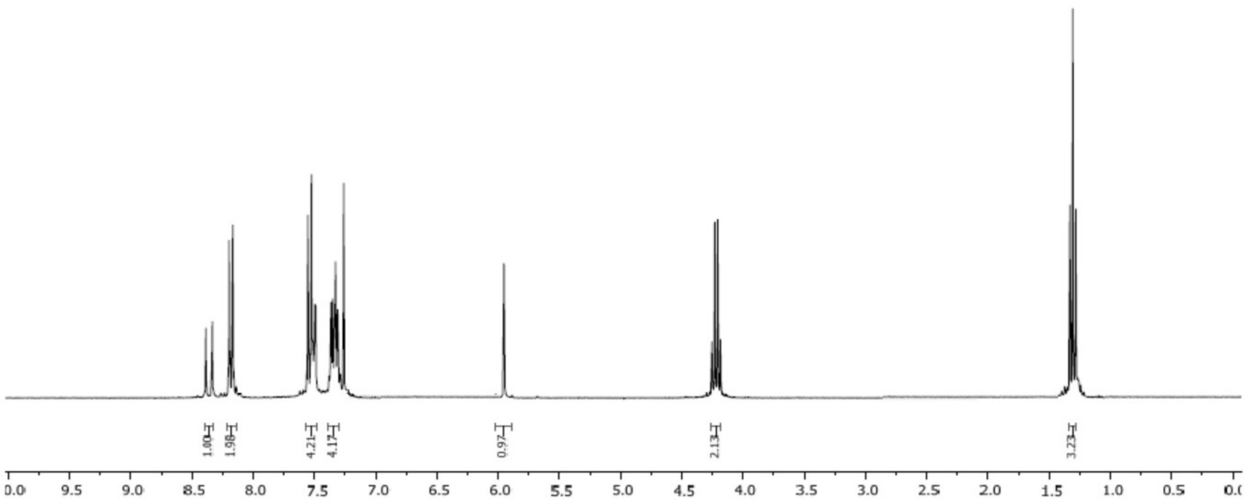

Fig 17. 3i ^{1}H NMR

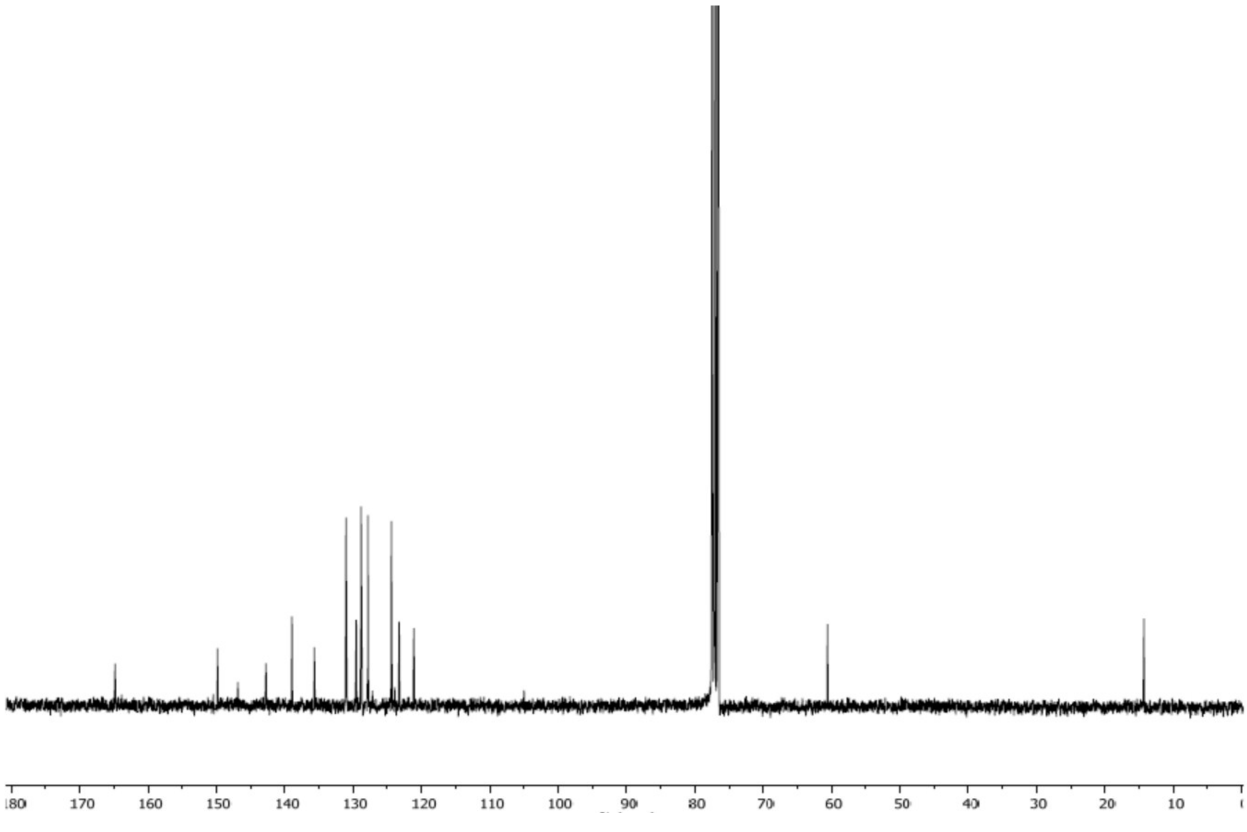

Fig 18. 3i ^{13}C NMR

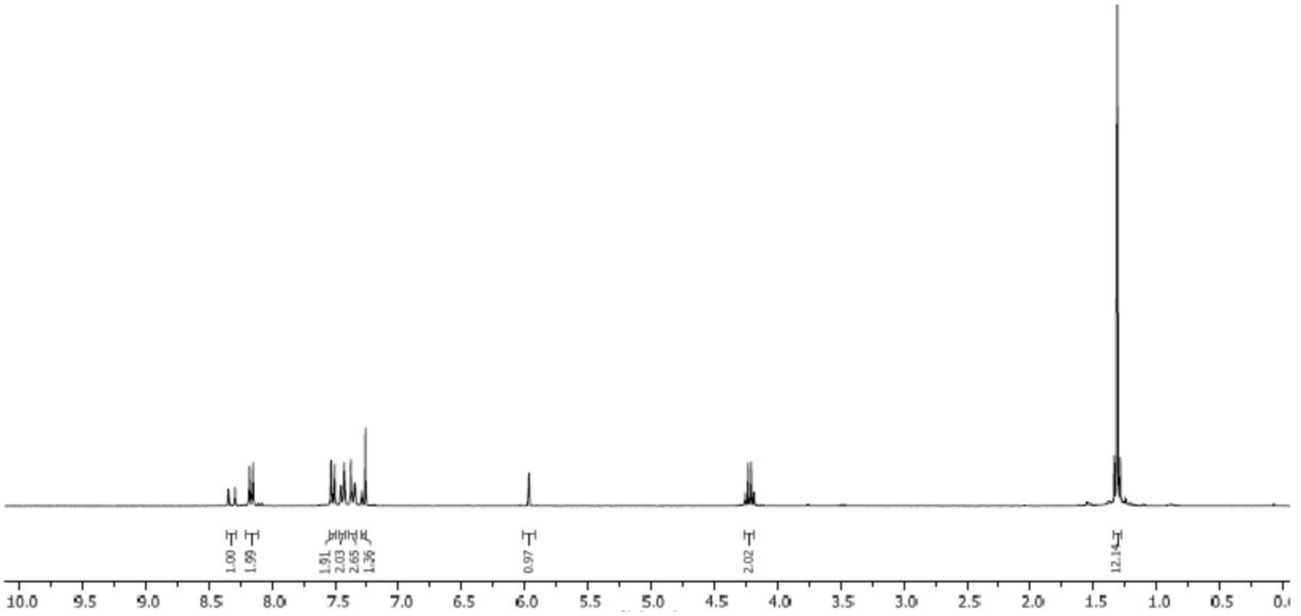

Fig 19. 3j ^{1}H NMR

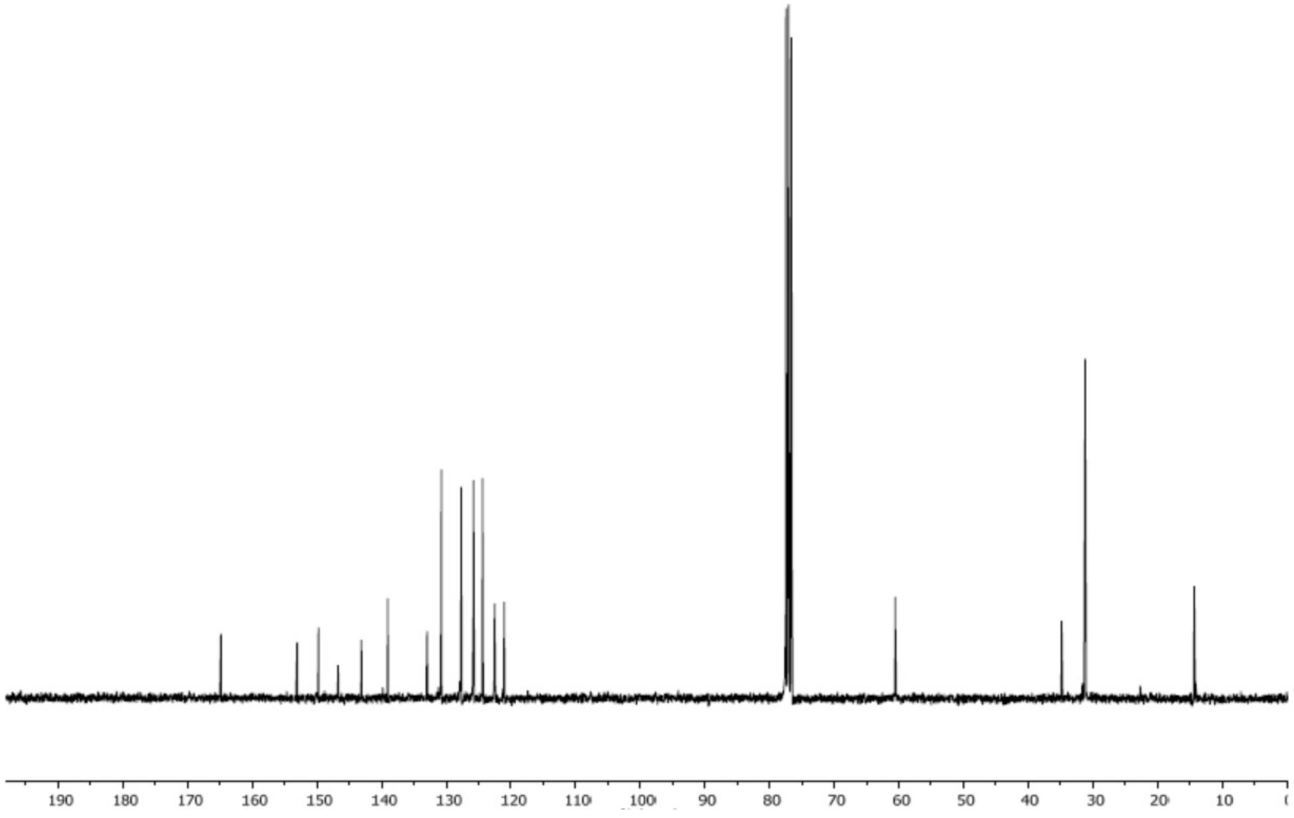

Fig 20. 3j ^{13}C NMR

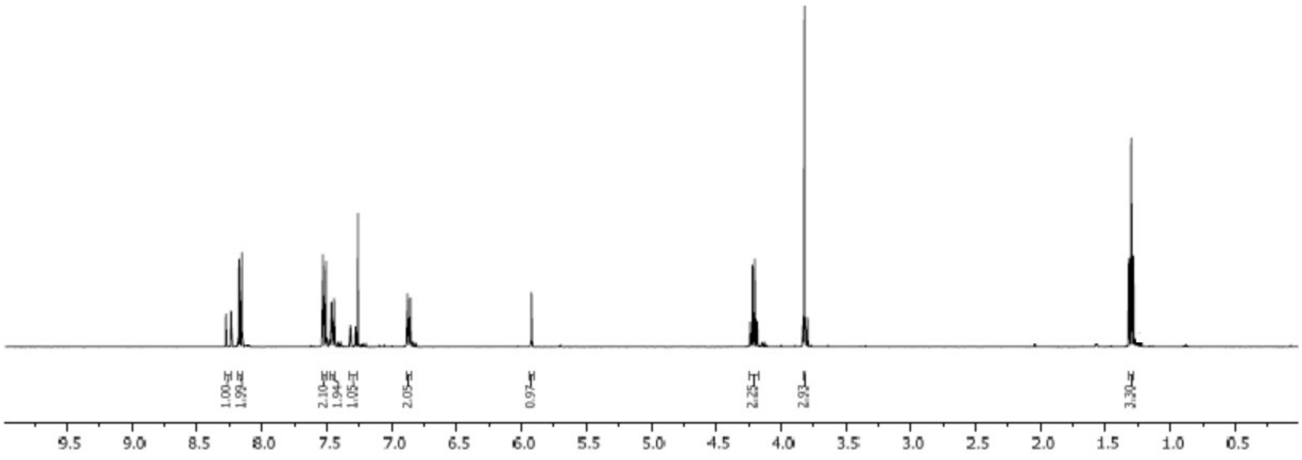

Fig 21. 3k ^{1}H NMR

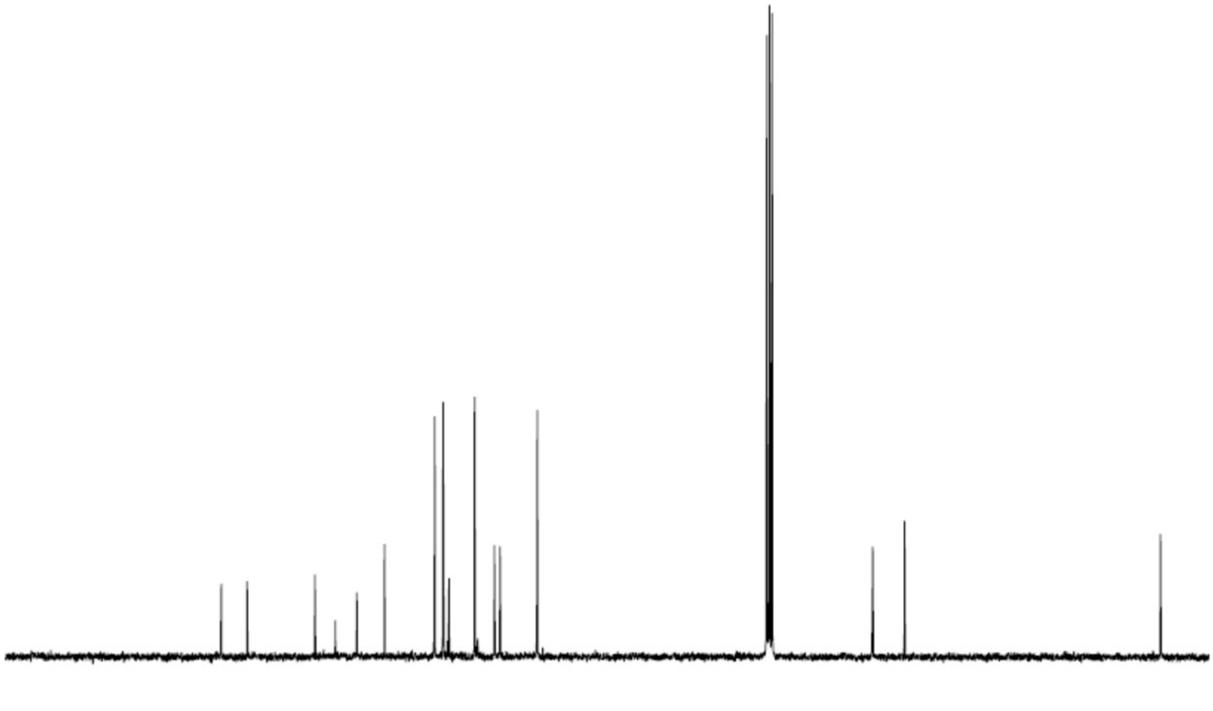

Fig 22. 3k ^{13}C NMR

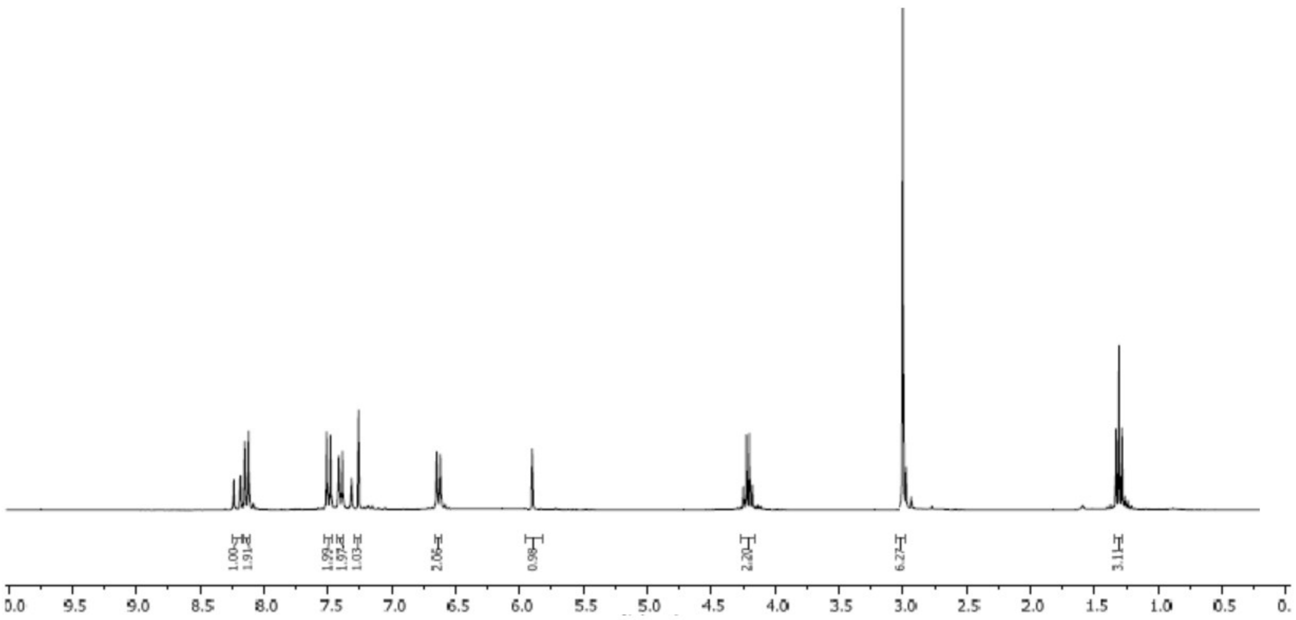

Fig 23. 3l ^{1}H NMR

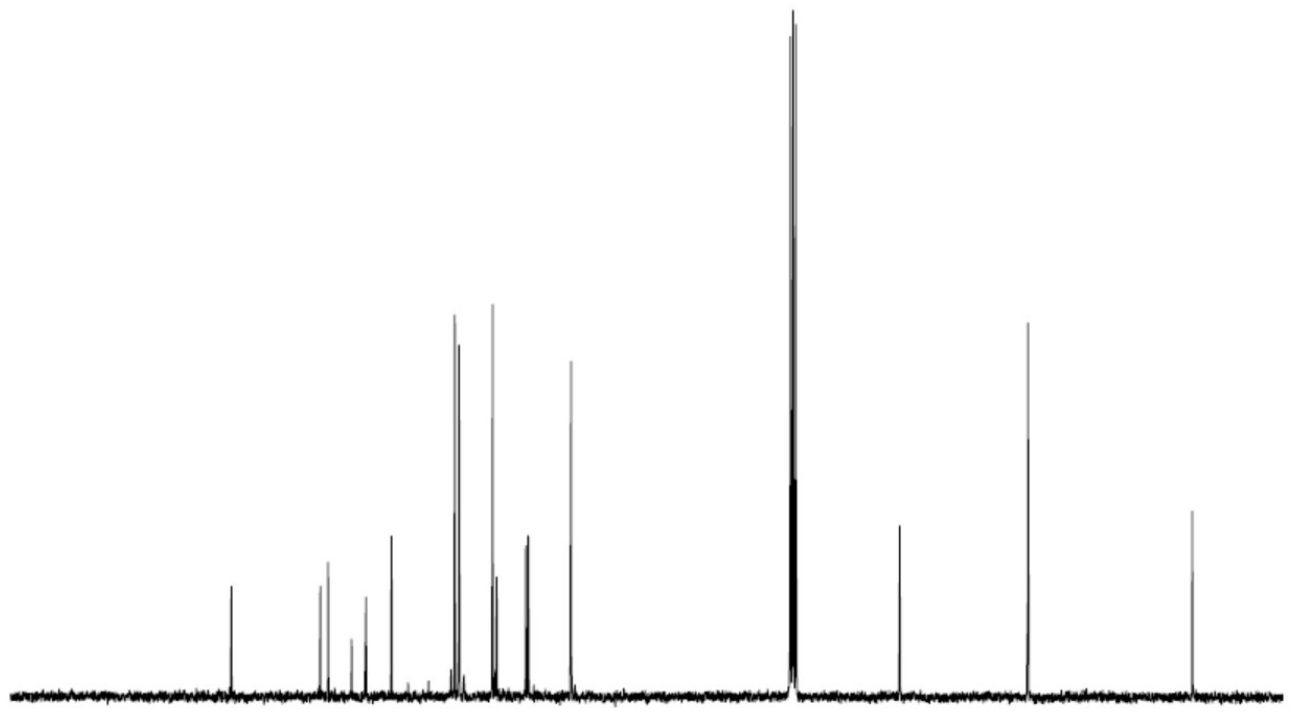

Fig 24. 3l ^{13}C NMR

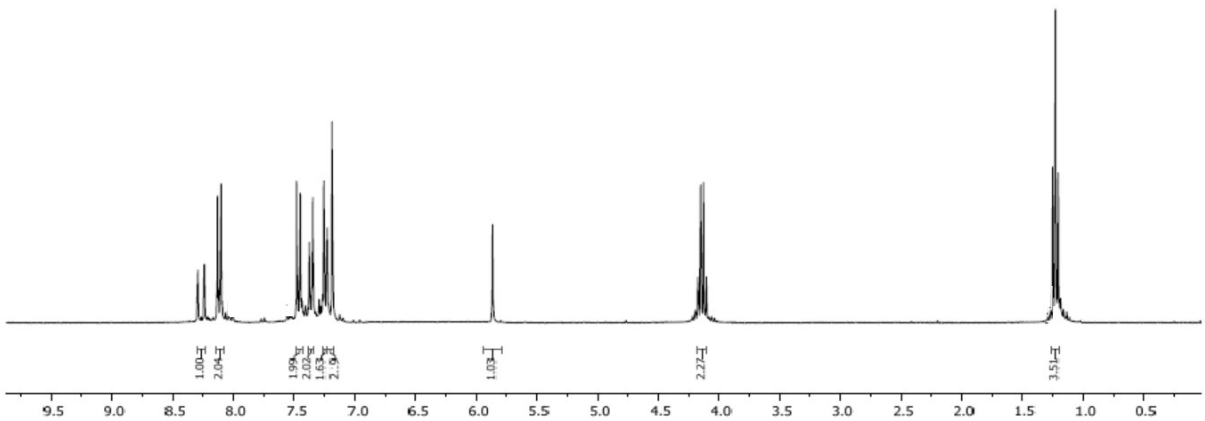

Fig 25. 3m ^{1}H NMR

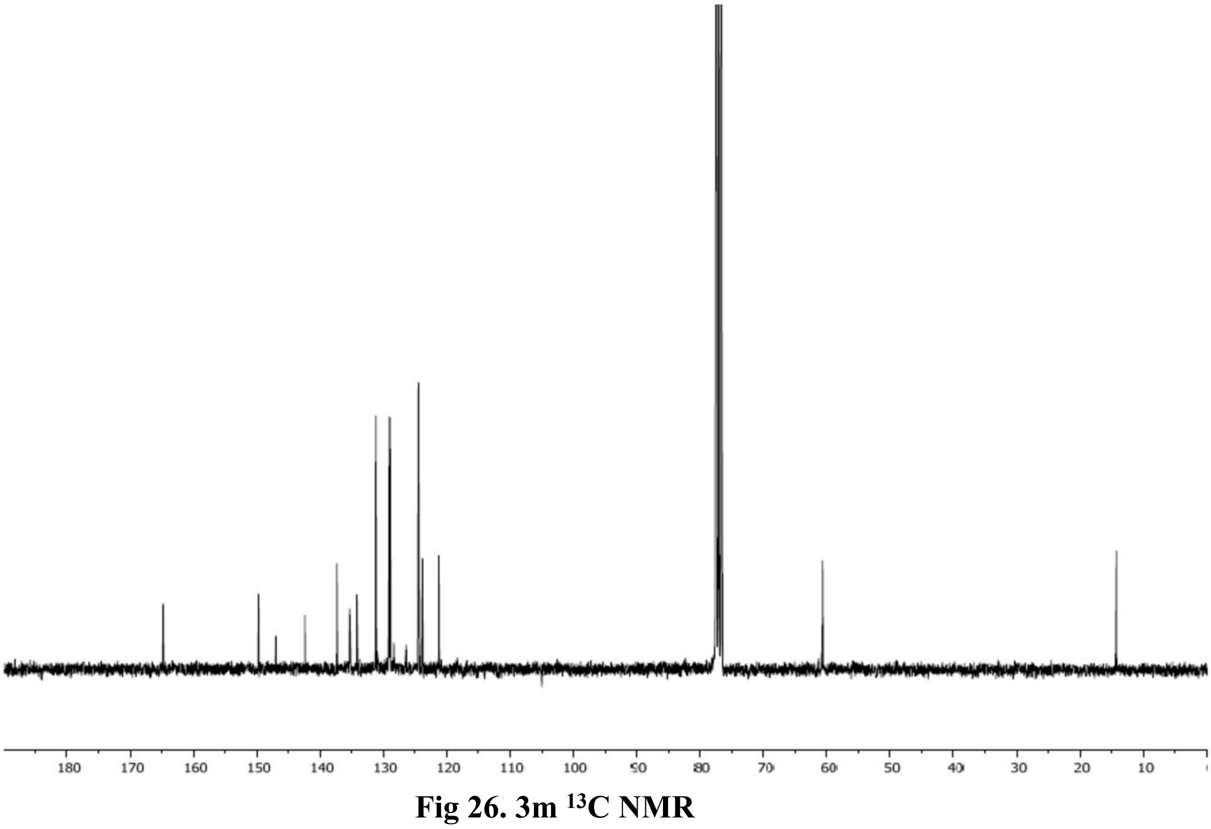

Fig 26. 3m ^{13}C NMR

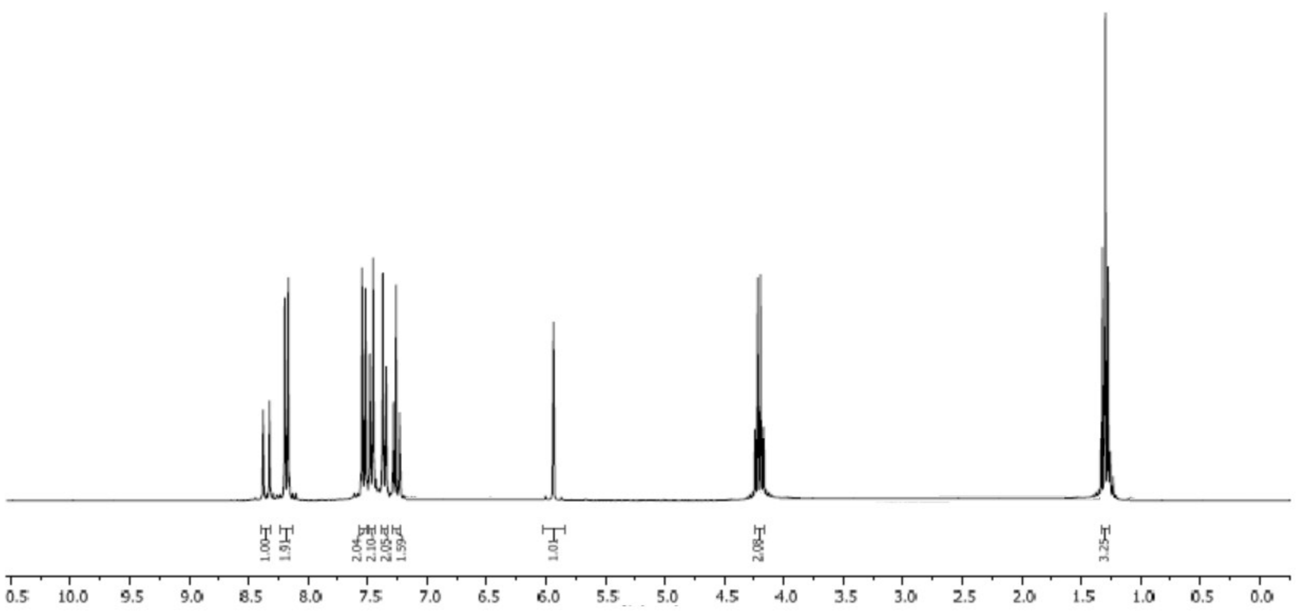

Fig 27. 3n ^{1}H NMR

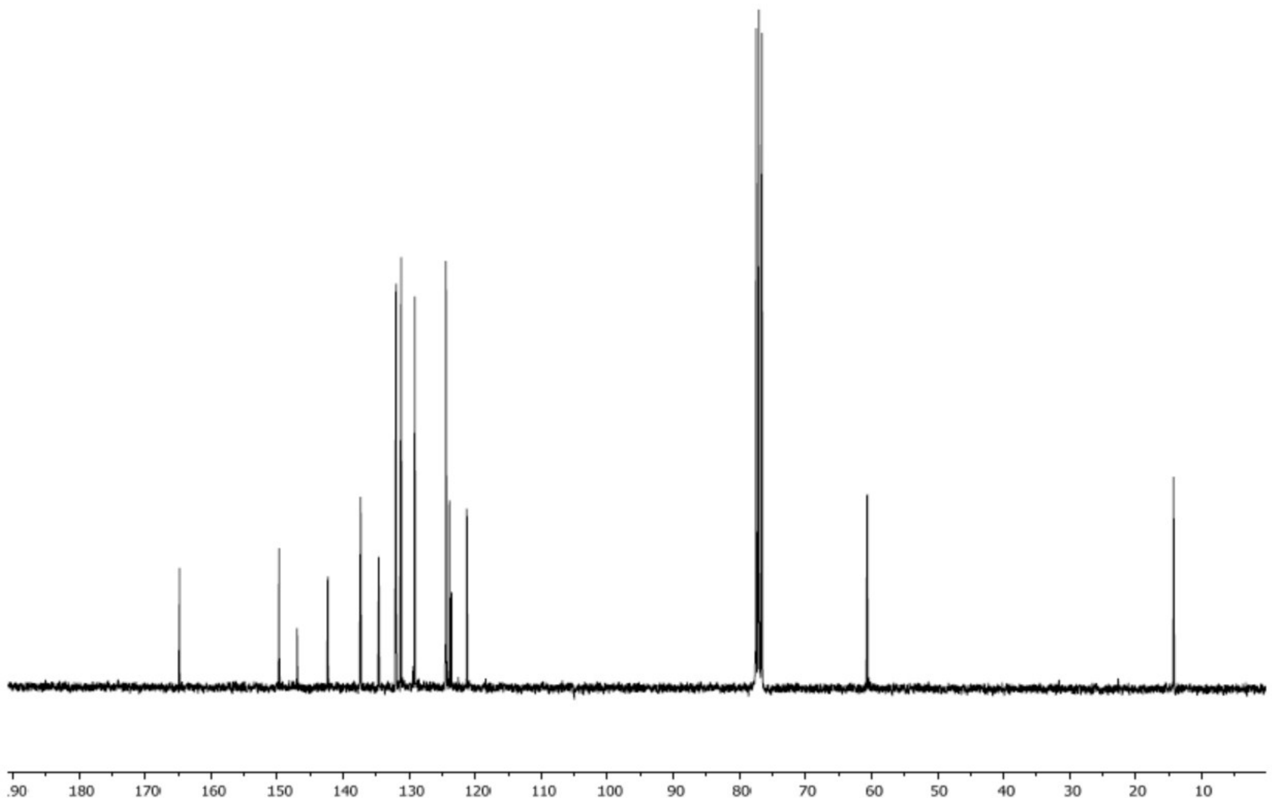

Fig 28. 3n ^{13}C NMR

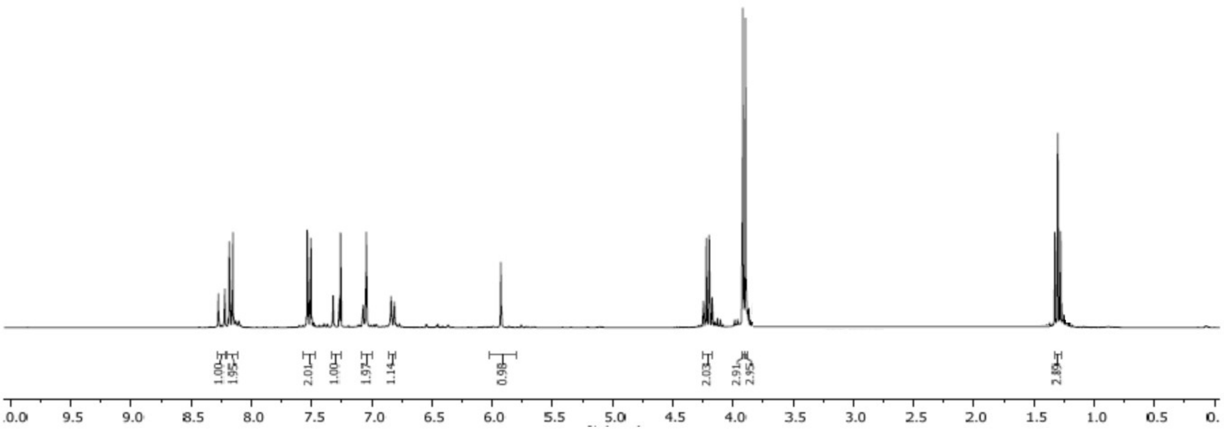

Fig 29. 3o ^{1}H NMR

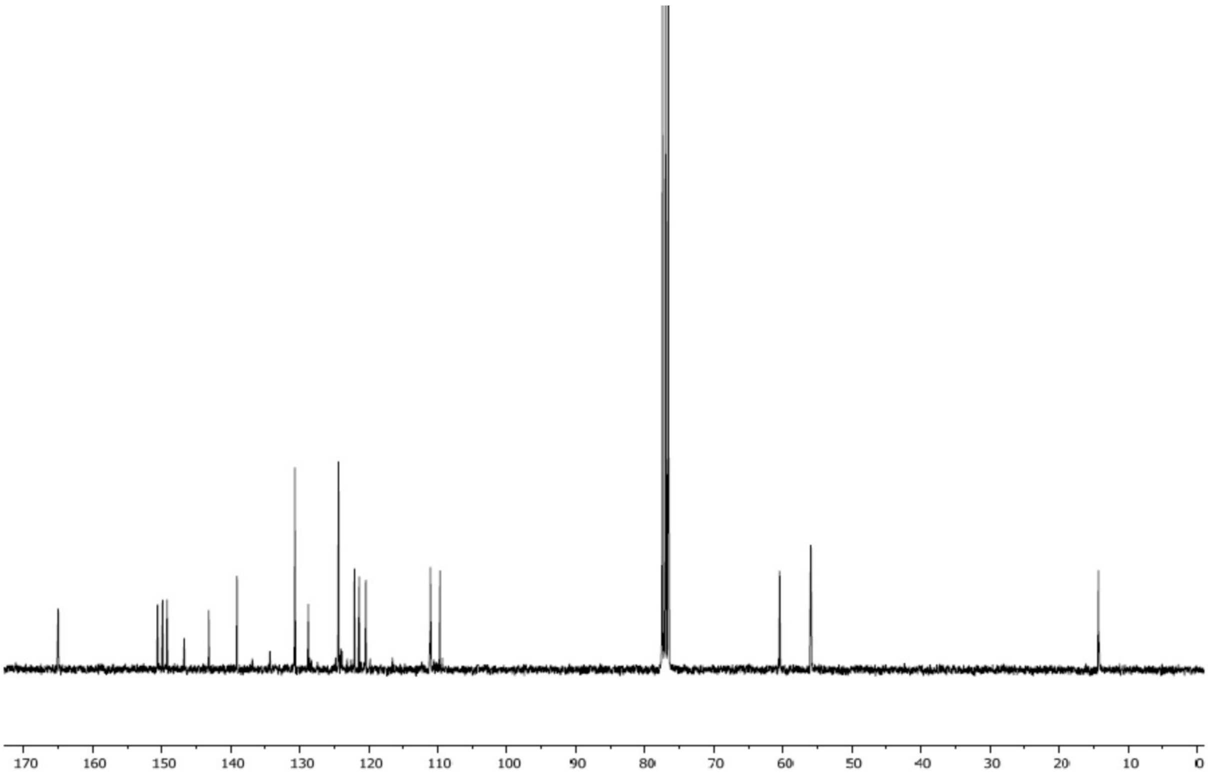

Fig 30. 3o ^{13}C NMR

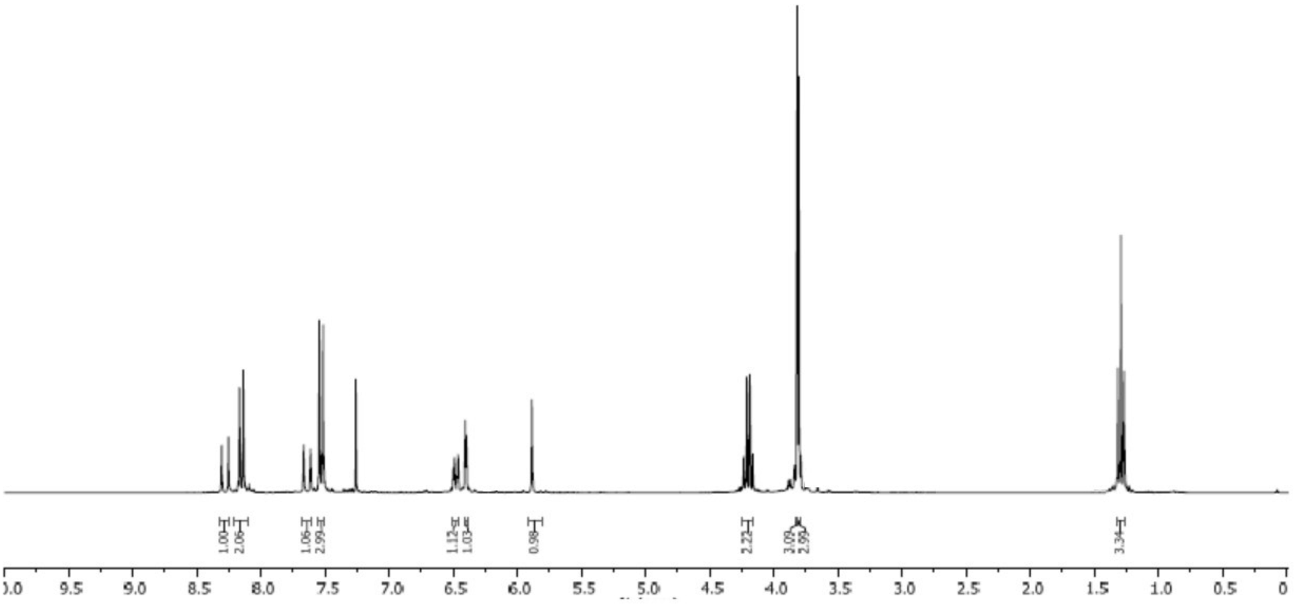

Fig 31. 3p ¹H NMR

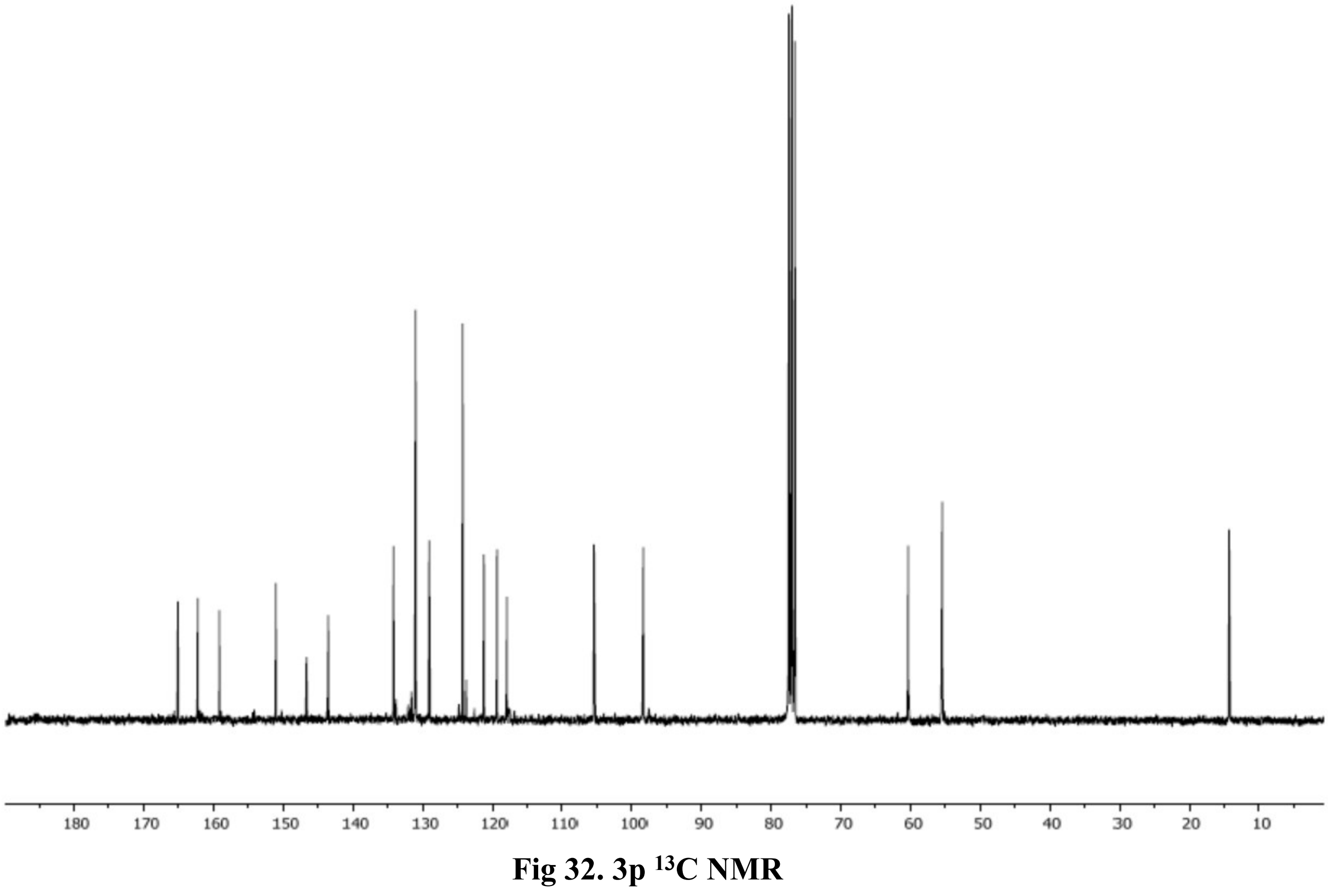

Fig 32. 3p ¹³C NMR

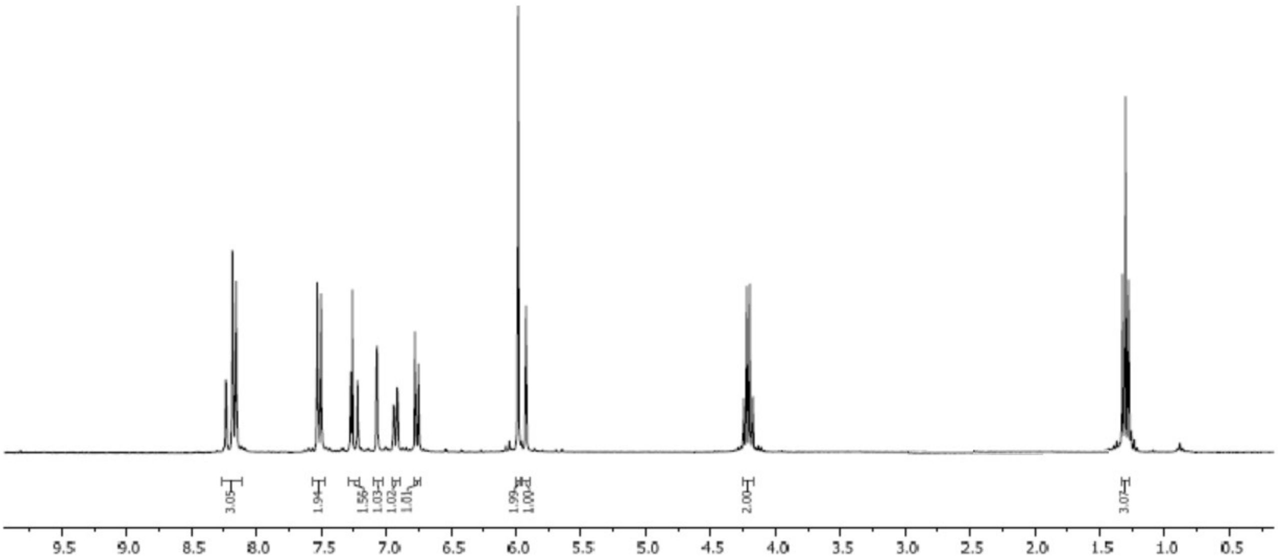

Fig 33. 3q ^{1}H NMR

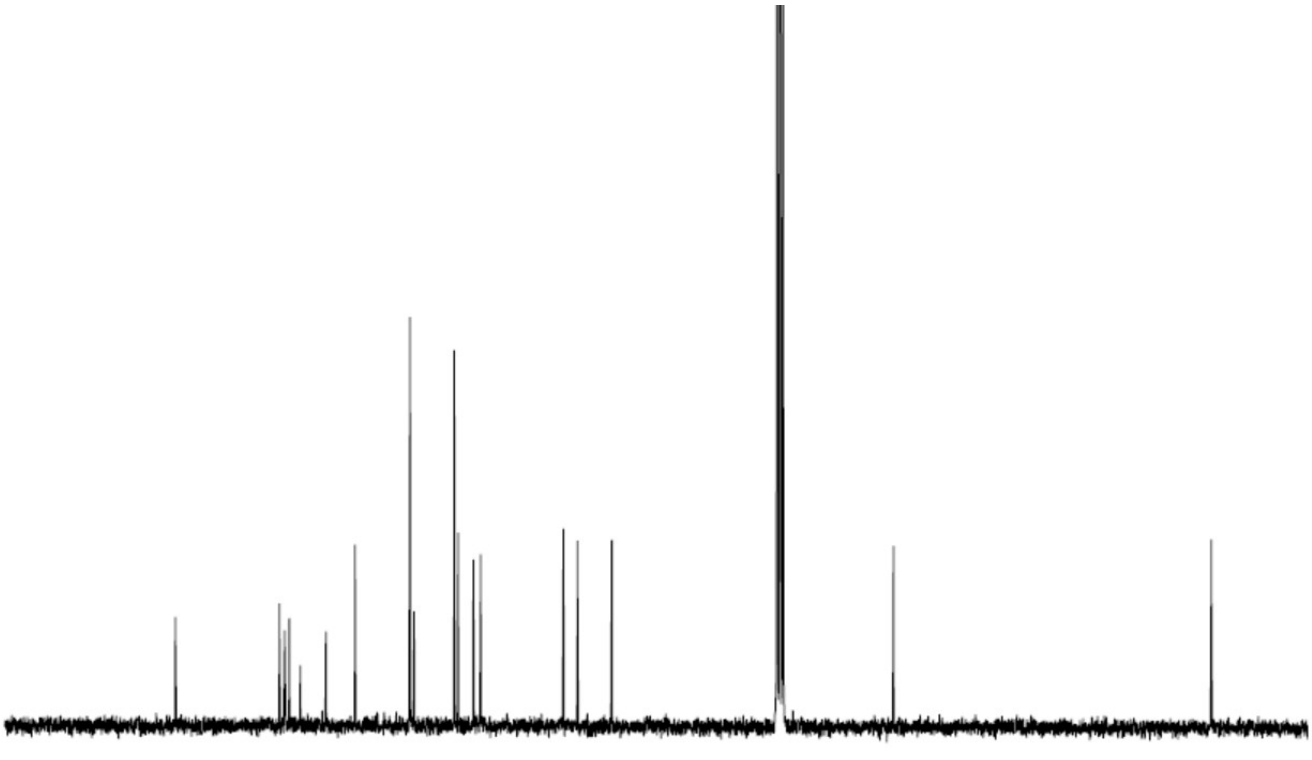

Fig 34. 3q ^{13}C NMR

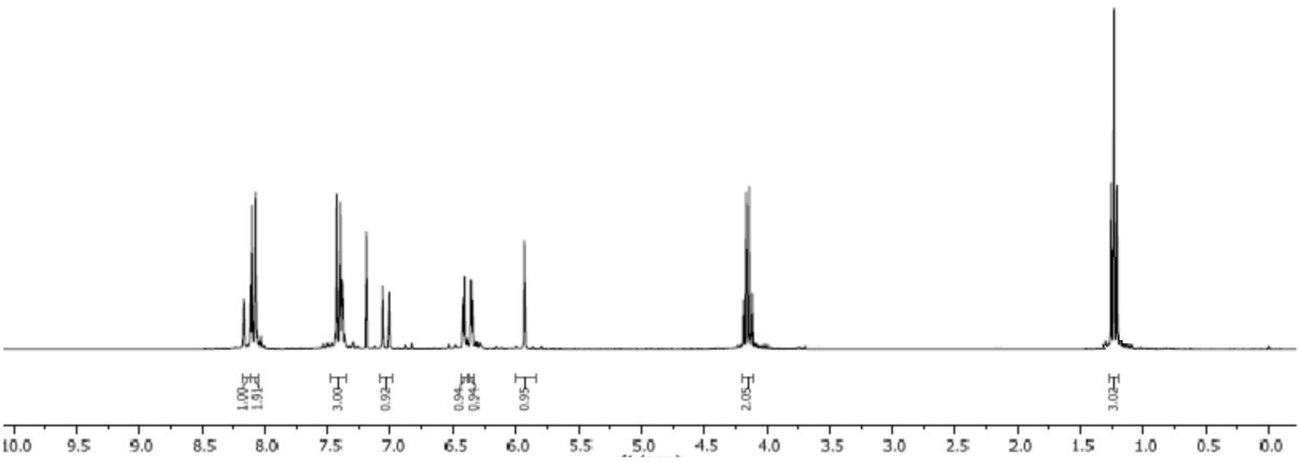

Fig 35. 3r ^{1}H NMR

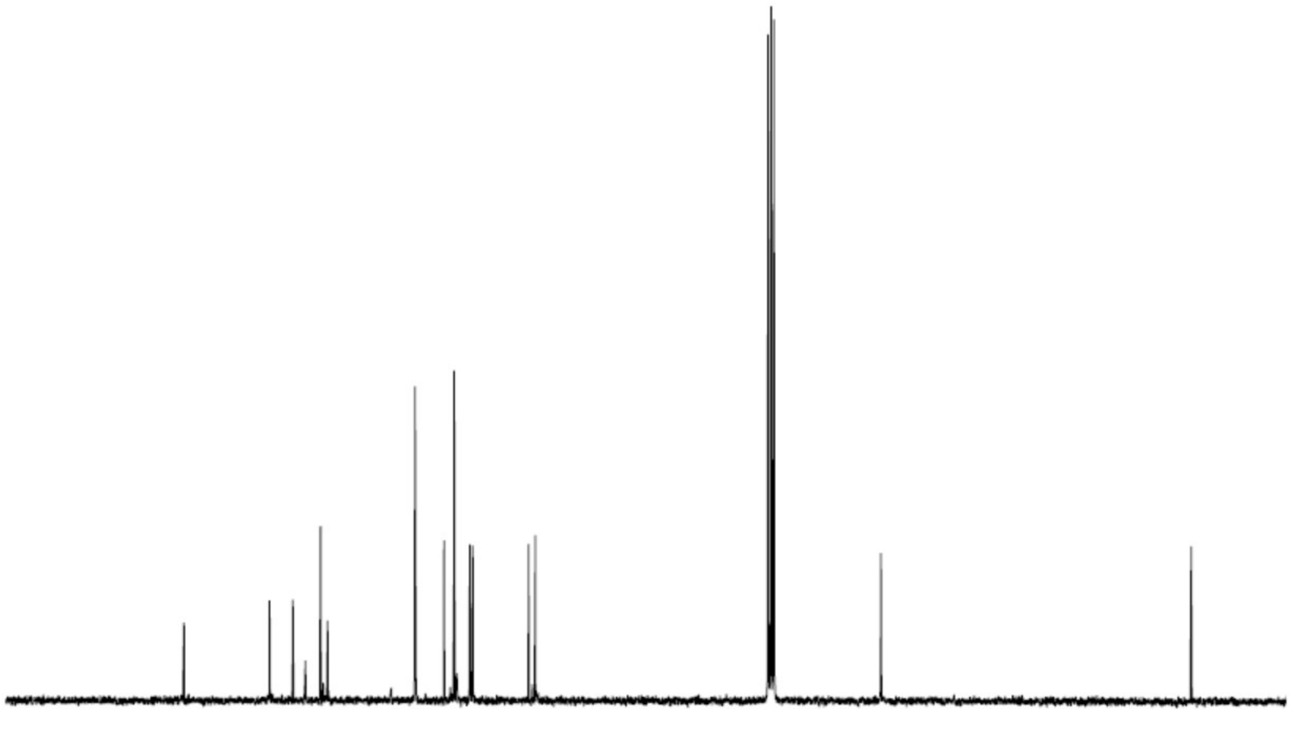

Fig 36. 3r ^{13}C NMR

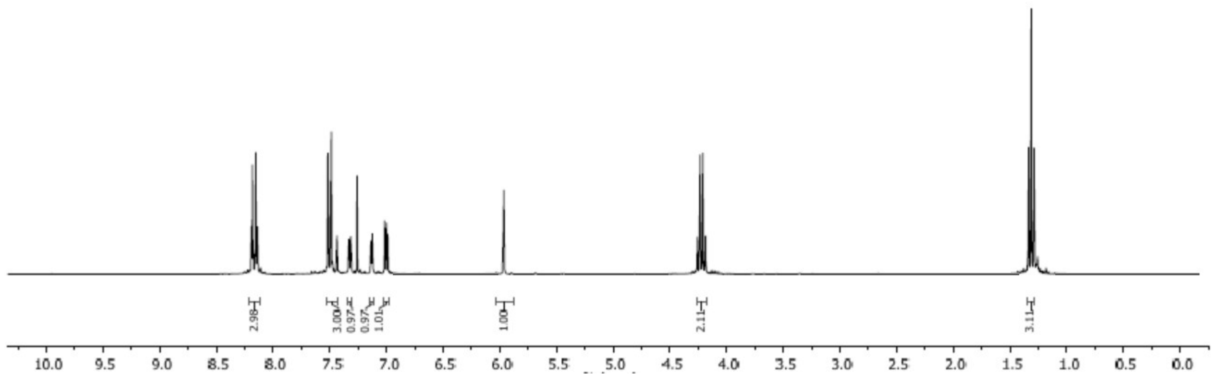

Fig 37. 3s ^{1}H NMR

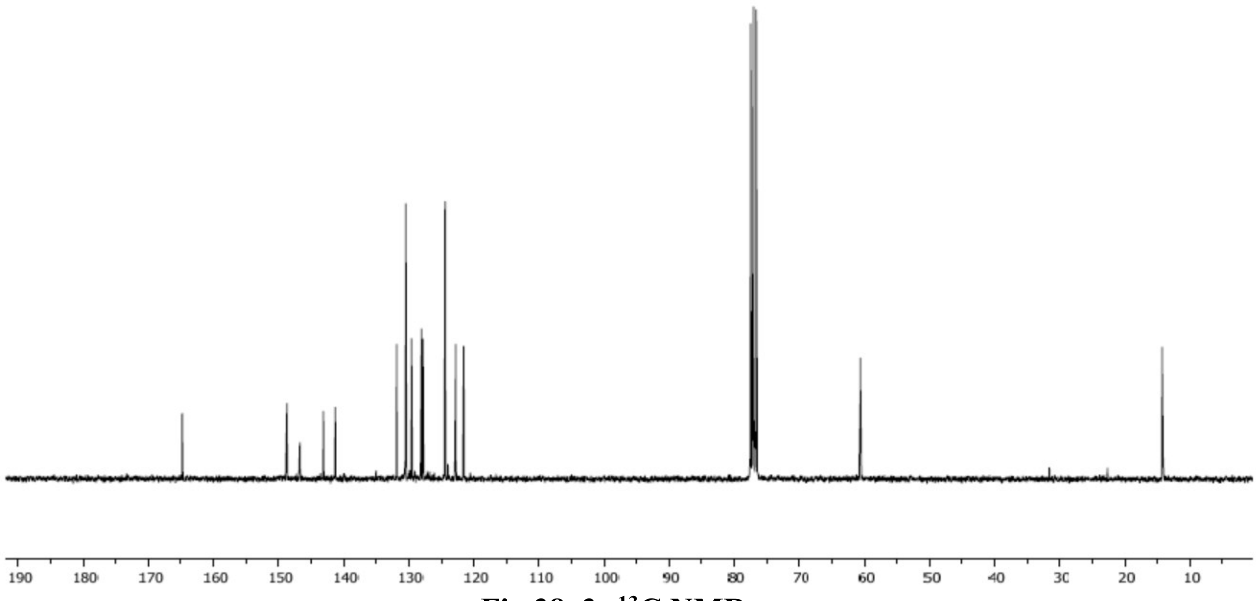

Fig 38. 3s ^{13}C NMR

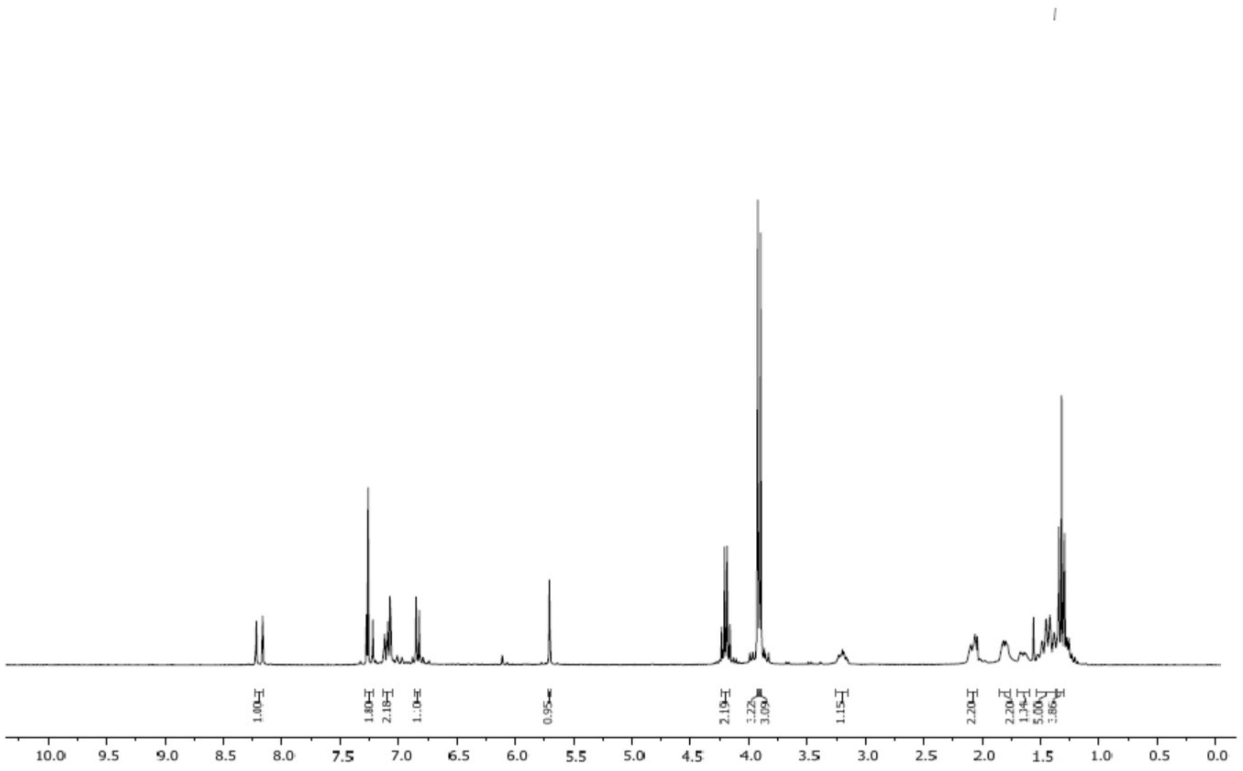

Fig 39. 5a ^{1}H NMR

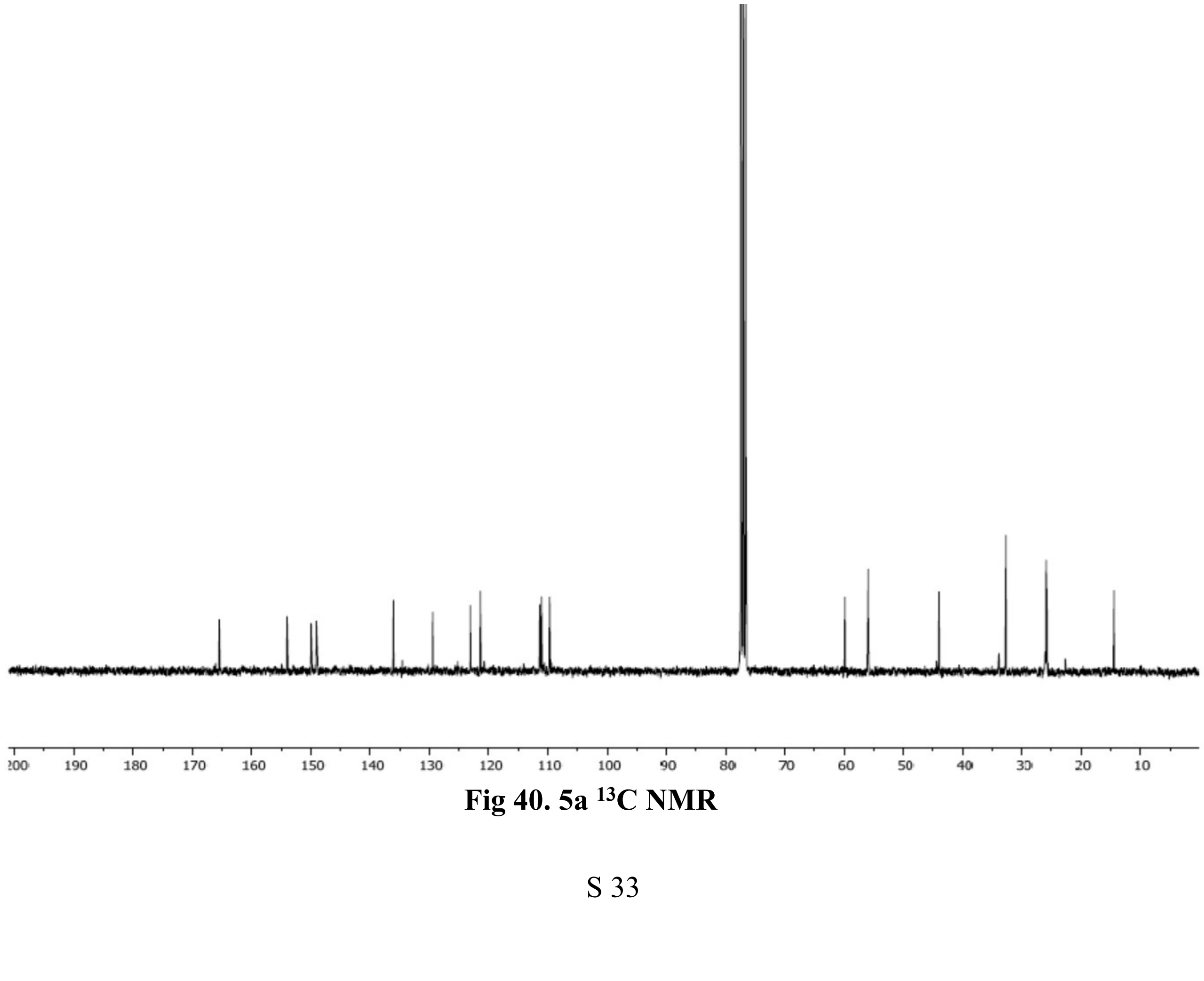

Fig 40. 5a ^{13}C NMR

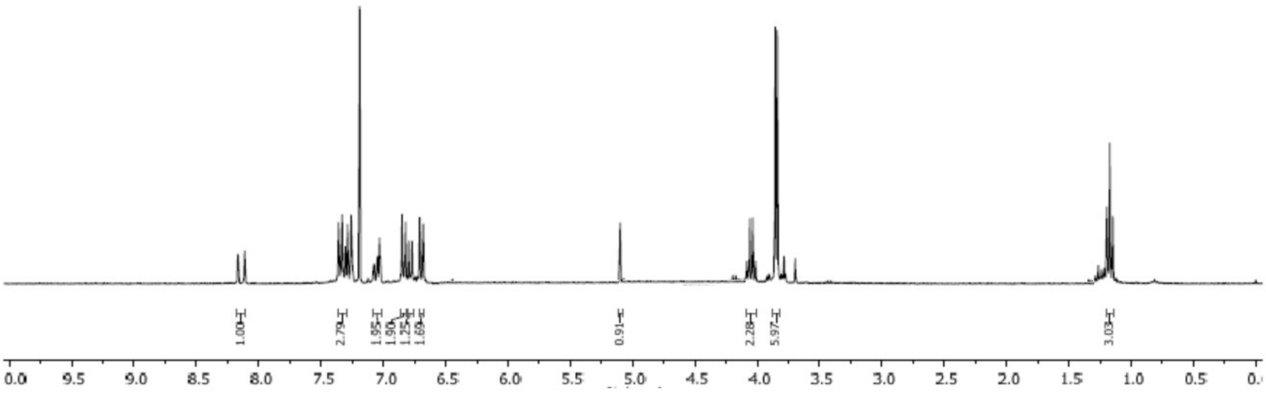

Fig 41. 5b ^{1}H NMR

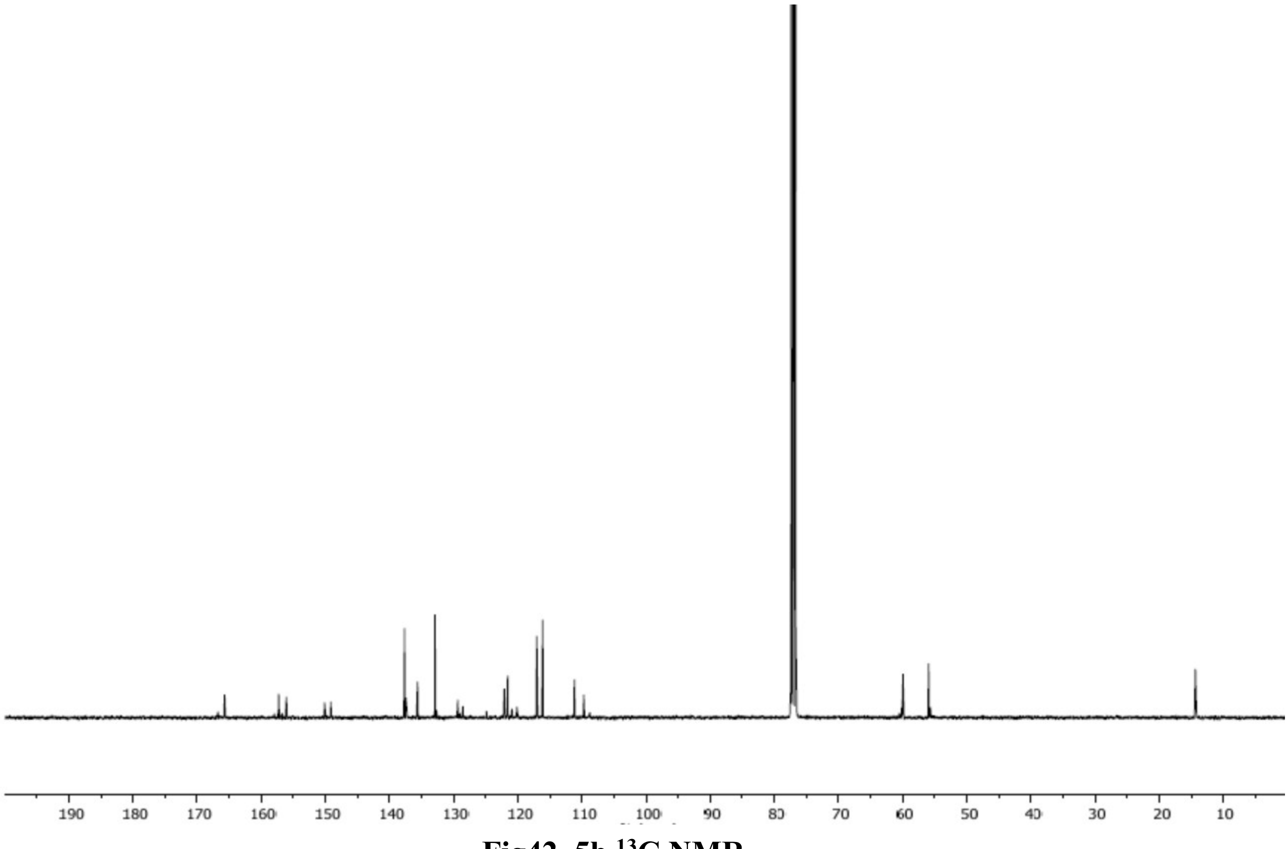

Fig42. 5b ^{13}C NMR

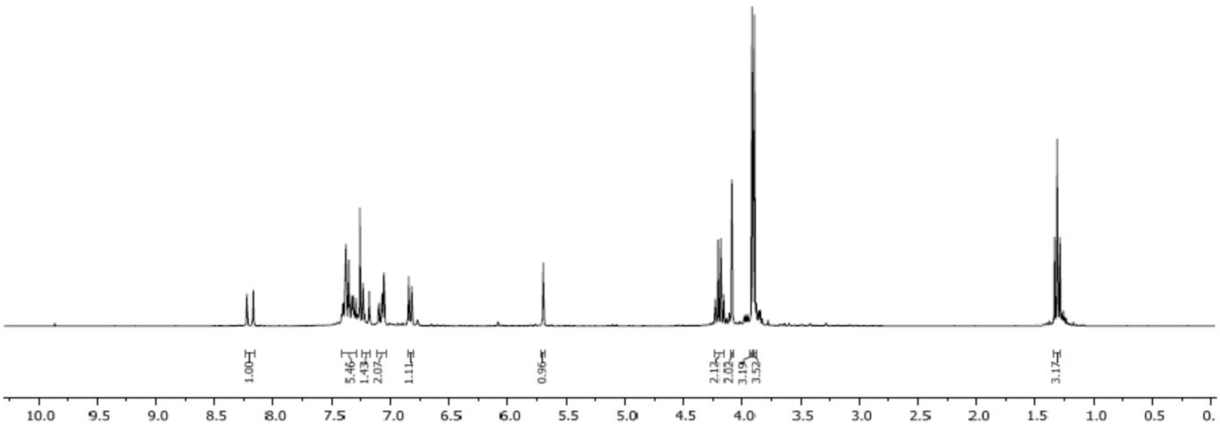

Fig 43. 5c ^{1}H NMR

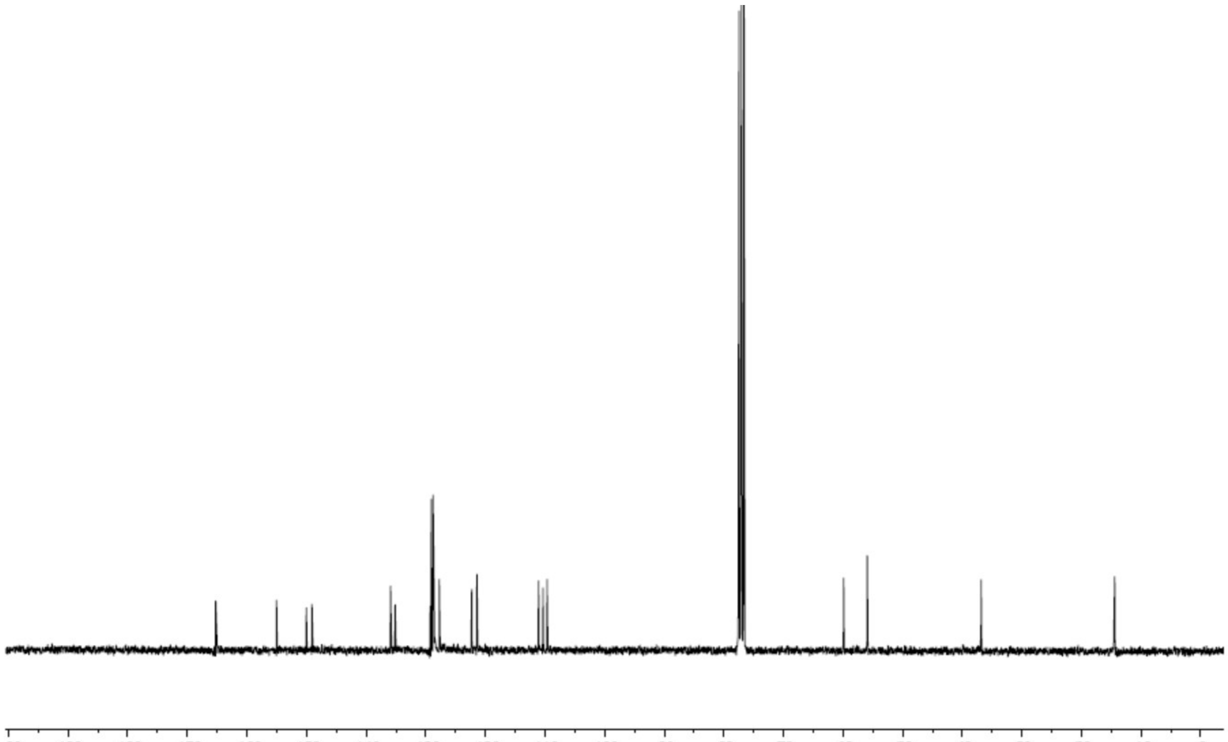

Fig 44. 5c ^{13}C NMR

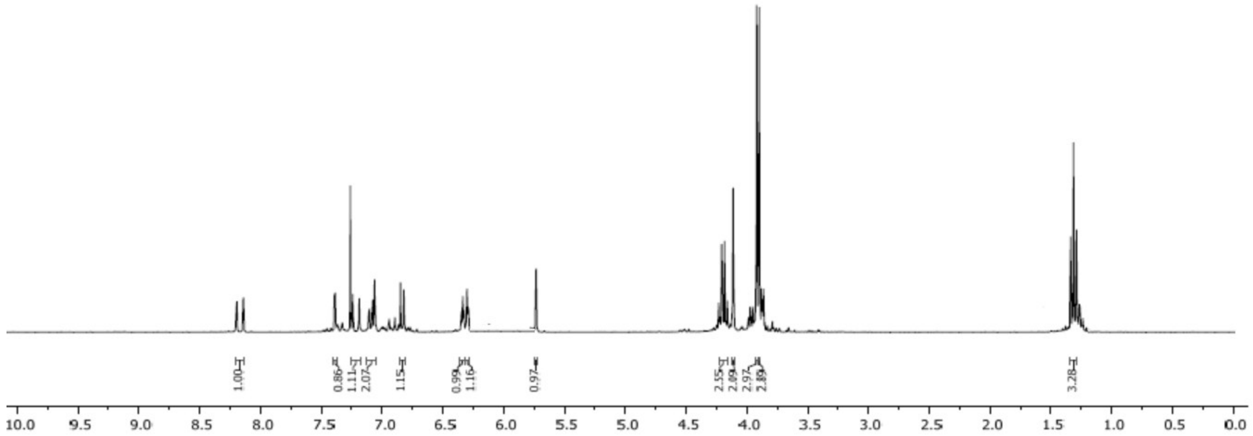

Fig 45. 5d ^{1}H NMR

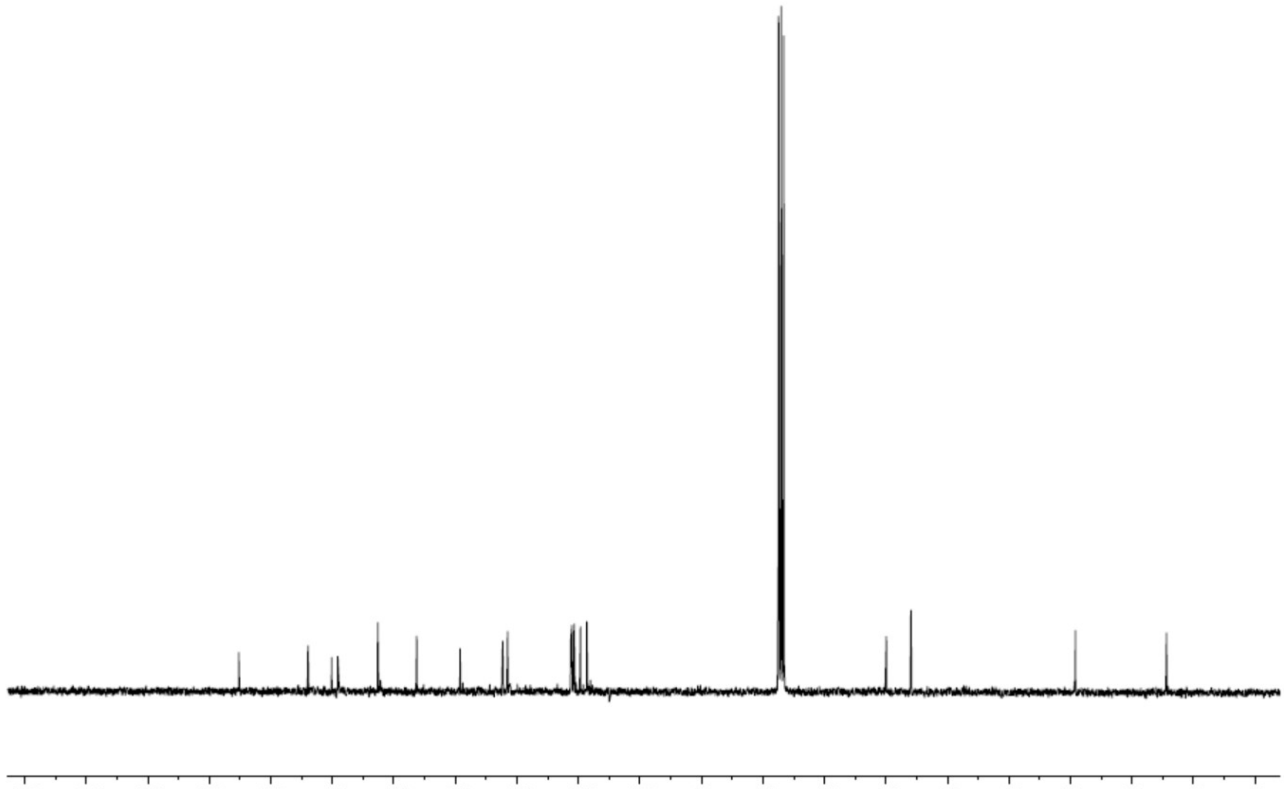

Fig 46. 5d ^{13}C NMR

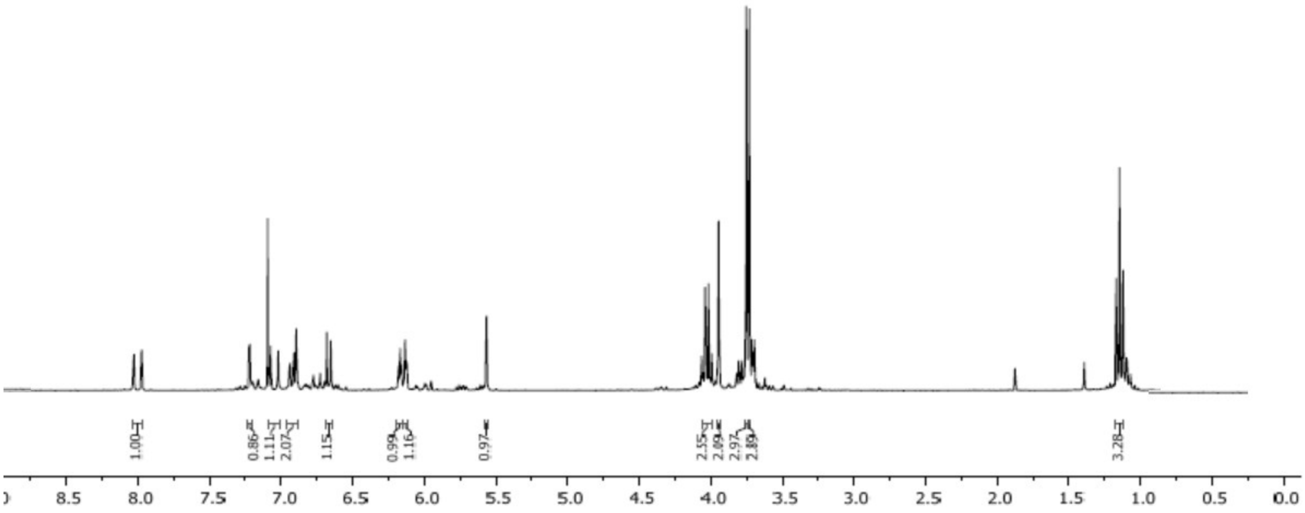

Fig 47. 5e ^{1}H NMR

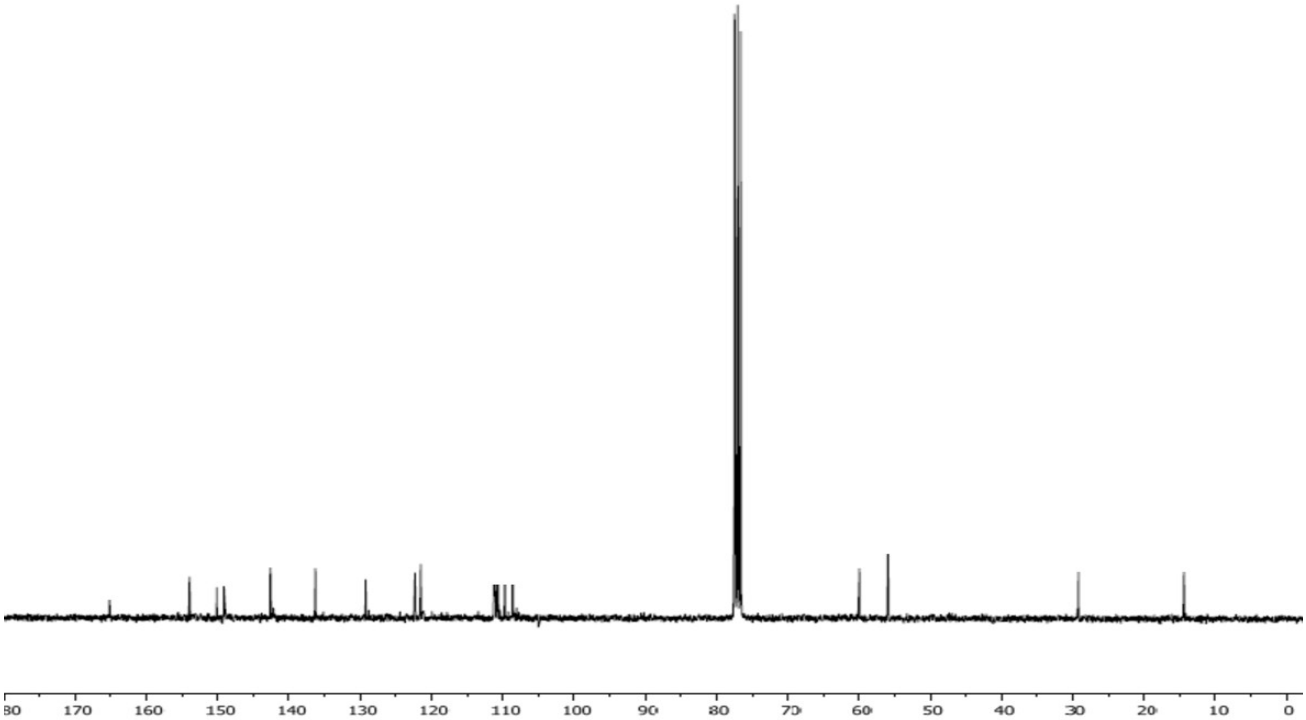

Fig 48. 5e ^{13}C NMR
